# Mechanistic Insights into Dibasic Iminosugars as pH-Selective Pharmacological Chaperones to Stabilize Human α-Galactosidase

**DOI:** 10.1021/jacsau.3c00684

**Published:** 2024-02-23

**Authors:** Huang-Yi Li, Hung-Yi Lin, Sheng-Kai Chang, Yu-Ting Chiu, Chung-Chien Hou, Tzu-Ping Ko, Kai-Fa Huang, Dau-Ming Niu, Wei-Chieh Cheng

**Affiliations:** † Genomics Research Center, Academia Sinica, 128, Section 2, Academia Road, Nankang, Taipei 115201, Taiwan; ‡ Institute of Biochemistry and Molecular Biology, National Yang Ming Chiao Tung University, 155, Section 2, Linong Street, Taipei 112304, Taiwan; § Department of Pediatrics, Taipei Veterans General Hospital, 201, Section 2, Shipai Road, Beitou, Taipei 112201, Taiwan; ∥ Institute of Biological Chemistry, Academia Sinica, 128, Section 2, Academia Road, Nankang, Taipei 11529, Taiwan; ⊥ Institute of Clinical Medicine, School of Medicine, National Yang Ming Chiao Tung University, 155, Section 2, Linong Street, Taipei 112304, Taiwan; # Department of Chemistry, National Cheng Kung University, 1, University Road, East, Tainan 701401, Taiwan; ∇ Department of Chemistry, National University of Kaohsiung, 700, University Road, Nanzih, Kaohsiung 811726, Taiwan; ○ Department of Chemistry, National Chiayi University, 300, Syuefu Road, Chiayi 600355, Taiwan

**Keywords:** pharmacological chaperone, pyrrolidine-based iminosugar, human lysosomal α-galactosidase, Fabry disease, pH-selective binding, electrostatic repulsion

## Abstract

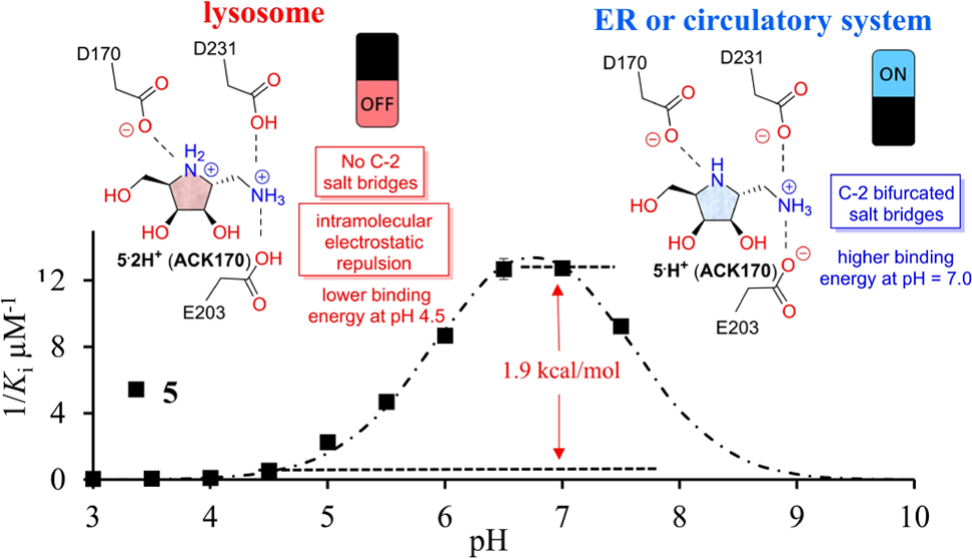

The use of pharmacological chaperones (PCs) to stabilize specific enzymes and impart a therapeutic benefit is an emerging strategy in drug discovery. However, designing molecules that can bind optimally to their targets at physiological pH remains a major challenge. Our previous study found that dibasic polyhydroxylated pyrrolidine **5** exhibited superior pH-selective inhibitory activity and chaperoning activity for human α-galactosidase A (α-Gal A) compared with its monobasic parent molecule, **4**. To further investigate the role of different C-2 moieties on the pH-selectivity and protecting effects of these compounds, we designed and synthesized a library of monobasic and dibasic iminosugars, screened them for α-Gal A-stabilizing activity using thermal shift and heat-induced denaturation assays, and characterized the mechanistic basis for this stabilization using X-ray crystallography and binding assays. We noted that the dibasic iminosugars **5** and **20** protect α-Gal A from denaturation and inactivation at lower concentrations than monobasic or other *N*-substituted derivatives; a finding attributed to the nitrogen on the C-2 methylene of **5** and **20**, which forms the bifurcated salt bridges (BSBs) with two carboxyl residues, E203 and D231. Additionally, the formation of BSBs at pH 7.0 and the electrostatic repulsion between the vicinal ammonium cations of dibasic iminosugars at pH 4.5 are responsible for their pH-selective binding to α-Gal A. Moreover, compounds **5** and **20** demonstrated promising results in improving enzyme replacement therapy and exhibited significant chaperoning effects in Fabry cells. These findings suggest amino-iminosugars **5** and **20** as useful models to demonstrate how an additional exocyclic amino group can improve their pH-selectivity and protecting effects, providing new insights for the design of pH-selective PCs.

## Introduction

α-Galactosidase A (α-Gal A, EC 3.2.1.22) is a lysosomal glycosidase that hydrolyzes the terminal α-linked galactoside present on macromolecules such as globotriaosylceramide (Gb3).^1,2^ Mutations in the α-galactosidase A gene (*GLA*) are found in patients with Fabry disease (FD), one of the most common lysosomal storage diseases (LSDs), which is characterized by the progressive accumulation of Gb3 in cells.^3,4^ Normal lysosomal enzymes fold in the endoplasmic reticulum (ER) and are then transported to the lysosomes, but mutant enzymes fail to fold correctly in the ER and are degraded by the ER quality control system.^5−7^ The current standard of care for LSDs is enzyme replacement therapy (ERT), where intravenous (IV) infusions of exogenous recombinant enzymes are used to restore the function of lysosomes in patient cells.^8−10^

**Figure 1:**
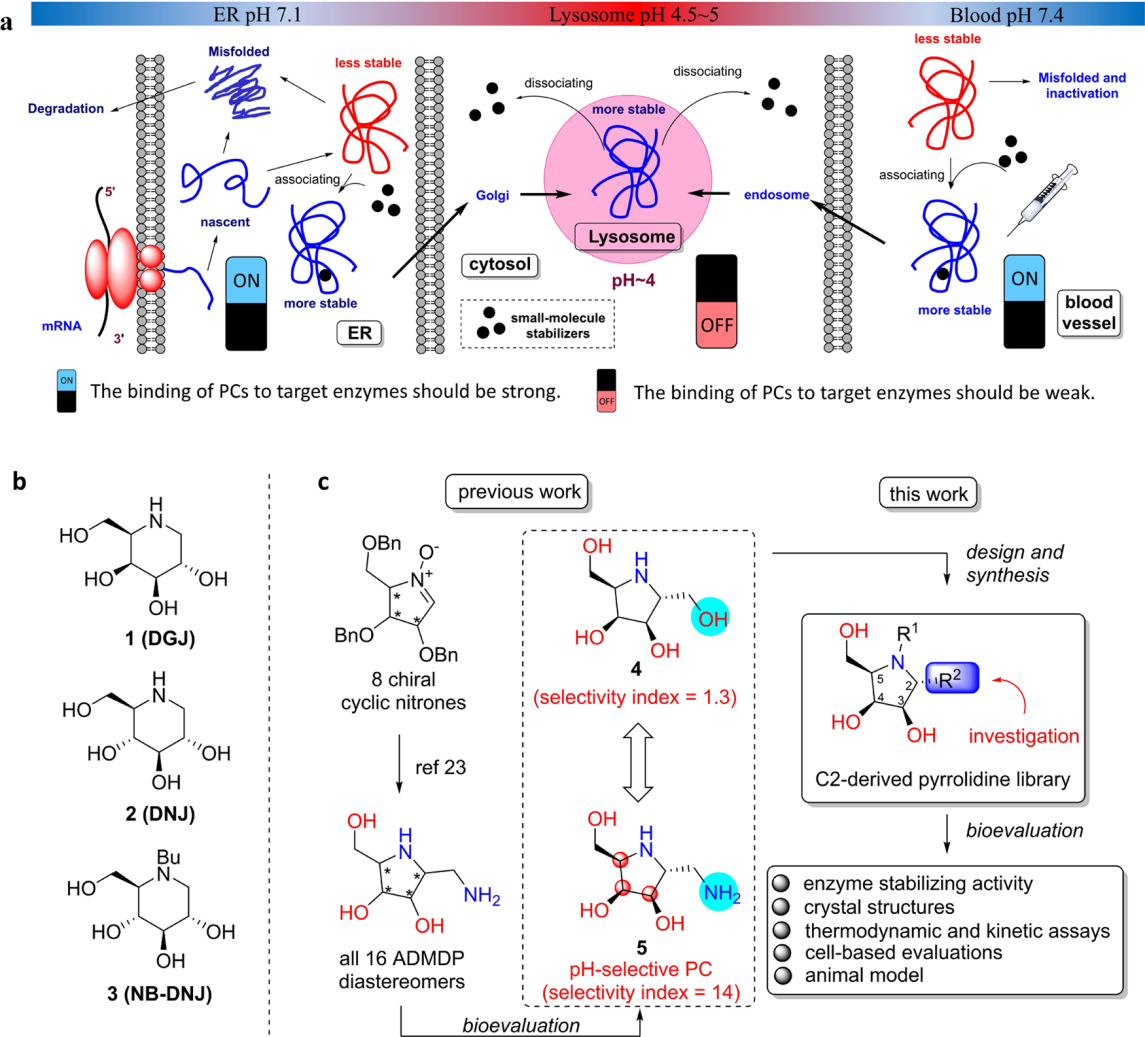
Iminosugars to treat LSDs. (a) pH-dependent binding of PCs toward partner enzymes. (b) Structures of iminosugar-based PCs. (c) Strategy to investigate the effect of different C-2 moieties on the chaperoning activity of iminosugars to α-Gal A.

More recently, pharmacological chaperone therapy (PCT), the use of pharmacological chaperones (PCs) to either enhance the trafficking of endogenous mutant proteins to the lysosome and/or minimize the denaturation of exogenous recombinant enzymes in the circulatory system, has emerged as an alternative approach to treat FD and many other LSDs (Figure 1a).^11−14^ For example, 1-deoxygalactonojirimycin (DGJ, **1**) is the first PC approved by the U.S. Food and Drug Administration (FDA) and European Medicines Agency (EMA) for treating patients with FD.^15,16^ Most reported PCs such as the iminosugars DGJ and 1-deoxynojirimycin (DNJ, **2**) are competitive inhibitors since PC development starting from glycosidase substrates or transition-state mimics is easier than starting from scratch (Figure 1b).^17−19^ However, the inhibitory activity of these PCs can diminish their therapeutic benefit. Several strategies have been developed to reduce their inhibitory effects, such as the use of inhibitors that decompose in the acidic environment within lysosomes or transient inactivators that can be slowly hydrolyzed by their target enzymes.^20−22^ A major challenge associated with the development of PCs to treat LSDs is the need for their binding to be pH-dependent; the PCs should preferentially bind to their targets in the ER (pH 7.1) and the circulatory systems (pH 7.4) rather than in lysosomes (pH 4.5–5.0), where substrate processing should not be inhibited.

Previously, the dibasic iminosugar and potent α-Gal A inhibitor **5** having the (2*R*,3*R*,4*S*,5*R*) configuration pattern demonstrated good chaperoning activity in cells transiently transfected with various mutant α-Gal A.^23^ Notably, iminosugar **5** exhibited superior pH-dependence in its inhibitory activity compared to its monobasic parent molecule, **4** (Figure 1c), suggesting that it binds to α-Gal A at pH 7.0 more effectively than **4**, leading to better enzyme-stabilizing or chaperoning activity. The lower IC_50_ of **5** (IC_50_ = 53 nM) against α-Gal A at pH 7.0 than pH 4.6 (IC_50_ = 670 nM) suggests that **5** can tightly bind to the defective α-Gal A in the ER but not in the lysosomes.^23^ Despite the structural similarity between compounds **4** and **5**, which differ only in the heteroatoms present on the C-2 methylene, the underlying mechanism by which the additional amino moiety in **5** enhances its binding potency to α-Gal A at pH 7 remains unclear; thus, understanding how different C-2 moieties, particularly amino groups, contribute to the pH-selectivity and enzyme-stabilizing activity of iminosugars could provide valuable insights for the development of PCs to treat LSDs.

Herein, we report the design and synthesis of a series of monobasic and dibasic pyrrolidine-based iminosugars based on natural product-inspired combinatorial chemistry (NPICC) to elucidate how the structural changes in the C-2 moiety of the iminosugars affect α-Gal A stabilization (Figure 1c).^100^ The enzyme-stabilizing activity of these iminosugars will be examined by thermal shift and heat-induced denaturation assays, and the molecular basis for the interactions between the iminosugars and α-Gal A revealed by X-ray crystal structures and binding assays. Finally, the protecting effects of these iminosugars will be evaluated in Fabry cells and mouse models.

## Results and Discussion

### Design and Synthesis of Monobasic and Dibasic Iminosugars

**Scheme 1:**
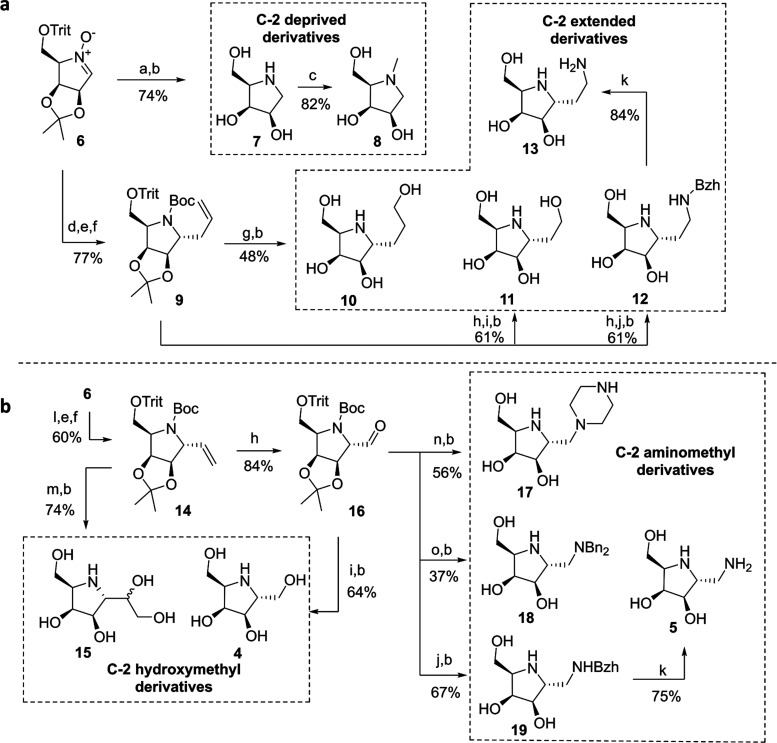
Synthesis of Pyrrolidine-Based Iminosugars (a) Synthesis of C-2-deprived and C-2-extended derivatives. (b) Synthesis of C-2 hydroxymethyl and aminomethyl derivatives. Reagents and conditions: (a) Raney Ni, H_2_, MeOH, rt, 12 h. (b) 6 N HCl, MeOH, rt, 12 h; then DOWEX (OH^–^). (c) H_2_, Pd(OH)_2_/C, CH_2_O, MeOH, rt, 12 h. (d) allylMgCl, THF, 0 °C, 2 h. (e) Zn, AcOH, CH_2_Cl_2_, rt, 12 h. (f) Boc_2_O, Et_3_N, MeOH, rt, 12 h. (g) BH_3_, THF, rt, 1 h; then H_2_O_2_, NaOH, rt, 12 h. (h) O_3_, CH_2_Cl_2_, −78 °C, 0.5 h, then Me_2_S. (i) NaBH_4_, MeOH, rt, 12 h. (j) NaBH_3_CN, NH_2_Bzh, AcOH, MeOH, rt, 12 h. (k) Pd(OH)_2_/C, H_2_, MeOH, rt, 12 h. (l) vinylMgBr, THF, 0 °C, 2 h. (m) OsO_4_, *N*-methylmorpholine *N*-oxide, THF/H_2_O, rt, 12 h. (n) NaBH_3_CN, 1-Boc-piperazine, AcOH, rt, 12 h. (o) NaBH_3_CN, NHBn_2_, AcOH, MeOH, rt, 12 h.

Having identified the (2*R*,3*R*,4*S*,5*R*) configuration pattern as being critical for the chaperoning activity of pyrrolidine-based iminosugars to α-Gal A,^23^ we aimed to investigate the impact of different C-2 moieties on the pH-selective binding and enzyme-stabilizing activity of these molecules. Accordingly, we designed and synthesized four series of iminosugars: (1) C-2-deprived, (2) C-2-extended, (3) C-2 hydroxymethyl, and (4) C-2 aminomethyl iminosugar derivatives (Scheme 1).

To efficiently synthesize C-2-modified iminosugars, a divergent synthetic strategy based on several common intermediates was used. The first of these was cyclic nitrone **6**, prepared from l-ribose as previously reported.^24^ The C-2-deprived pyrrolidines were directly prepared from **6** via reduction of the nitrone moiety followed by acidic deprotection to give **7**, which underwent reductive amination with formaldehyde and Raney Ni under a hydrogen atmosphere to give the *N*-methylated derivative **8** (Scheme 1a).

The C-2-extended, hydroxymethyl, and aminomethyl derivatives were also obtained from **6** via diastereoselective nucleophilic addition of Grignard reagents (Scheme 1a).^25−27^ C-2-extended molecules were prepared from **6** via its allylation, followed by reduction of the hydroxylamine, and *t*-butyl carbamate formation to give alkene **9**. Hydroboration/oxidation of alkene **9** gave hydroxypropyl pyrrolidine **10** after acidic deprotection. Hydroxyethyl and aminoethlyl pyrrolidines **11** and **12** were obtained from **9** via ozonolysis, followed by reduction and reductive amination, respectively. Dibasic iminosugar **13** was obtained by the catalytic hydrogenolysis of **12**.

C-2 hydroxymethyl derivatives were synthesized from alkene **14** (Scheme 1b), which was obtained from **6** in a vinylation, reduction, and protection sequence. Dihydroxylation of **14** by osmium tetroxide followed by acidic deprotection afforded a 1:1 mixture of inseparable diastereomers **15**. Monobasic iminosugar **4** was obtained by ozonolysis of **14**, followed by reduction and deprotection.

Finally, the synthesis of C-2 aminoethyl derivatives commenced by reductive amination of **16**, which is obtained from **14** by ozonolysis (Scheme 1b). Previous studies have proposed that hydrophobic interactions may play a role in enhancing the protecting effect of iminosugars on α-Gal A.^28,29^ To investigate this prospect, reductive amination reactions were conducted with different alkylamines, affording *N*-alkylated amino-iminosugars **17**–**19** after deprotection.

### Protecting Effects of Iminosugars **4**, **5**, **7**, **8**, **10**–**13**, **15**, **and 17**–**19** on α-Gal A

**Figure 2:**
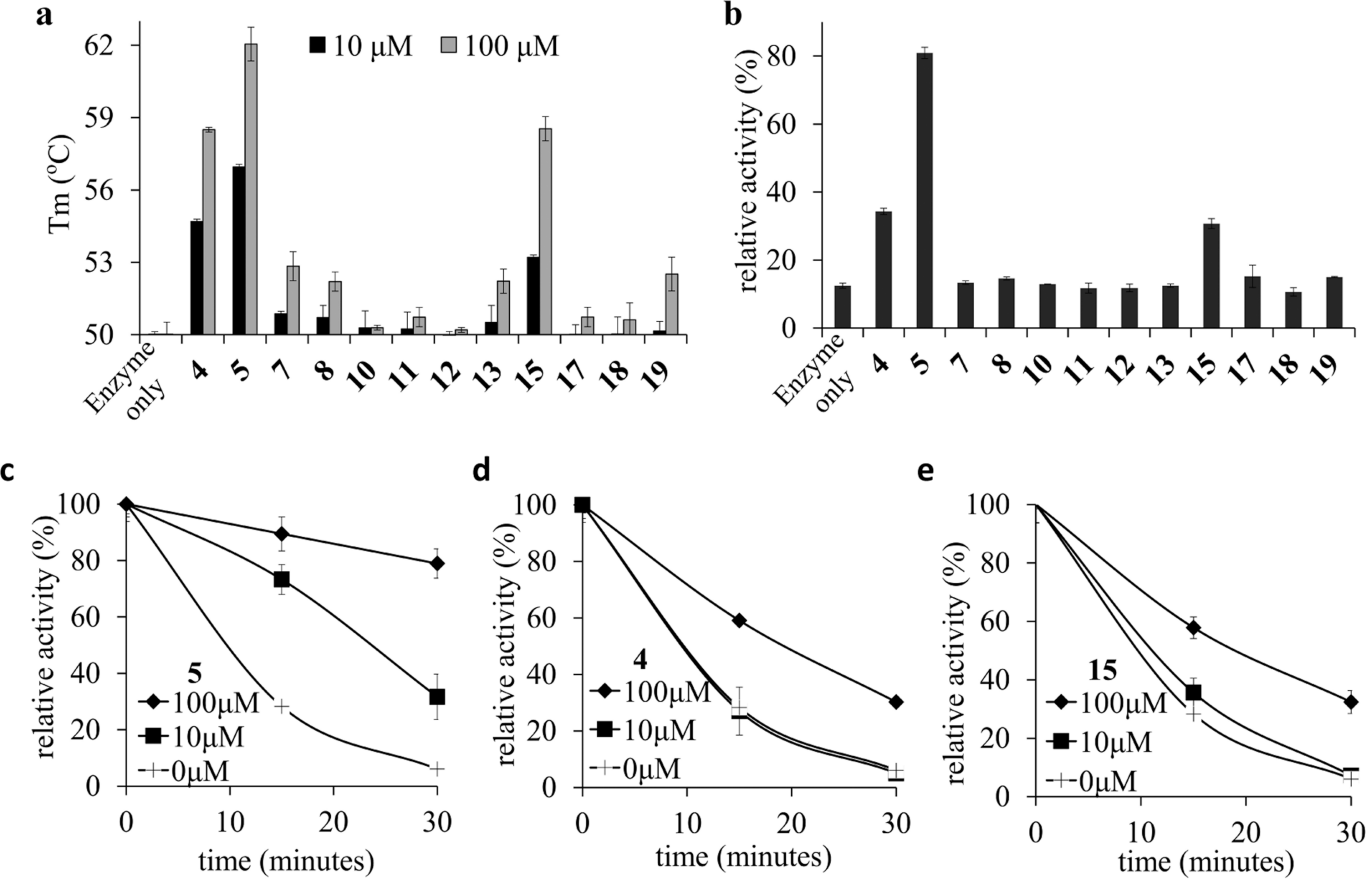
α-Gal A-stabilizing activity of iminosugars. (a) Unfolding *T*_m_ values of α-Gal A incubated with or without iminosugars. (b) Residual activity of α-Gal A incubated with or without 100 μM of iminosugars at 37 °C for 30 min. (c) Dose- and time-dependent protecting effects of **5** on α-Gal A. (d) Protecting effect of **4** on α-Gal A. (e) Protecting effects of **15** on α-Gal A.

To determine the rh-α-Gal A-stabilizing effects of these iminosugars, we conducted a fluorescence-based thermal shift assay to measure its melting temperature (*T*_m_).^30,31^ A solution of rh-α-Gal A was individually incubated with 10 and 100 μM iminosugars from 40 to 70 °C with fluorescent dye in pH 7.0 phosphate buffer (Figures 2a and S1). We found that 100 μM of **4** and **5** increased the *T*_m_ value of α-Gal A by 8.5 ± 0.1 and 12.0 ± 0.7 °C, respectively, which was much higher than that of other iminosugars, including C-2-deprived, C-2-extended, and C-2 *N*-alkylated derivatives. These results indicate that the C-2 hydroxymethyl and aminomethyl moieties are crucial for the observed protecting effects. Notably, iminosugar **15**, bearing an additional hydroxymethyl group, demonstrated protecting effects comparable to those of **4**, improving the *T*_m_ value by 8.5 ± 0.5 °C at 100 μM, indicating that modifications with a small functional group on the C-2 methylene do not interfere with the enzyme-stabilizing activity.

Next, the ability of the iminosugars to protect rh-α-Gal A from inactivation was evaluated by determining the residual activity of the enzyme in the Dulbecco’s Modified Eagle Medium (DMEM) at 37 °C for 30 min after its incubation with or without iminosugars (Figure 2b).^29^ Among the iminosugars tested, only **4**, **5**, and **15** exhibited significant protecting effects on the enzyme at 100 μM. Notably, the dibasic iminosugar **5** maintained the enzyme activity up to 80% of the initial value after incubation (Figure 2c), while C-2 hydroxymethyl iminosugars **4** and **15** preserved only ∼35% of the enzyme activity (Figure 2d,e), consistent with the thermal shift assay results. Taken together, these results indicate the significance of the specific placement of heteroatoms, particularly nitrogen, on the C-2 methylene in determining the protecting effects of five-membered iminosugars on α-Gal A. This critical positioning likely facilitates the interactions with adjacent residues within the active site of α-Gal A. In contrast, compounds possessing heteroatoms at different positions at the C-2 side chain, such as compounds **10**, **11**, **12**, or **13**, fail to engage with nearby residues, likely due to either considerable distances or unfavorable angles.

### Structural Basis for Binding of Pyrrolidine-Based Iminosugars to α-Gal A

**Figure 3:**
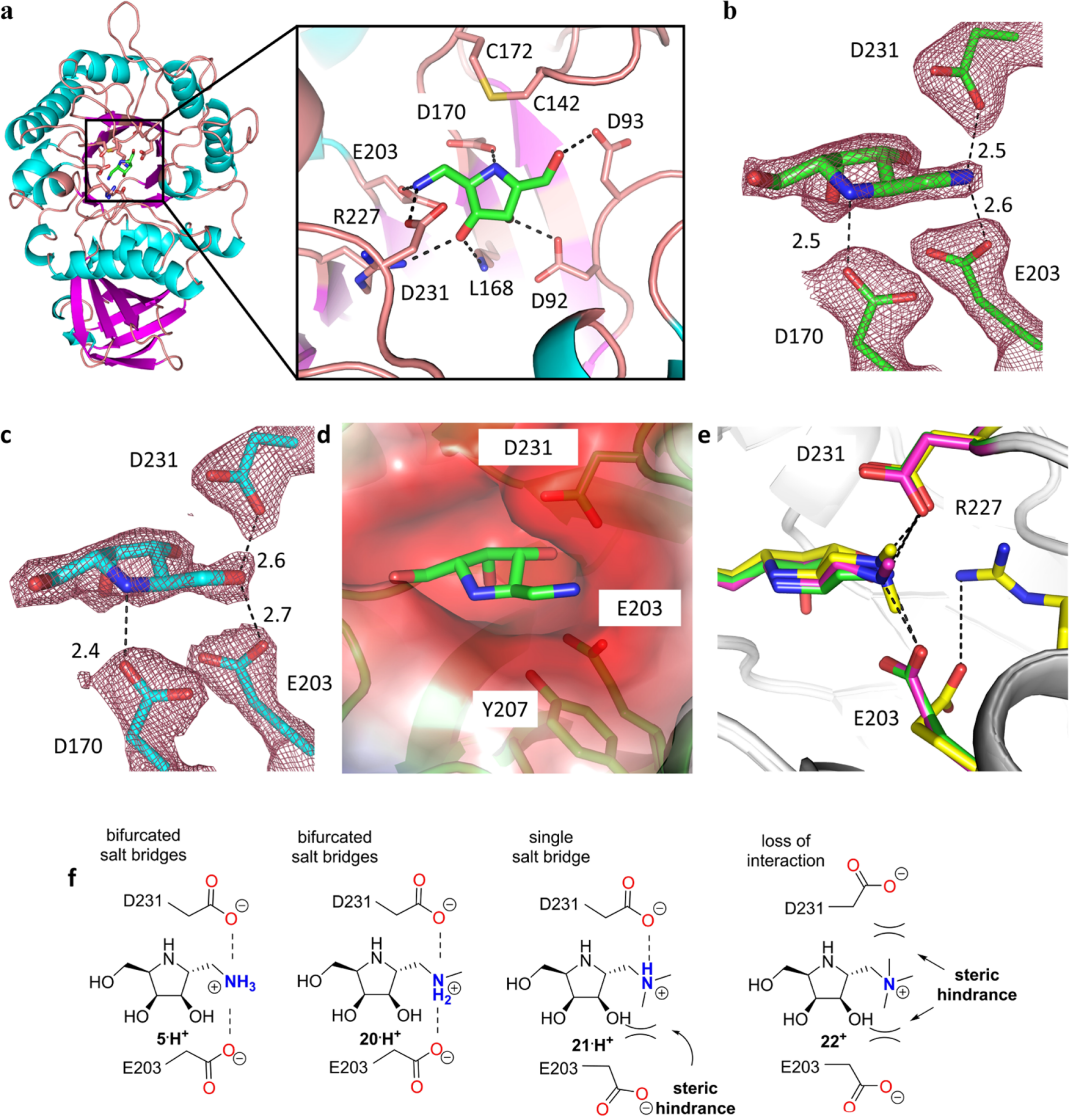
Crystal structures of α-Gal A bound to the iminosugars. (a) Overall structure of α-Gal A bound to compound **5** at pH 7.2. A close-up view of bound **5** is also shown. (b) 2*F*_o_ – *F*_c_ electron density around the bound compound **5** at pH 7.2. (c) 2*F*_o_ – *F*_c_ electron density around the bound compound **4** at pH 7.2. (d) Surface representation of the iminosugar-binding pocket with bound compound **5** depicted in a green stick model. The surface charge potentials are shown with blue and red colors representing the positive and negative charge, respectively. (e) Superimposition of the structures of α-Gal A bound to compounds **5** (green), **20** (pink), and **21** (yellow). (f) Proposed interactions between the *exo*-N of compounds **5**·**H**^**+**^, **20**·**H**^**+**^, **21**·**H**^**+**^, and **22**^**+**^, and the carboxyl residues of α-Gal A.

**Scheme 2:**
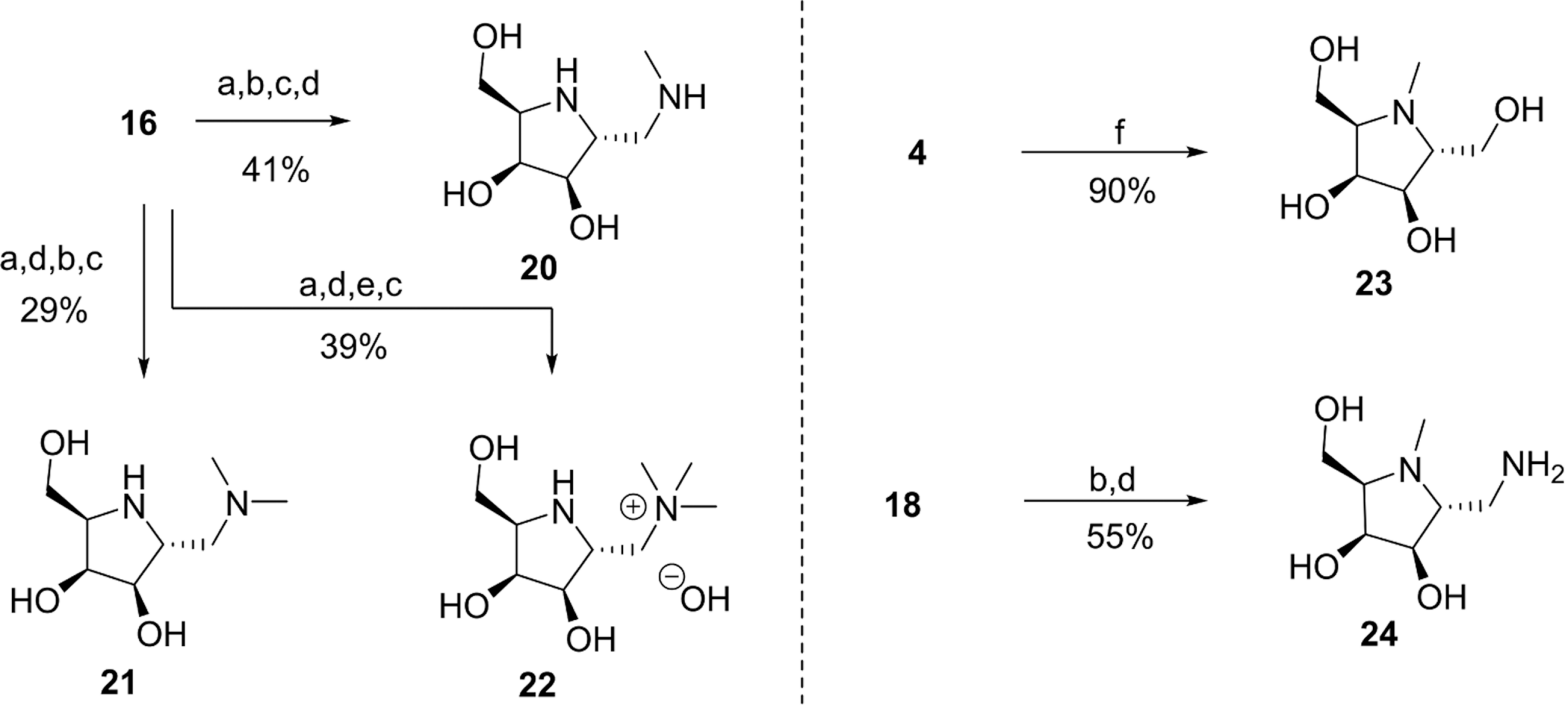
Synthesis of *N*-methylated Pyrrolidines Reagents and conditions: (a) NaBH_3_CN, NH_2_Bzh, AcOH, MeOH, rt, 12 h. (b) NaBH_3_CN, CH_2_O, AcOH, MeOH, rt, 12 h. (c) 6 N HCl, MeOH, rt, 12 h; then DOWEX (OH^–^). (d) Pd(OH)_2_/C, H_2_, MeOH, rt, 12 h. (e) MeI, MeOH, rt, 12 h. (f) H_2_, Pd(OH)_2_/C, CH_2_O, MeOH, rt, 12 h.

To unravel the binding mechanism of iminosugars with α-Gal A, *N*-methylated derivatives **20**–**24** were synthesized (Scheme 2 and Figure S1), and we determined the crystal structures of α-Gal A in complex with compounds **4** and **5** at pH 7.2 and 4.5, respectively, and **8**, **20**, **21**, **23**, and **24** at pH 7.2 at 1.97 to 2.61 Å resolution (Table S1).^2,32^ The structures revealed that these pyrrolidines bind to the catalytic site nearly in the center of the TIM-barrel domain of the enzyme. The bindings are primarily stabilized not only by hydrogen bonds (H-bonds) between the hydroxyl groups of the compounds and the residues D92, D93, L168, D170, E203, R227, and D231 of α-Gal A but also by a salt bridge between the endocyclic amino group (*endo*-N) of the monobasic compounds (p*K*_a_ ∼ 7.0) and D170 of the enzyme at pH 7.2 (Figures 3a and S2a).^33^ In contrast, the titration curves revealed that the endo-N moiety in compound **5** exhibits reduced basicity (p*K*_a_ = 5.5) compared to that of **4** (p*K*_a_ = 7.0) due to electrostatic repulsion between the vicinal ammonium cations (Figures S2b and S3),^34^ which implies the formation of an H-bond between *endo*-N of **5** and D170 at pH 7.0 but a salt bridge at pH 4.5. Surprisingly, in the compound **5**-bound structure of α-Gal A, the positively charged exocyclic amino group (*exo*-N, p*K*_a_ = 9.1) of **5** forms bifurcated salt bridges (BSBs) with residues E203 and D231 of the enzyme at pH 7.2 (Figures 3b, S2b, and S3), where the carboxylic side chains are essentially deprotonated and negatively charged. By contrast, the corresponding C-2 hydroxyl group of **4** forms H-bonds alone to E203 and D231 at pH 7.2 (Figure 3c). This finding most likely reflects the stronger binding affinity and better protecting effects of **5** toward α-Gal A at pH 7.2 compared to those of **4**. Interestingly, in the compound **8**-bound structure of α-Gal A, the position corresponding to the *exo*-N of **5** or C-2 hydroxyl group of **4** is occupied by a solvent molecule that bridges the gap between E203 and D231 (Figure S4a–c), indicating the crucial role of the C-2 aminomethyl and hydroxymethyl moieties of the compounds in H-bonding to the enzyme.

In addition, the *exo*-N of **5** (or the C-2 hydroxyl group of **4**) is docked into an extended but narrow pocket made by E203, Y207, and D231 of the enzyme (Figure 3d), presumably reflecting the reduced protecting effects of compounds **18** and **19**, which possess bulky substituents on the *exo*-N. To examine whether this pocket can accommodate small functional groups on the *exo*-N of **5**, iminosugars **20**, **21**, and **22** were synthesized (Scheme 2). Interestingly, the protecting effect of **20** (Δ*T*_m_ = 11.0 ± 0.5 °C) remained nearly unchanged compared to that of **5** (Δ*T*_m_ = 12.0 ± 0.7 °C), while the protecting effect of **21** was reduced (Δ*T*_m_ = 5.3 ± 0.5 °C) and **22** nearly completely absent (Figure S5). The solved structures of α-Gal A bound to **20** and **21** indicate that single methylation on *exo*-N had little effect on the salt bridges to E203/D231 of α-Gal A (Figure S4d), but double methylations on the *exo*-N led to a loss of the salt bridge to E203 due to a large steric hindrance between the second methyl group and E203 (Figures 3e and S4e). Based on this structural observation, it is reasonable to assume that triple methylations on the *exo*-N resulted in the loss of salt bridges to both E203/D231 (Figure 3f). These findings suggest that long but not bulky groups added to the *exo*-N of dibasic iminosugars may have a chance to further improve their protecting effects.

Our structures also indicate that the *endo*-N of the compounds forms a salt bridge (or H-bond) to the residue D170 of α-Gal A and also van der Waals contacts with the disulfide linkage C142–C172 (Figure S4f). To probe whether an extra group added to the *endo*-N can improve the protecting effects of the compounds, iminosugars **23** and **24** were synthesized (Scheme 2). However, their protecting effects on α-Gal A were reduced (Δ*T*_m_ ∼ 5 °C) as shown in Figure S5. The solved structures of α-Gal A bound to **23** and **24** indicate that the extra methyl group on the *endo*-N has interacted little with the enzyme (Figure S4g,h). The methyl groups led to a slight rotation of **23** and **24** compared to **4** and **5**, respectively, probably due to the steric hindrance on the salt bridge to D170 (Figure S4i,j), resulting in their reduced protecting effects. Furthermore, partial deprotonation of *endo*-N at pH 7.2 leading to the partial loss of both salt bridge and H-bond to D170 is also possible (Figure S4k).

Given these structural findings, it is conceivable that the pH-dependent protonation states on the amino groups of iminosugars and ionizable residues in the catalytic site of α-Gal A regulate the binding status of the compounds,^35^ i.e., the stronger binding of **5** to α-Gal A at pH 7.2 is due to the formation of the BSBs, which are missing at pH 4.5 when the binding is weaker and only H-bonds are formed.

### Thermodynamic and Kinetic Studies for Interactions between Pyrrolidine-Based Iminosugars and α-Gal A

**Figure 4:**
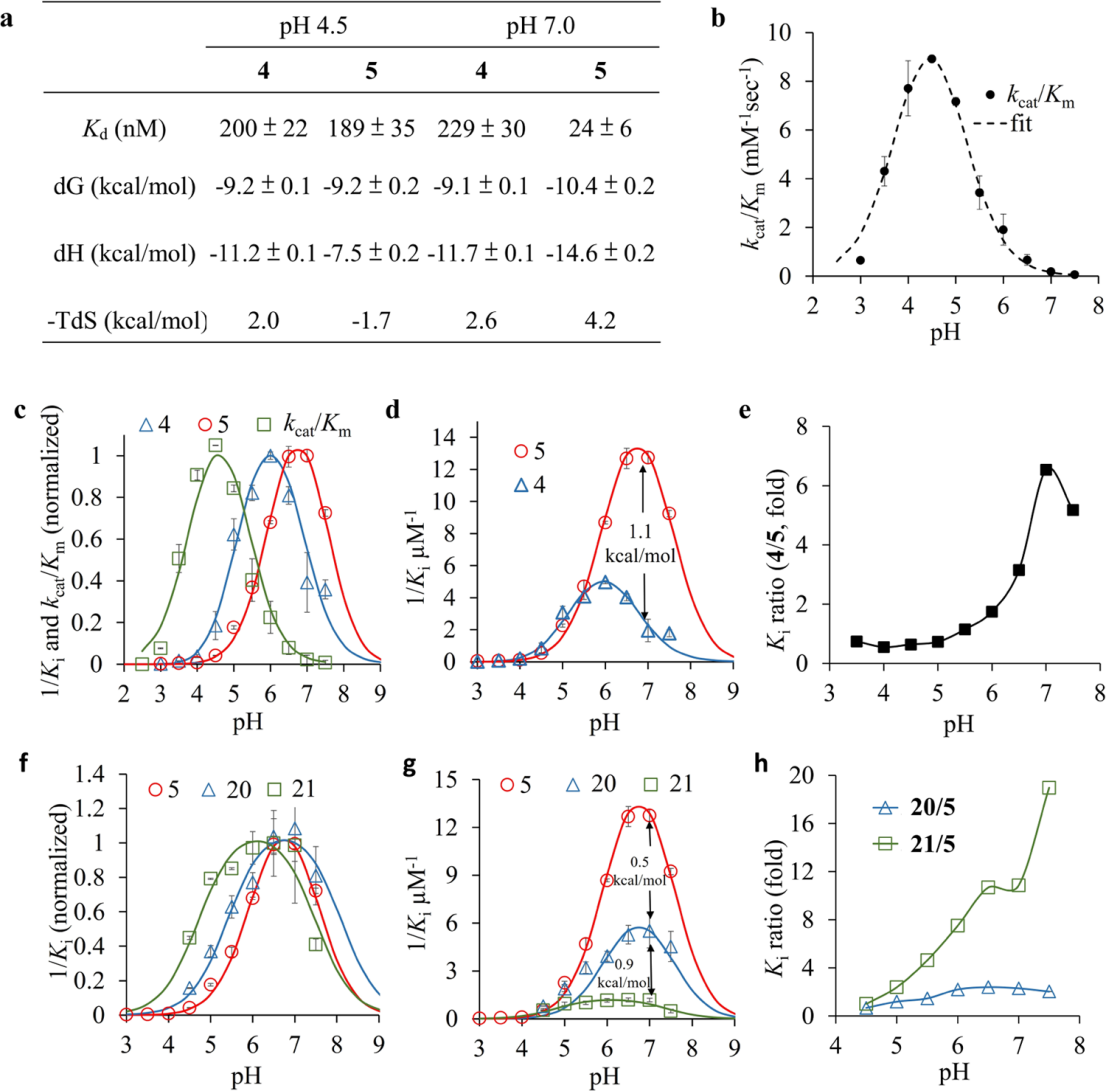
Thermodynamic and kinetic analyses of the interactions between the iminosugars and α-Gal A. (a) Thermodynamic analysis on the binding of **4** and **5** to α-Gal A at pH 4.5 and 7.0 using ITC. (b) pH profile of *k*_cat_/*K*_m_ values of α-Gal A. (c) Normalized pH profiles of 1/*K_i_* values for **4** and **5** against α-Gal A. The *k*_cat_/*K*_m_ profile of α-Gal A is also shown for comparison. (d) Comparison of pH profiles of 1/*K_i_* values for **4** and **5**. (e) pH profile of the *K_i_* ratio for **4:5**. (f) Normalized pH profiles of 1/*K_i_* values for **5**, **20**, and **21**. (g) Comparison of pH profiles of 1/*K_i_* values for **5**, **20**, and **21**. (h) Comparison of pH profiles of *K_i_* ratios for **20:5** and **21:5**.

To confirm that the pH-selectivity of **5** was due to the BSBs with residues E203 and D231 of α-Gal A, the thermodynamic and kinetic parameters for interactions between the iminosugars and α-Gal A at different pH conditions were determined. As analyzed by using isothermal titration calorimetry (ITC), we observed similar binding energies at pH 4.5 for **4** (−9.2 ± 0.1 kcal/mol) and **5** (−9.2 ± 0.1 kcal/mol), but at pH 7.0, the binding energy of **5** (−10.4 ± 0.2 kcal/mol) was significantly lower than that of **4** (−9.1 ± 0.1 kcal/mol), owing to a substantially enhanced enthalpy (Δ*H*_pH 7.4_ – Δ*H*_pH 4.6_ = −7.1 kcal/mol) as shown in Figures 4a and S6. This finding supports that the formation of BSBs is responsible for the stronger binding of **5** than **4** at pH 7.0. In addition, we also noticed that the binding of compound **5** compared to compound **4** with α-Gal A at pH 4.5 exhibits a substantial reduction in binding enthalpy (Δ*H*_5_ – Δ*H*_4_ = +3.7 kcal/mol) and a simultaneous enhancement in binding entropy (Figure 4a). This reduced binding enthalpy may reflect the disrupted H-bonds due to the intramolecular electrostatic repulsion between two charged ammonium groups in compound **5**, while the enhanced binding entropy could be a result of the enthalpy–entropy compensation due to the distorted binding conformation.^36^ To gain further insight into the protonation states of the catalytic residues in α-Gal A, the specificity constants (*k*_cat_/*K*_m_) of the enzyme at different pH values were determined (Figure 4b). We found that α-Gal A had a bell-shaped catalytic profile with maximal activity at pH ∼ 4.5 with the acidic and basic limbs of p*K*_a_ values of 3.8 ± 0.1 and 5.1 ± 0.1, which reflect essentially the titration of the catalytic nucleophile D170 and acid/base D231, respectively.^37^ Specifically, both of the catalytic residues are fully deprotonated at pH 7.0, where, when binding to **5**, the positively charged *exo*-N of the compound forms a strong electrostatic interaction with D231 of the enzyme.

To better understand how the ionizable groups in the iminosugars and α-Gal A active site contribute to the binding at different pH values, we determined the pH-dependent inhibition of the enzyme by the iminosugars.^35,38,39^ The pH profile of 1/*K_i_* for monobasic iminosugar **4** also showed a bell-shaped curve with an optimal pH at 6.0, and the acidic and basic limb p*K*_a_ values of 5.1 ± 0.1 and 6.8 ± 0.2, respectively (Figure 4c). The acidic limb likely reflects the deprotonation of the acid/base, while the basic limb reflects the deprotonation of the *endo*-N of the compound. It is thus suggestive that the binding of monobasic iminosugars to α-Gal A prefers the *endo*-N of the compounds being protonated and the catalytic residues of the enzyme deprotonated (Figure S7a).^40,41^ By contrast, the dibasic iminosugar **5** showed a strikingly alkaline-shifted curve with the acidic and basic limb p*K*_a_s of 5.9 ± 0.1 and 7.7 ± 0.2, respectively, and an optimal pH at ∼6.8. Presumably, in addition to the *endo*-N and the catalytic residues, the ionizations of the *exo*-N and residue E203 also modulate the pH profile of **5**, resulting in a pH-selectivity different from **4**.

As estimated from the inhibition constants of **4** and **5** against α-Gal A at pH 7.0, we found that the binding energy of **5** was ∼1.1 kcal/mol lower than that of **4** (Figure 4d), consistent with the results obtained from ITC. A plot of 1/*K_i_* ratios of **5:4** indicated that both compounds had a similar binding affinity against α-Gal A at low pH (pH 3.0–5.0), while the ratio increased drastically between pH 5.0 and 7.0 (Figure 4e). This result suggests that E203 was likely deprotonated between pH 5.0 and 7.0, leading to the considerably increased level of binding of **5** to α-Gal A through the formation of a salt bridge with the *exo*-N of the compound. The deprotonation of E203 likely contributes to the alkaline-shifted acidic limb of **5**, while the alkaline-shifted basic limb of **5** may reflect the deprotonation of the *exo*-N in **5** compared to **4**.

To further confirm that the interaction between the *exo*-N of **5** and E203 of α-Gal A contributes to the alkaline-shifted acidic limb of this compound, the pH-dependent inhibition profiles of compounds **20** and **21** were determined (Figure 4f). We found that the p*K*_a_ values in the acidic and basic limbs of **20** were 5.4 ± 0.2 and 8.1 ± 0.3, respectively, and both are close to that of **5**. By contrast, the p*K*_a_ value in the acidic limb of **21** was decreased significantly (4.7 ± 0.2). This data indicates that a loss of the interaction between the *exo*-N and E203 results in an acidic shift of the acid limb of **21** compared to that of **5** and **20**. Additionally, by estimating the inhibition constants of **5**, **20**, and **21** against α-Gal A at pH 7.0, we found that the binding energy of **5** was ∼1.4 kcal/mol lower than that of **21** (Figure 4g). This probably reflects a loss of the salt bridge between **21** and E203 of α-Gal A, owing to steric hindrance (Figure 3f). The plot of 1/*K*_i_ ratios for **5:21** showed a substantial increase from 1 to 18 between pH 4.5 and 7.5, whereas the ratios for **5:20** were in the range of 0.5–2.5 (Figure 4h). These results also support the importance of the salt bridge between *exo*-N of dibasic iminosugars and E203 of α-Gal A in their pH-selective binding.

Taken together, the binding of dibasic iminosugars **5** and **20** to α-Gal A is more favorable at physiological pH conditions due to the formation of the BSBs between the positively charged *exo*-N of the compounds and the negatively charged residues E203/D231 of α-Gal A (Figure S7b), whereas in the acidic environment of lysosomes, where E203/D231 of the enzyme and the *endo*-N of **5** should be protonated, the binding becomes weaker due to loss of BSBs and the intramolecular electrostatic repulsion between the *exo*-N and *endo*-N of the compounds.

### Protecting Effects of Iminosugars **4**, **5**, **20**, **21**, **22**, **and 24** in FD Cells

**Figure 5:**
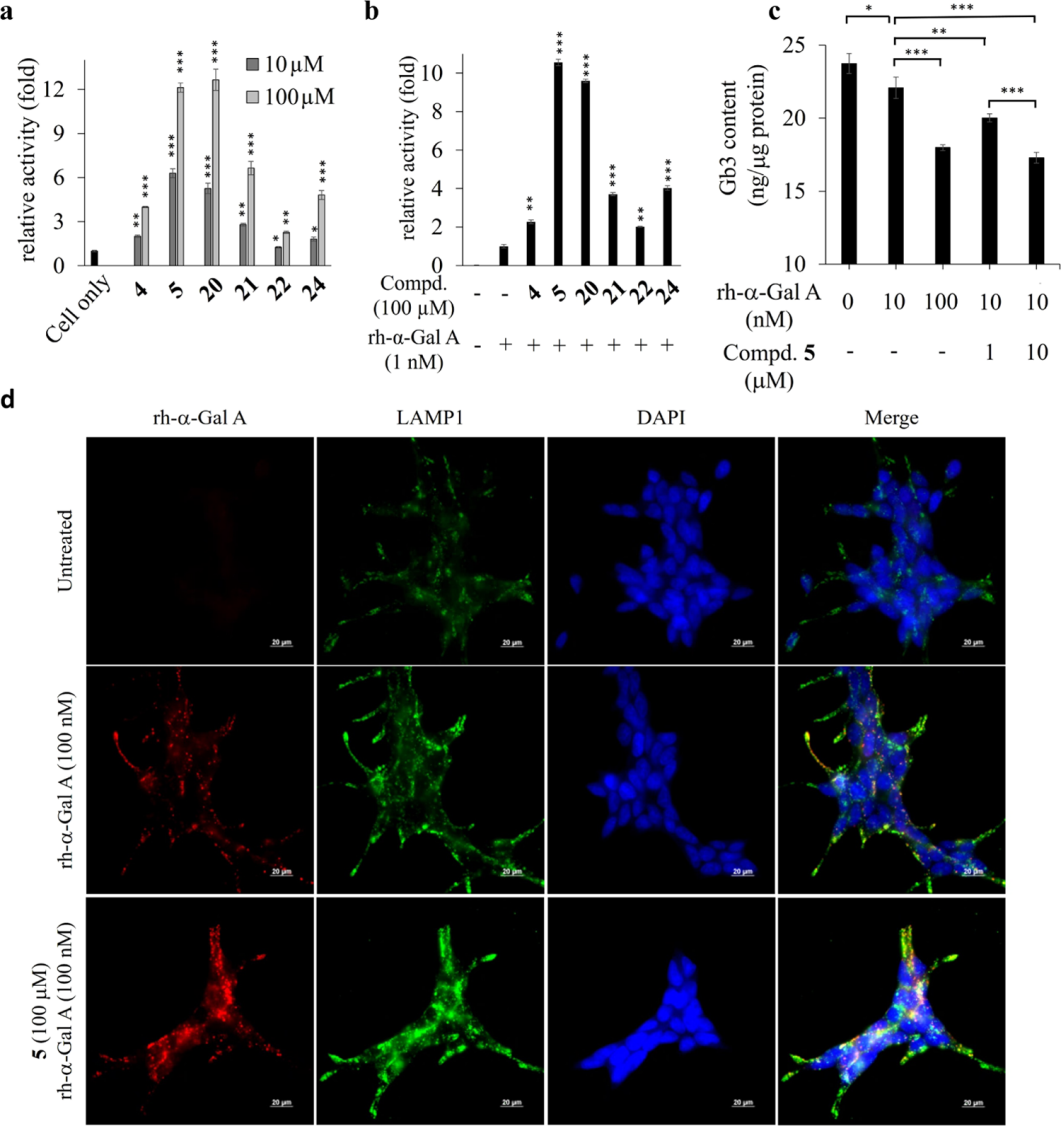
Protecting effects of iminosugars in FD cells. (a) Chaperoning activity of iminosugars (10, 100 μM) in cultured N215S lymphocytes. (b) α-Gal A activity in lysates of cultured W162X fibroblasts treated iminosugars (100 μM) in combination with rh-α-Gal A (1 nM). (c) Gb3 levels in W162X fibroblasts were measured 5 days after treatment by LC-MS/MS. (d) Immunofluorescence in *GLA* KO HEK293. Staining was performed for rh-α-Gal A (red) and LAMP1 (green). Scale bar: 20 μm. The data bars shown are the mean ± SDM of 3 wells tested in parallel from one representative of three independent experiments. *p < 0.05, ** *p* < 0.01, *** *p* < 0.005 (*t*-test).

To confirm that the BSBs of the iminosugars are an important contributor to their protecting effects, FD cells were used to evaluate their chaperone activity and ERT-improving efficacy. The capacity of iminosugars **4**, **5**, **20**, **21**, **22**, and **24** to improve α-Gal A activity was assessed in FD lymphocytes with N215S missense pathogenic variant (GM04391, Coriell) as shown in Figure 5a. Dibasic iminosugars **5** and **20** that possess the BSBs with D231 and E203 showed a remarkable 12.1- and 12.7-fold enhancement at 100 μM, respectively, while monobasic and other *N*-methylated iminosugars showed mild to moderate enhancement effects from 2.7- to 6.2-fold, compared with untreated cells. To further test the protecting effects of these iminosugars, FD fibroblasts lacking α-Gal A activity due to the W162X nonsense variant (GM00107, Coriell) were cotreated with 1 nM rh-α-Gal A and 100 μM iminosugars. As shown in Figure 5b, all iminosugars had significant protecting effects when compared with those of rh-α-Gal A alone. It was noted that dibasic iminosugars **5** and **20** improved cellular α-Gal A activity as high as 10.6- and 9.6-fold than without protection. These results suggested that the C-2 aminomethyl moiety is critical for improving the protecting effect of pyrrolidine-based iminosugars on α-Gal A due to the formation of BSBs with D231/E203.

To investigate whether enhancement of intracellular α-Gal A activity improves Gb3 clearance, the intracellular Gb3 contents of the W162X FD fibroblasts were quantified by LC-MS/MS. The fibroblasts were cotreated with rh-α-Gal A and **5** for 24 h, and the intracellular Gb3 contents were analyzed at 96 h post-treatment. The Gb3 content was decreased by 15% when cotreated with 10 nM rh-α-Gal A and 1 μM **5** and significantly improved when compared with 10 nM rh-α-Gal A only. Importantly, the improvement of Gb3 clearance by 10 nM rh-α-Gal A plus 10 μM **5** was more dramatic (25% decrease) and comparable to that with 100 nM rh-α-Gal A alone (Figure 5c).

The immunofluorescence assay showed the localization of rh-α-Gal A in *GLA* KO HEK293. The *GLA* KO HEK293 cell line was produced through CRISPR/Cas9 technology, and the endogenous *GLA* gene locus was completely disrupted (Figure 5d). After treatment with 100 nM rh-α-Gal A, the recombinant protein had been taken up and colocalized with the lysosomal marker, LAMP1. The α-Gal A fluorescent signals were significantly enhanced when cotreated with 100 μM of **5**, demonstrating that improved cellular activity might have resulted from the increased intracellular protein level. Taken together, these results provide a new insight that dibasic amino-iminosugars are better PCs for improving intracellular α-Gal A protein level and activity and also Gb3 clearance in FD cells when compared with monobasic ones.

### Assessment of the *In Vivo* Protecting Effect of Dibasic Iminosugar **5** on rh-α-Gal A in a *Gla* KO Mice Model

**Figure 6:**
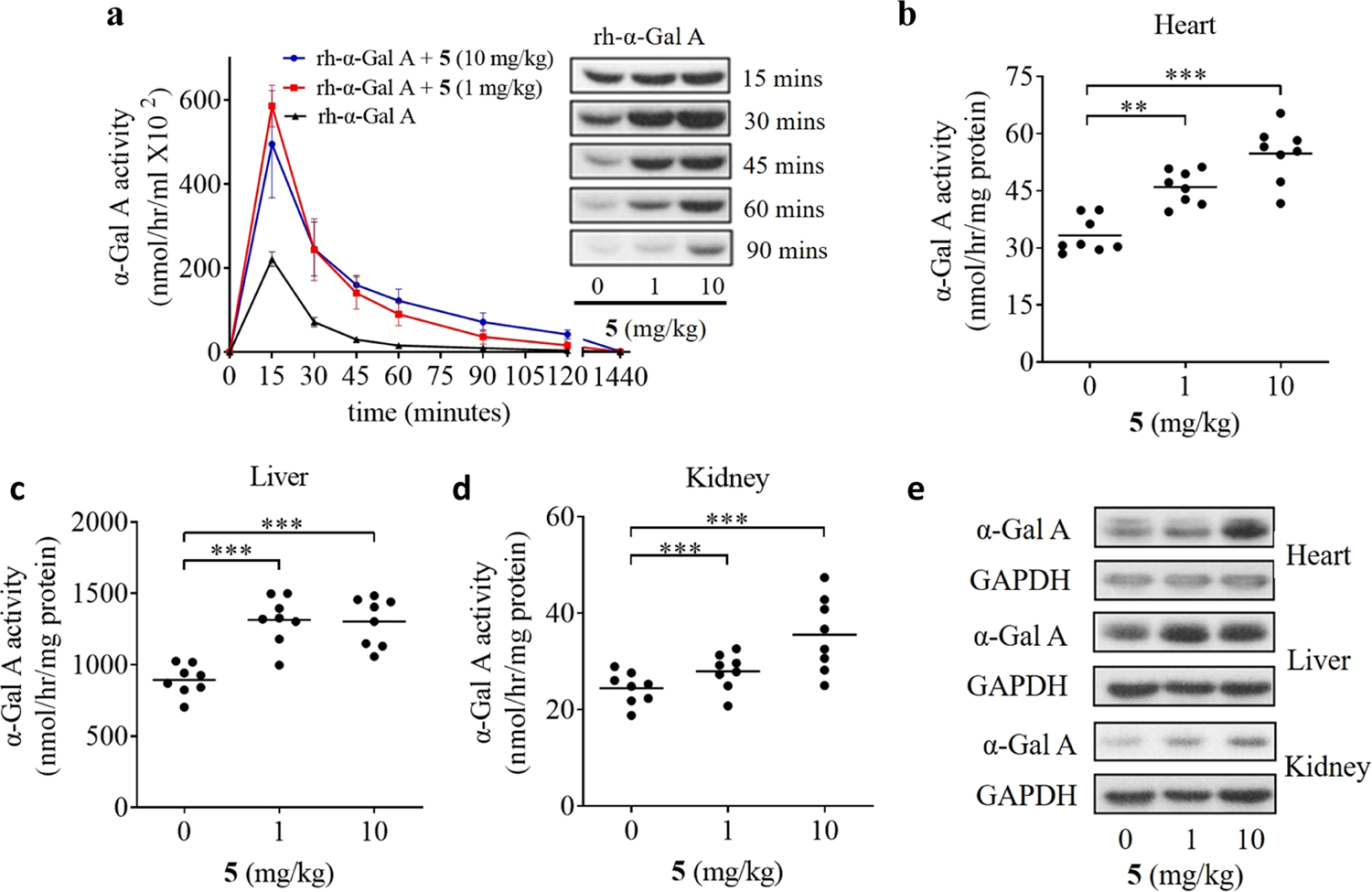
Enhancement of ERT by **5** in *Gla* KO mice. (a) rh-α-Gal A (3 mg/kg) mixed with or without **5** (1 and 10 mg/kg) was administered to *Gla* KO mice (*n* = 5). Plasma was collected at different times to test the activity of α-Gal A, and a Western blot was used to determine the protein of rh-α-Gal A in plasma. (b–e) rh-α-Gal A (3 mg/kg) and **5** (0, 1, and 10 mg/kg) were coadministered to mice and sacrificed on day 1. The heart, liver, and kidney were tested for their enzyme activity and protein level (*n* = 8–11). *p < 0.05, ** *p* < 0.01, *** *p* < 0.005 (one-way ANOVA with Tukey’s posthoc).

The protecting effect of dibasic iminosugar **5** was evaluated in *Gla* KO mice by monitoring the half-life of rh-α-Gal A in the plasma. Different concentrations of iminosugar **5** (0, 1, and 10 mg/kg) were incubated with 3 mg/kg rh-α-Gal A at room temperature for 10 min. After an intravenous injection to *Gla* KO mice (8–12 weeks old), the plasma α-Gal A activity was assayed at different times (15, 30, 45, 60, 90, 120 min, and 24 h) as shown in Figure 6a. The highest enzymatic activity was shown at 15 min post intravenous infusion, when **5** enhanced the activity by 2.6-fold compared to rh-α-Gal A alone. In addition, the enzyme activity was significantly prolonged at 2 h post-treatment with 10 mg/kg of **5** compared to the control group (42.4 ± 10.4 × 10^2^ versus 4.6 ± 2.5 × 10^2^ nmol/h/mL, *p* < 0.001). These data demonstrated that rh-α-Gal A alone has a half-life of ∼8.6 min in plasma and was improved to ∼11.9 and ∼14.8 min with 1 or 10 mg/kg of **5**, respectively. Concordantly, the enhanced protein levels also supported the beneficial effects of cotreatment of **5** in stabilizing circulated rh-α-Gal A. The data indicated that iminosugar **5** protected circulating rh-α-Gal A *in vivo* and prolonged the half-life.

To test whether the protecting effects of iminosugar **5** facilitated tissue uptake of *Gla* KO mice, we harvested mouse tissues after injection and assayed their α-Gal A activities. The enzyme activity from the mouse heart was significantly enhanced when cotreated with 1 and 10 mg/kg of **5** (46.0 and 54.8 nmol/h/mg) for 24 h, compared with the treatment of rh-α-Gal A alone (33.3 nmol/h/mg) as shown in Figure 6b. A similar improvement was observed in the mouse liver and kidney (Figure 6c,d). These data demonstrated that both 1 and 10 mg/kg **5** significantly improved enzyme levels in mouse tissues. It also suggested that **5** might protect circulated rh-α-Gal A and enhance tissue uptake *in vivo*. The long-term effect of **5** toward rh-α-Gal A was also observed in the organs after 7 days post-treating with rh-α-Gal A (3 mg/kg) and 0, 1, and 10 mg/kg of **5** (Figure S8a–c). Similarly, the rh-α-Gal A activities in mouse hearts were increased by 2.1- and 2.7-fold by cotreating with 1 and 10 mg/kg of **5**, when compared with treating rh-α-Gal A alone. Also, the residual activity was improved by around 2.0-fold in the liver and kidney in the cotreatment group. In addition, greater rh-α-Gal A protein levels following cotreatment of **5** for both 1 and 7 days were confirmed by Western blotting (Figures 6e and S8d). These data showed that 1 mg/kg of **5** was effective and able to prolong the activity of rh-α-Gal A in mice for 7 days post-infusion. Taken together, these results support the enhancement by iminosugar **5** of tissue α-Gal A activity post-infusion through the prolonged half-life of circulating rh-α-Gal A in plasma, facilitating tissue uptake *in vivo.*

## Conclusions

In summary, we have made a significant discovery regarding the modulation of pH-selective binding of iminosugars to α-Gal A. Our study of the α-Gal A-stabilizing activity of monobasic and dibasic iminosugars revealed that the C-2 aminomethyl moiety plays a crucial role in preventing enzyme denaturation and inactivation. Notably, the superior pH-selective binding and protecting effects of dibasic iminosugars, in contrast to monobasic ones, are attributed to the formation of BSBs between the *exo*-N of the dibasic iminosugars and the carboxyl residues E203/D231 of α-Gal A at pH 7.0 and, additionally, to the intramolecular electrostatic repulsion between the *exo*-N and *endo*-N of the compounds at pH 4.5. Of all of the iminosugars examined, dibasic iminosugar **5** exhibited optimal binding to α-Gal A at pH ∼ 6.8, where positively charged *exo*-N and negatively charged E203/D231 interact, driven by a substantially enhanced enthalpy. We also found that the BSBs of iminosugars to the enzyme are crucial for their chaperoning activity and ERT-improving efficacy in FD cells. Furthermore, dibasic iminosugar **5** significantly enhanced tissue α-Gal A activity, highlighting its protecting effects on the enzyme *in vivo*. Therefore, the installation of an additional amino group onto iminosugars is proposed as a useful strategy to modulate the binding of iminosugars to target enzymes for the design of PCs or neutral-bound inhibitors.

## Supporting Information Available

Additional experimental details, materials, methods, and characterization data, including NMR spectra (PDF)

## Abbreviations

PC: pharmacological chaperone
α-Gal A: human α-galactosidase A
ERT: enzyme replacement therapy
FD: Fabry disease
Gb3: globotriaosylceramide
LSD: lysosomal storage disease
ER: endoplasmic reticulum
IV: intravenous
PCT: pharmacological chaperone therapy
DNJ: 1-deoxynojirimycin
DGJ: 1-deoxygalactonojirimycin
Tm: melting temperature
DMEM: Dulbecco’s Modified Eagle Medium
H-bond: hydrogen bond
*endo*-N: endocyclic amino group
*exo*-N: exocyclic amino group
BSB: bifurcated salt bridge
KO: knockout
