# Supplementary material for: Mechanistic Insights into Dibasic Iminosugars as pH-Selective Pharmacological Chaperones to Stabilize Human α-Galactosidase
Source: JACS Au. 2024 Feb 23;4(3):908–18. doi: 10.1021/jacsau.3c00684 (PMC10976572; doi:10.1021/jacsau.3c00684)
Supplement: Supplementary file 1 — au3c00684_si_001.pdf [file au3c00684_si_001.pdf]

Supporting information

Mechanistic insights into dibasic iminosugars as pH-selective pharmacological chaperones to stabilize human α-galactosidase

Huang-Yi Li[1,2], Hung-Yi Lin[1], Sheng-Kai Chang[3], Yu-Ting Chiu[1], Chung-Chien Hou[1], Tzu-Ping Ko[4], Kai-Fa Huang[4], Dau-Ming Niu[3,5*], Wei-Chieh Cheng[1,6,7,8*]

[1] Genomics Research Center, Academia Sinica, 128, Section 2, Academia Road, Nankang, Taipei 115201, Taiwan.
[2] Institute of Biochemistry and Molecular Biology, National Yang Ming Chiao Tung University, 155, Section 2, Linong Street, Taipei 112304, Taiwan.
[3] Department of Pediatrics, Taipei Veterans General Hospital, 201, Section 2, Shipai Road, Beitou, Taipei 112201, Taiwan.
[4] Institute of Biological Chemistry, Academia Sinica, 128, Section 2, Academia Road, Nankang, Taipei 11529, Taiwan.
[5] Institute of Clinical Medicine, School of Medicine, National Yang Ming Chiao Tung University, 155, Section 2, Linong Street, Taipei 112304, Taiwan.
[6] Department of Chemistry, National Cheng Kung University, 1, University Road, East, Tainan 701401, Taiwan.
[7] Department of Chemistry, National University of Kaohsiung, 700, University Road, Nanzih, Kaohsiung 811726, Taiwan.
[8] Department of Chemistry, National Chiayi University, 300, Syuefu Road, Chiayi 600355, Taiwan.

*corresponding authors
Dau-Ming Niu dmniu.submission@gmail.com,
Wei-Chieh Cheng wcheng@gate.sinica.edu.tw

Table of contents

1. Supporting figures and tables S3
2. Biochemical and biological methods S17
3. Chemical Synthesis S21
4. NMR Spectra S36
5. References S66

1. Supporting figures and tables

(a)

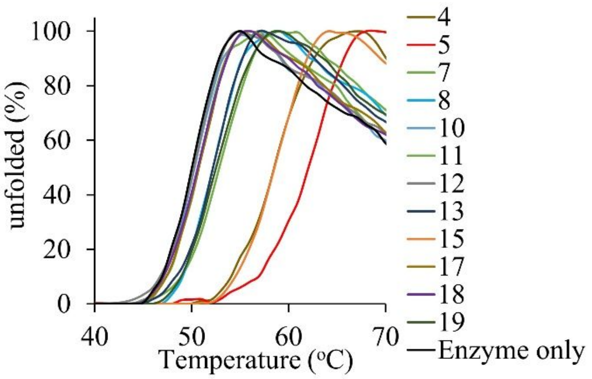

(b)

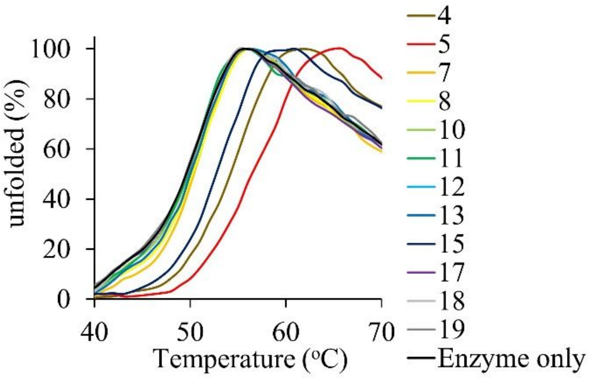

Fig. S1 Unfolding curves of α-Gal A. α-Gal A was incubated with or without (a) 100 or (b) 10 μM iminosugars monitored by changes in the fluorescence of SYPRO Orange as a function of temperature. The curves are shown for triplicate experiments.

Table S1 X-ray Data collection and refinement statistics

**Data collection**			
Crystal	rh-α-Gal A-**4** pH 4.5	rh-α-Gal A-**4** pH 7.2	rh-α-Gal A-**5** pH 4.5
Space group	$P3_221$	$P3_221$	$P3_221$
Cell dimensions			
a, b, c (Å)	90.6, 90.6, 216.7	90.6, 90.6, 216.1	90.6, 90.6, 216.7
α, β, γ (°)	90.0, 90.0, 120.0	90.0, 90.0, 120.0	90.0, 90.0, 120.0
Resolution (Å)	30-1.97	30-2.32	30-2.61
Unique reflections[a]	72505 (7020)[b]	45211 (4380)	31820 (3102)
Redundancy	10.4 (7.1)	6.6 (3.5)	5.0 (5.1)
Completeness (%)	99.8 (98.3)	99.9 (99.5)	99.0 (98.5)
$I/\sigma(I)$	32.3 (2.1)	14.1 (2.1)	13.5 (2.0)
R_{merge} (%)	7.3 (71.9)	12.3 (59.4)	10.0 (70.1)
**Refinement (Å)**			
Resolution (Å)	29.7-1.98	29.7-2.32	28.2-2.61
Reflections (> 0 $\sigma(F)$)			
Working/test	65642/3443	41311/3677	30040/1510
R_{factor}/R_{free}[b]	0.156/0.191	0.168/0.217	0.174/0.209
RMS deviations			
Bond lengths (Å)/angles (°)	0.007/0.93	0.006/0.94	0.003/0.73
B-factor (Å^2)/no. of atoms			
Protein	30.0/6261	38.2/6261	49.2/6261
Glycan	73.2/170	74.9/156	81.7/148
Ligand	28.4/22	41.7/22	47.4/22
Solvent	41.2/678	47.2/570	52.9/445
Ramachandran plot (%)			
Favored	97.6	97.7	96.9
Allowed	2.1	2.0	3.1
Outliers	0.3	0.3	0
PDB ID code	8K7F	8K7G	8K7D

[a]Values in parentheses correspond to the highest resolution shell.

[b]Five percent of randomly selected data were set aside for calculating R_{free}.

**Data collection**			
Crystal	rh-α-Gal A-**5** pH 7.2	rh-α-Gal A-**8** pH 7.2	rh-α-Gal A-**20** pH 7.2
Space group	$P3_221$	$P3_221$	$P3_221$
Cell dimensions			
a, b, c (Å)	90.4, 90.4, 216.4	90.5, 90.5, 216.3	90.1, 90.1, 215.5
α, β, γ (°)	90.0, 90.0, 120.0	90.0, 90.0, 120.0	90.0, 90.0, 120.0
Resolution (Å)	30-2.20	30-2.28	30-2.12
Unique reflections[a]	52803 (5184)	47648 (4650)	57776 (5223)
Redundancy	11.0 (10.1)	5.9 (5.0)	10.3 (4.4)
Completeness (%)	100.0 (100.0)	100.0 (100.0)	99.0 (90.3)
$I/\sigma(I)$	17.4 (2.3)	13.1 (2.4)	18.2 (2.0)
R_{merge} (%)	13.4 (98.9)	15.9 (86.7)	11.8 (56.1)
**Refinement (Å)**			
Resolution (Å)	29.6-2.20	29.6-2.28	29.5-2.12
Reflections (> 0 σ(*F*))			
Working/test	47850/3656	45825/3751	54313/1989
R_{factor}/R_{free}[b]	0.161/0.192	0.173/0.210	0.161/0.195
RMS deviations			
Bond lengths (Å)/angles (°)	0.004/0.88	0.003/0.76	0.004/0.80
B-factor ($Å^2$)/no. of atoms			
Protein	38.0/6261	35.8/6267	30.7/6291
Glycan	67.9/134	73.3/148	71.9/184
Ligand	42.8/22	37.2/20	33.4/24
Solvent	44.9/567	44.1/556	44.6/778
Ramachandran plot (%)			
Favored	97.4	97.0	97.3
Allowed	2.3	2.7	2.4
Outliers	0.3	0.3	0.3
PDB ID code	8K7E	8K7H	8K7I

**Data collection**			
Crystal	rh-α-Gal A-**21** pH 7.2	rh-α-Gal A-**23** pH 7.2	rh-α-Gal A-**24** pH 7.2
Space group	$P3_221$	$P3_221$	$P3_221$
Cell dimensions			
a, b, c (Å)	90.7, 90.7, 216.3	90.4, 90.4, 216.2	90.1, 90.1, 215.9
α, β, γ (°)	90.0, 90.0, 120.0	90.0, 90.0, 120.0	90.0, 90.0, 120.0
Resolution (Å)	30-2.01	30-2.00	30-2.00
Unique reflections[a]	68290 (6025)	69920 (6866)	67902 (5624)
Redundancy	10.2 (5.0)	12.0 (10.1)	10.4 (5.0)
Completeness (%)	98.6 (88.6)	100.0 (100.0)	98.0 (83.0)
$I/\sigma(I)$	21.1 (2.1)	20.8 (2.1)	20.3 (2.7)
R_{merge} (%)	10.5 (65.7)	12.1 (94.2)	11.8 (42.0)
**Refinement (Å)**			
Resolution (Å)	29.7-2.01	29.6-2.00	29.5-2.00
Reflections (> 0 σ(*F*))			
Working/test	63564 /2005	67182/2003	64676/1976
R_{factor}/R_{free}[b]	0.158/0.191	0.154/0.186	0.158/0.186
RMS deviations			
Bond lengths (Å)/angles (°)	0.007/0.91	0.009/0.97	0.005/0.81
B-factor ($Å^2$)/no. of atoms			
Protein	30.5/6313	29.9/6296	29.0/6266
Glycan	75.4/198	68.9/156	70.0/184
Ligand	23.6/26	24.0/24	27.1/24
Solvent	43.5/766	42.7/807	43.8/801
Ramachandran plot (%)			
Favored	96.7	97.3	97.3
Allowed	3.1	2.4	2.4
Outliers	0.3	0.3	0.3
PDB ID code	8K7J	8K7K	8K7L

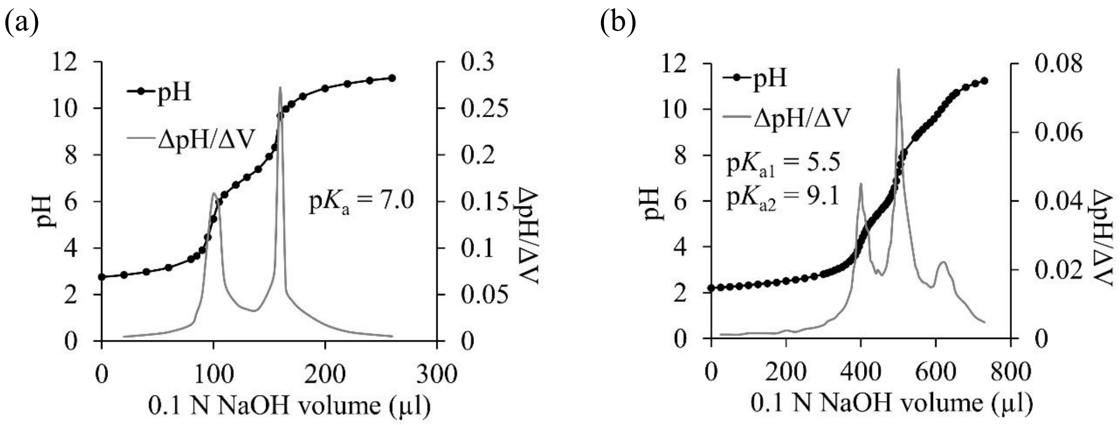

Fig. S2 Titration curves of (a) **4** and (b) **5**.

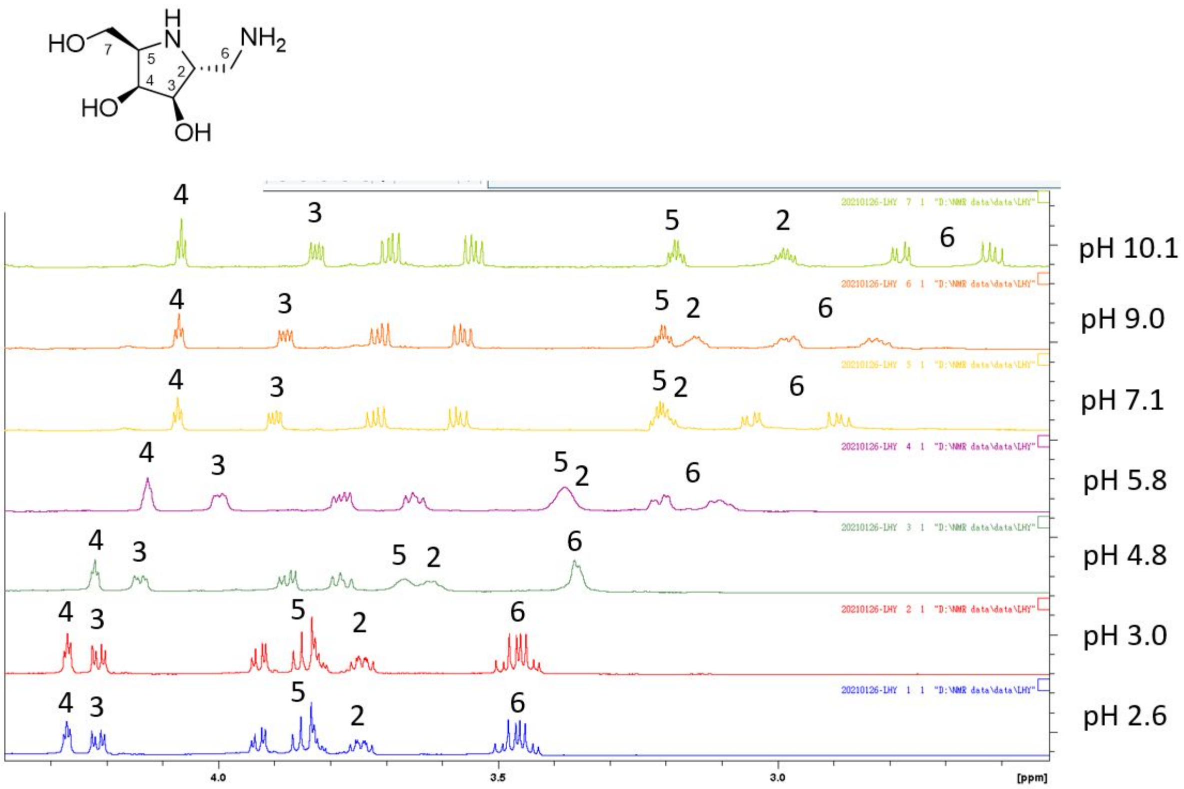

Fig. S3 pH-Dependent ^{1}H-NMR spectra of **5**. The deprotonation of the exocyclic amino group of **5** was reflected by the significantly migrated H-2, H-3, and H-6 chemical shifts from pH 10.1 to 7.1, while the endocyclic amino group by the chemical shifts of all hydrogens from pH 7.1 to 3.0.

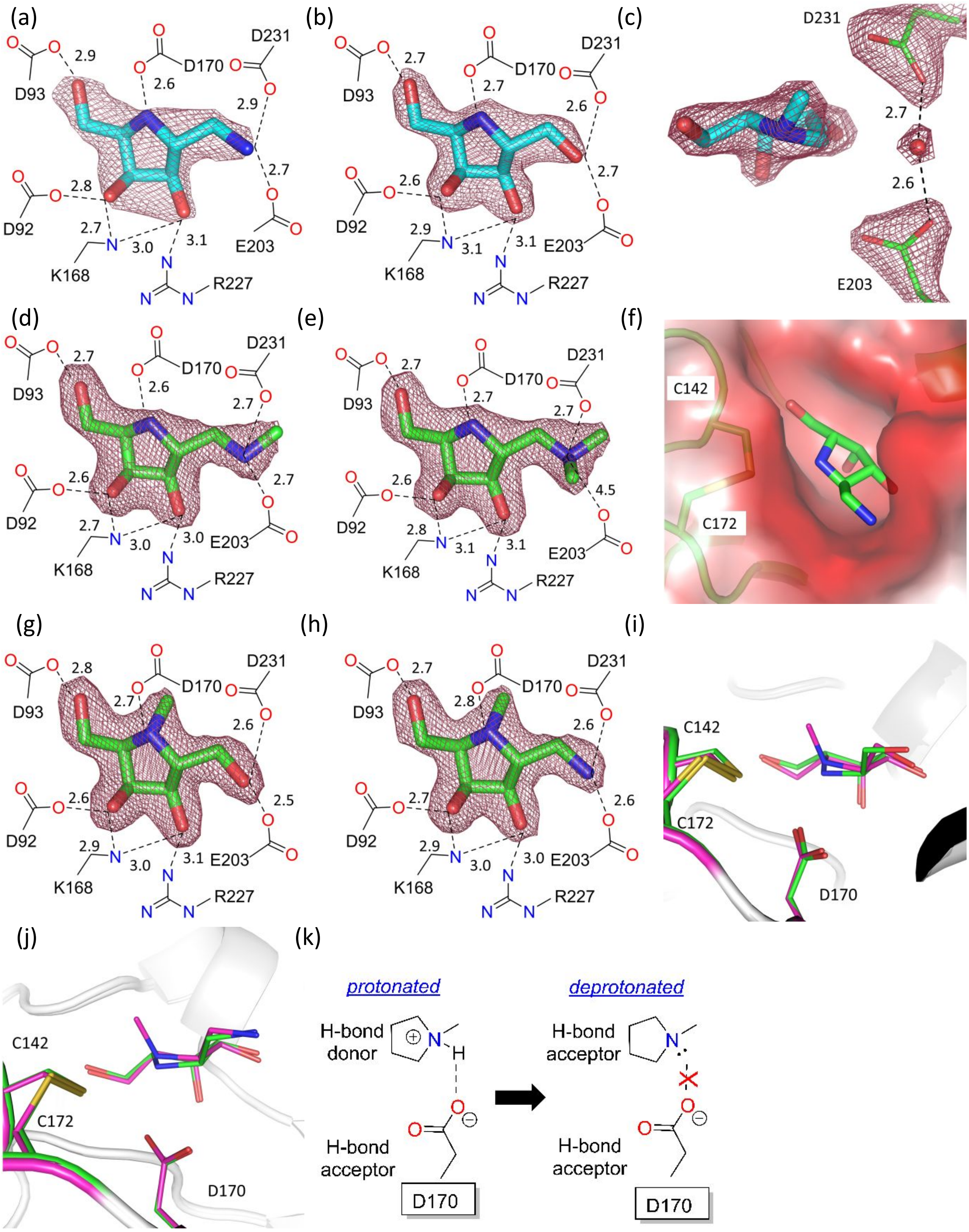

Fig. S4 Crystal structures of α-Gal A bound to the iminosugars. (a) The 2*Fo*-*Fc* electron density around the bound compound **4** at pH 4.5. (b) The 2*Fo*-*Fc* electron density around the bound compound **5** at pH 4.5. (c) The 2*Fo*-*Fc* electron density around the bound compound **8** at pH 7.2. (d) The 2*Fo*-*Fc* electron density around the bound compound **20** at pH 7.2. (e) The 2*Fo*-*Fc* electron density around the bound compound **21** at pH 7.2. (f) Surface representation of the iminosugar-binding pocket with the bound compound **5** being shown with a green stick model.

The surface charge potentials are shown with blue and red colors representing the positive and negative charge, respectively. (g) The 2*Fo*-*Fc* electron density around the bound compound **23** at pH 7.2. (h) The 2*Fo*-*Fc* electron density around the bound compound **24** at pH 7.2. (i) Superimposition of the structures of α-Gal A bound to compounds **4** (green) and **23** (pink). (j). Superimposition of the structures of α-Gal A bound to compounds **5** (green) and **24** (pink). (f) Proposed interactions between the *endo*-N of compound **4** and D170 of α-Gal A.

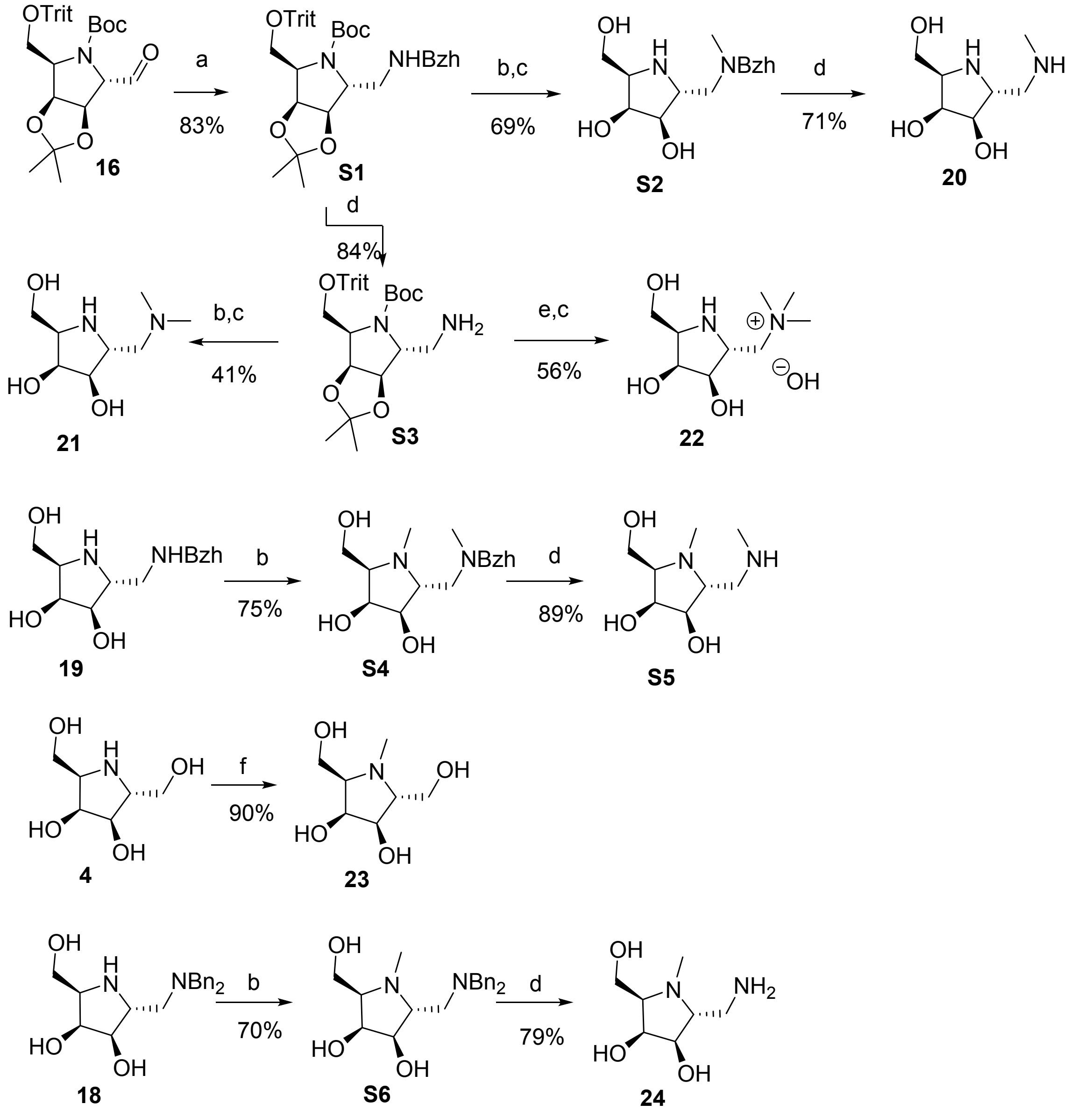

Scheme S1 Synthesis of *N*-methylated pyrrolidines. Reagents and conditions: a) $NaBH_3CN$, NH_2Bzh, AcOH, MeOH, rt, 12 h. b) $NaBH_3CN$, CH_2O, AcOH, MeOH, rt, 12 h. c) 6N HCl, MeOH, rt, 12 h; then DOWEX (OH^-). d) $Pd(OH)_2/C$, H_2, MeOH, rt, 12 h. e) MeI, MeOH, rt, 12 h. f) H_2, $Pd(OH)_2/C$, CH_2O, MeOH, rt, 12 h.

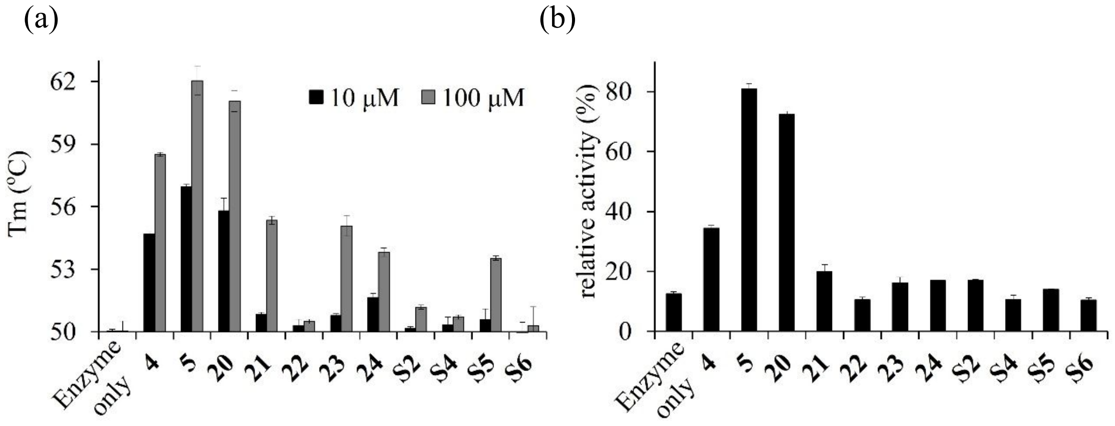

Fig. S5 The protecting effects of iminosugars to α-Gal A. (a) Unfolding Tm values of α-Gal A incubated with or without iminosugars, and monitored by changes in the fluorescence of SYPRO Orange as a function of temperature. (b) The residue activity of α-Gal A determined after incubating with or without 100 μM of pyrrolidines at 37 °C for 30 minutes.

(a)

5 at pH 4.5

Raw data

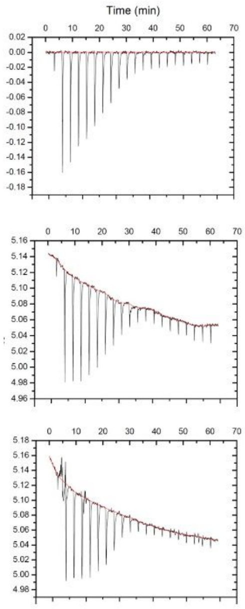

Global fitting

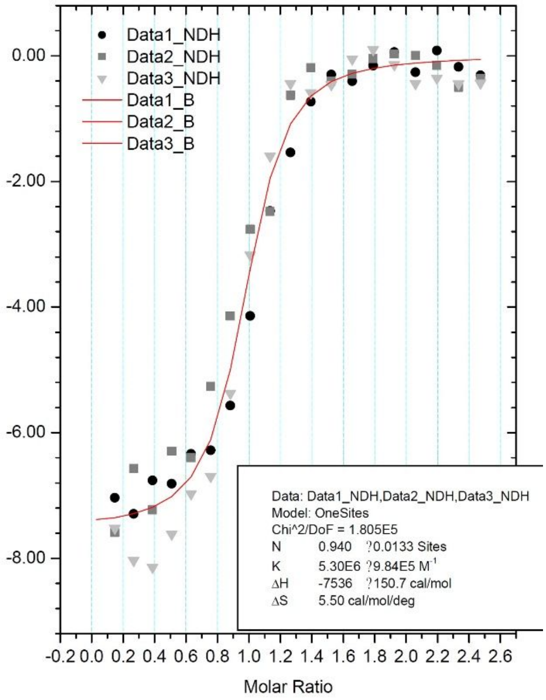

(b)

4 at pH 7.0

Raw data

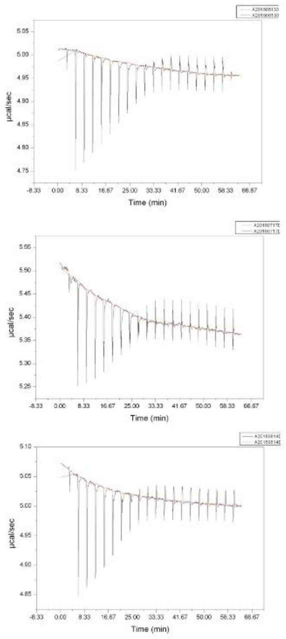

Global fitting

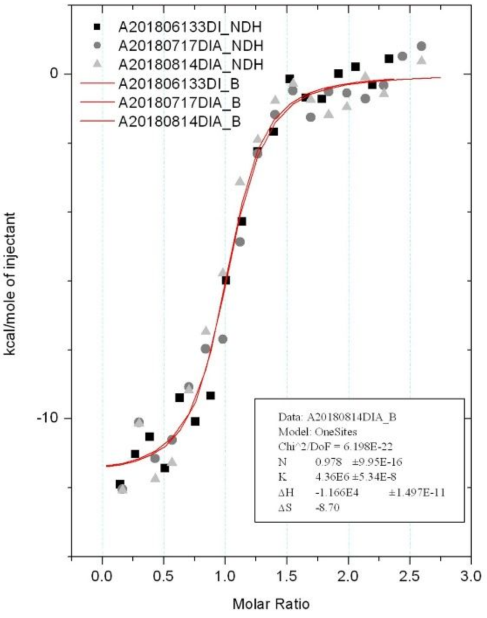

(c)

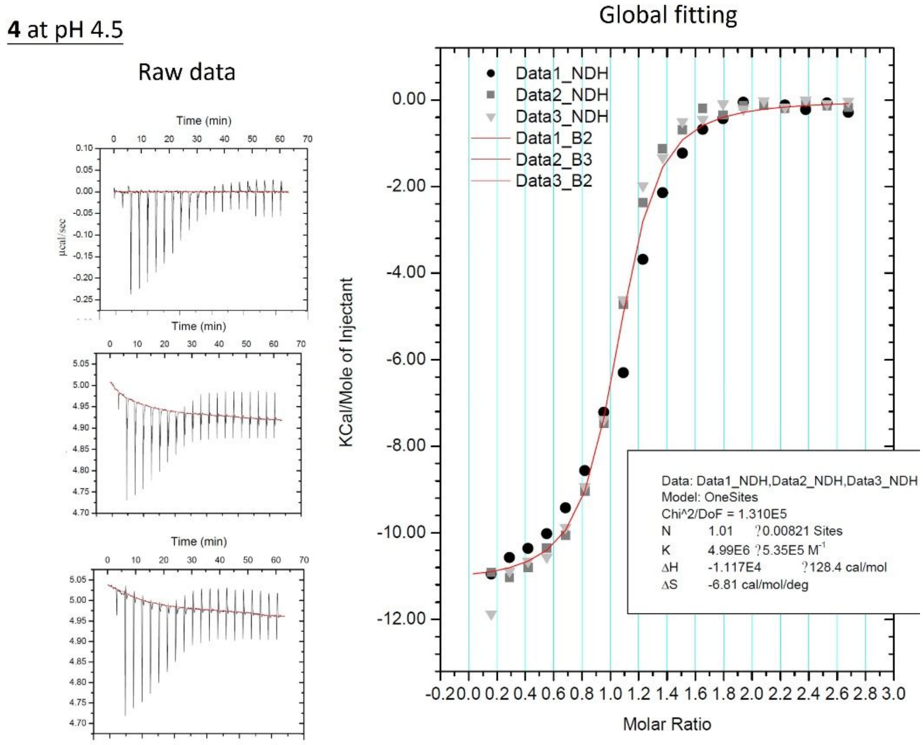

(d)

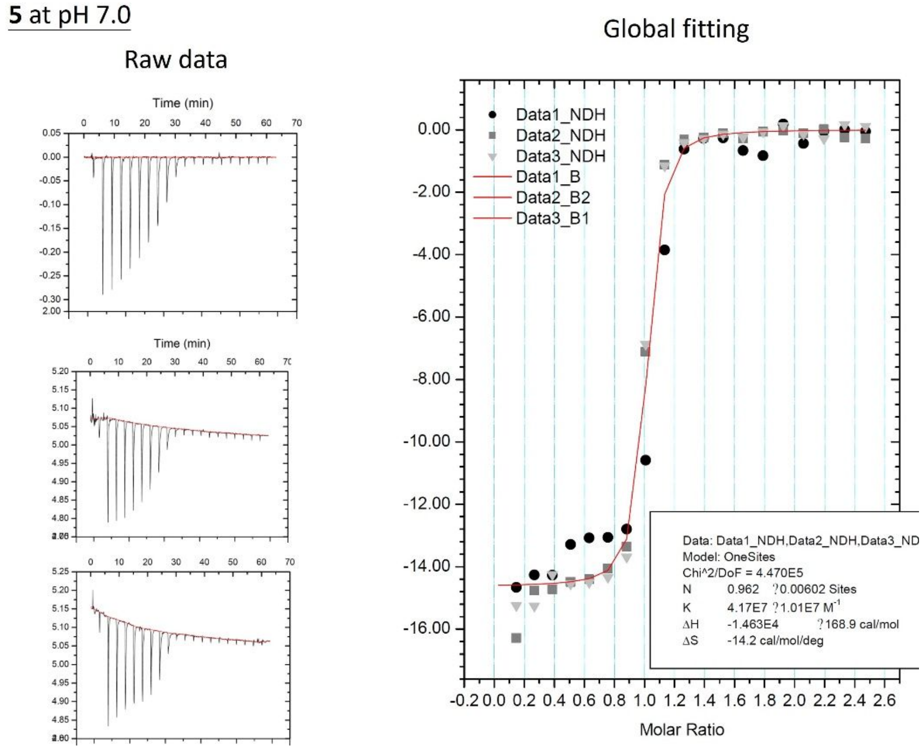

Fig. S6 The raw titration data of the power supplied to the system to maintain a constant temperature against time, and the bimolecular fit of the normalized heats of interaction plotted against the molar concentration. (a) ITC data for **4** at pH 4.5, (b) **4** at pH 7.0, (c) **5** at pH 4.5, and **5** at pH 7.0.

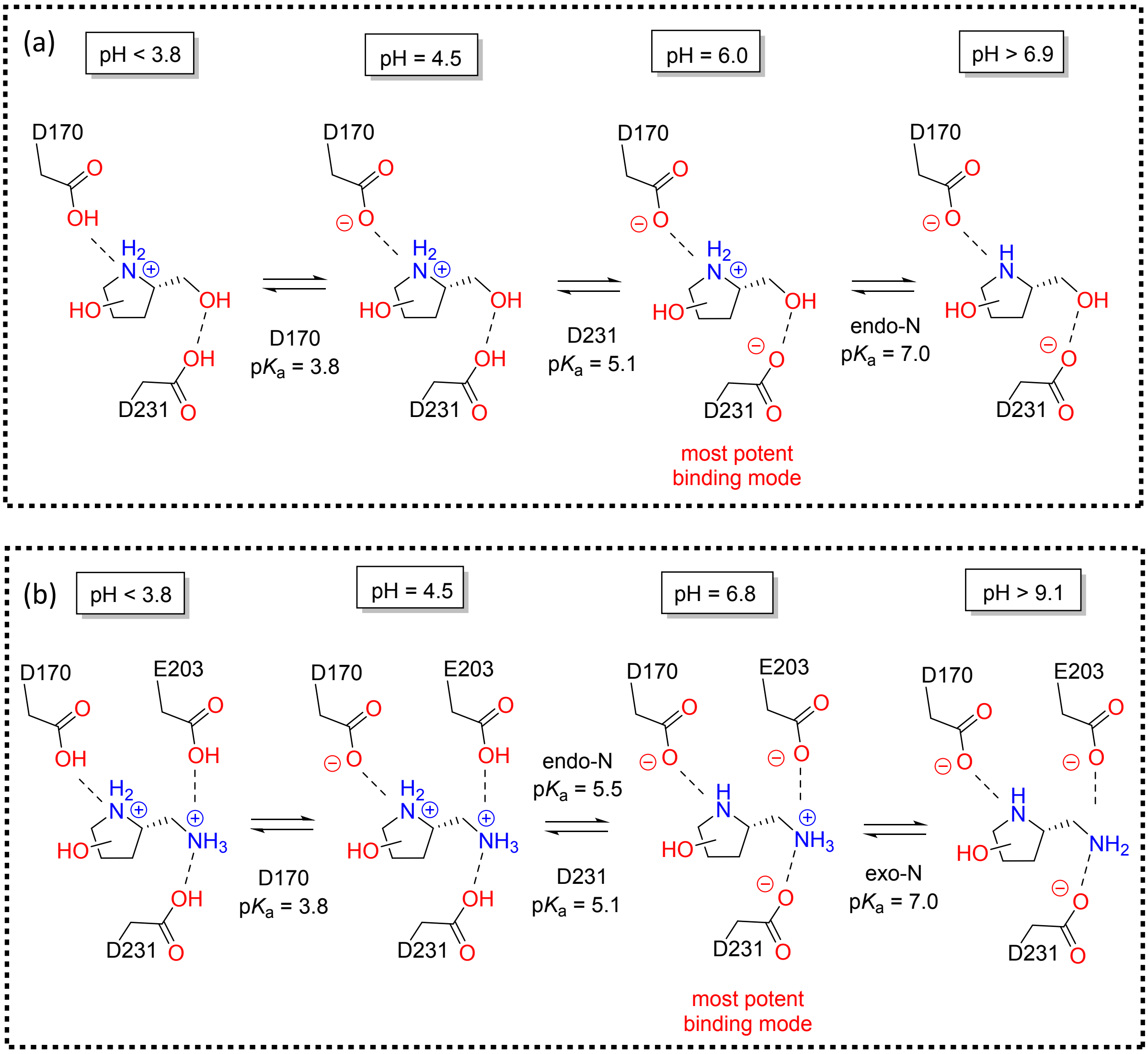

Fig. S7 The proposed protonated states of (a) monobasic iminosugar **4**, and (b) dibasic iminosugar **5** binding to rh-α-Gal A.

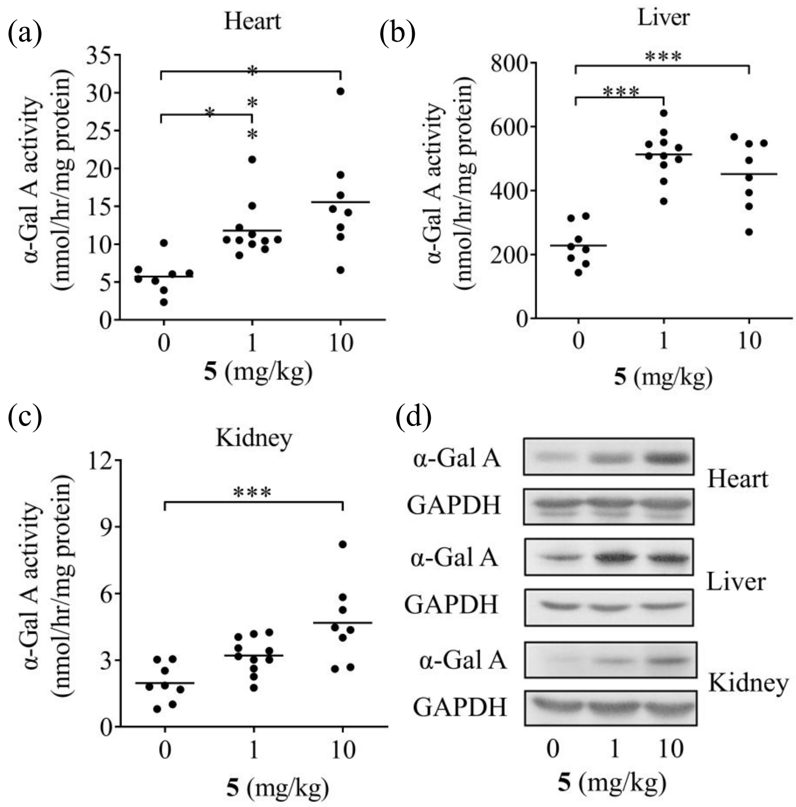

Fig. S8 rh-α-Gal A (3 mg/kg) and **5** (0, 1, 10 mg/kg) were co-administered to mice and sacrificed on day 7. The (a) heart, (b) liver, and (c) kidney were tested for their enzyme activity, and (d) protein level (n=8-11). **p* <0.05, ** *p* <0.01, *** *p* <0.005 (One-way ANOVA with Tukey`s post-hoc)

2. Biochemical and biological methods

Thermal stability shift assay

The stability of rh-α-Gal A was assessed using a modified fluorescence thermal stability assay on a Rotor-Gene system in neutral pH buffer (potassium phosphate, pH 7.4).[1] Briefly, rh-α-Gal A (2 μg) was combined with SYPRO Orange and various concentrations of iminosugars in a final reaction volume of 20 μL. A thermal gradient was applied to the plate at a rate of 1 °C/minute, during which time the fluorescence of SYPRO Orange was continuously monitored. The fluorescence intensity at each temperature was normalized to the maximum fluorescence after complete thermal denaturation.

In vitro stabilization of rh-α-Gal A

An assessment of the ability of iminosugars to stabilize rh-α-Gal A against denaturation was performed.[2] Enzyme aliquots (10 μL, in Dulbecco's Modified Eagle Medium) were incubated with 0, 10, or 100 μM of each molecule on ice for 10 minutes. The samples were heated at 37 °C as a function of time in an attempt to heat-inactivate (denature) rh-α-Gal A, and then the samples were diluted into a 100-fold volume of 0.1 M citric phosphate buffer (pH 4.6). The enzyme was purified by ultrafiltration using a 30 kDa molecular weight cutoff membrane, and then incubated with the substrate (0.1 mM 4-methylumbelliferyl-α-D-galactoside) for 15 minutes at 37°C before quenching with glycine buffer. Liberated 4-methylumbelliferone was measured (excitation 355 nm, emission 460 nm). Enzyme activity was reported relative to the unheated enzyme.

Crystallization and X-ray data collection

Crystals of apo rh-α-Gal A were prepared as described previously.[3, 4] rh-α-Gal A (20 mg/mL in 20 mM Tris-HCl pH 8.0) was mixed with an equal volume of the reservoir solutions: (i) 100 mM NaOAc pH 4.5, 25% PEG4000 and 200 mM ammonium sulfate; (ii) 100 mM Tris-HCl buffer 7.2, 25% PEG4000 and 200 mM ammonium sulfate. Crystals were obtained within 7-14 days by using the hanging drop vapor diffusion method at 20 °C. For the structures of rh α-Gal A in complex with iminosugars, the crystals were soaked into 5-50 mM for 5 minutes. Prior to syn X-ray data collection, the crystals were transferred to a solution containing the mother liquid supplemented with 15% glycerol (v/v) and then flash-frozen in liquid nitrogen. The X-ray diffraction data were collected at the beamlines 13B1 and 15A1 of the Taiwan Light Source, or the beamline 05A of the Taiwan Photon Source, National Synchrotron Radiation Research Center, Hsinchu, Taiwan. All diffraction data were processed with the program *HKL-2000*.[5] The data collection statistics are listed in Table S1. The space group of the crystals is $P3_221$ with the typical unit cell dimensions of a = 90.0 Å, b = 90.0 Å, c = 216.0 Å. The asymmetric unit comprises an rh-α-Gal A dimer with an estimated solvent content of ~54%.

Structure determination and refinement

The crystal structures of rh-α-Gal A bound to the iminosugars were solved by the molecular replacement phasing method with the program *Molrep* within the *CCP4* software suite,[6] and using the published α-Gal A structure (PDB code: 1R46) as a template. The initial models were subjected to the manual adjustment with *Coot* and then computational refinement with *Refmac5*.[7, 8] Throughout refinement, a randomly selected 5% of the data was set aside as a free data set, and the model was refined against the remaining data with F> 0 as a working data set. Subsequently, iterative rounds of model adjustment with *Coot* and refinement with *REFMAC5* were performed to improve the quality and completeness of the structures until the *R*-factor and R_{free} values were converged. The stereochemical quality of the refined structure was checked with the program *MolProbity*.[9] The final refinement statistics are listed in Table S1. The molecular figures were generated with *PyMOL* (Schrödinger, New York, USA).

Determination of the pK_a values of iminosuigars 4 and 5

pK_a values of the basic iminosugars **4** and **5** were calculated from titration curves according to the Henderson–Hasselbach approximation. The titration curves were obtained by titrating aqueous solutions of the inhibitors with sodium hydroxide solution.

Isothermal titration calorimetry

All the titration experiments were performed using MicroCal iTC200 according to the method described previously.[10] Briefly, rh-α-Gal A and ligands were prepared in 100 mM NaOAc buffer (pH 4.5) or 100 mM phosphate buffer (pH 7.0) before use. The final protein concentration in the sample cell was determined by the BCA protein assay kit (Thermo Fisher Scientific). The integrated heat data were fit with a one-site binding model using the *Origin-7* software. Protein concentration was corrected by titration of the reference compound and normalized for the concentration of each ligand. All ligands bound to rh-α-Gal A have a stoichiometry of approximate unity.

pH-dependent inhibition

Values of k_{cat}/K_m and $1/K_i$ as a function of pH for the reaction of rh-α-Gal A with 4-methylumbelliferyl-α-D-galacopyranoside ([S] << K_m) were performed at 37 °C and 100 mM buffer for 30 minutes. 100 mM Citrate-phosphate was used for pH 3.0 to 7.5.[11] The released 4-methylumbelliferone was detected as described previously. The data were fitted with a bell ionization curve (limit × $10^{(pH-pKa1)}$ / ($10^{(2\times pH-pKa1-pKa2)}$ + $10^{(pH-pKa1)}$ + 1)) using the software GraFit to determine the pK_a values. K_i values are calculated (IC_{50}/K_i = (1+[S]/K_m) from the average of triplicate independent measurements of IC_{50}. The dependence $1/K_i$ versus pH was also fitted with a bell ionization curve GraFit.

Cell-based rh-α-Gal A activity identification

Fabry W162X fibroblast (GM00107, Coriell Institute) or N215S (GM04391, Coriell Institute) was seeded in sterile, clear-bottom, 48-well plates at 20000 cells/well, and incubated at 37 °C, 5% CO_2 for 12–16 hours.[2, 12] The cells were then incubated with rh-α-Gal A (1, 10, 100, 1000 nM) alone, or with iminosugars for 24 hours. The cells were washed three times with growth medium, and then maintained in growth medium at 37 °C, 5% CO_2 for another day. The enzyme assay was performed after being washed twice with PBS, the cells were treated with 50 λ reaction buffer (0.1% triton X 100, 8 mM 4-methylumbelliferyl-α-galactoside, 150 mM *N*-acetylgalactosamine in pH 4.6 citric phosphate buffer) followed by incubation at 37 °C for 1h. Stop solution (0.4 M K_2CO_3, pH 10.8) was then added and fluorescence was read on a Victor plate reader (at 355 nm excitation and 460 nm emission). Raw fluorescence counts were background subtracted, as defined by counts from substrate solution only. A MicroBCA Protein Assay Kit was used to determine protein concentration of the cell lysates. 4-Methylumbelliferone standard curve ranging from 0 to 200 μM was determined in the same day for conversion of fluorescence data to absolute rh-α-Gal A activity expressed as the nanomoles of 4-methylumbelliferone liberated per mg protein/hour (nmol/mg protein/hour). Relative α-Gal A activity was expressed as fold which was normalized to untreated enzyme activity.

Cell-based Gb3 quantification

Fabry W162X fibroblast was seeded in sterile, clear-bottom, 48-well plates at 20000 cells / well, and incubated at 37 °C, 5% CO_2 for 12–16 h. After the desired treatment, the cells were washed three times with PBS and extracted with MeOH containing the unnatural Gb3 (C17:0) as the internal standard for 0.5 h. The supernatant was collected and concentrated by speedvac, and the residue was dissolved with DMSO for the Gb3 quantification by LC-MS/MS to measure the signal of six most representative isoforms (C16:0, C20:0, C22:0, C24:1, C24:0, C24:1-OH) as the total amount of Gb3 in treated Fabry W162X fibroblasts.[13]

Cell imaging

Cells were washed twice with PBS and fixed in Cytofix/Cytoperm™ solution containing 4% paraformaldehyde (BD Biosciences, cat.554722) at room temperature for 20 minutes. The cells were then permeabilized with Perm/Wash buffer (BD Biosciences, cat.554723) at room temperature for 5 minutes. The cells were then washed twice with wash buffer and blocked in 5% BSA and incubated at room temperature for 1 hour, followed by incubation with primary antibodies (Rabbit anti-GLA, GTX101178; Mouse anti-LAMP1, GTX80229) at 4 °C for overnight. The cells were washed with wash buffer and incubated with secondary antibodies (Goat anti-rabbit IgG antibody, GTX213110-05; Goat anti-mouse IgG antibody, GTX213111-

04) at room temperature for 1 hour, and nuclei-stained with DAPI for 20 minutes. Images were acquired by using Nikon Ni-E fluorescence microscope and quantified by using ImageJ software. Fluorescence intensity was defined as GLA fluorescence/cell number under 10 low-power field. Scale bar, 20 mm.

rh-α-Gal A activity in *Gla* KO mice

All *in vivo* experiments were conducted in accordance with the regulations and guidelines of Institutional Animal Care and Use Committee︶IACUC︵of Taipei Veterans General. 8-12 Weeks old male *Gla* KO mice were treated rh-α-Gal A (3 mg/kg) and small molecules (0, 1, or 10 mg/kg) by intravenous tail. Mice were euthanized 1 or 7 days after treatment as indicated. Mouse tissues (heart, liver, and kidney) were weighted and homogenized in 0.1% Triton (0.1 mg/ml). The rh-α-Gal A activity, protein concentration and rh-α-Gal A levels were detected as previously described. The half-life of rh-α-Gal A was calculated using the formula: $t_{1/2} = \log 0.5/(\log A_e/A_0) \times t$, where $t_{1/2}$ is the half-life of the rh-α-Gal A, A_e is the amount of rh-α-Gal A remaining, A_0 is the amount of rh-α-Gal A at 15 minutes, and t is the elapsed time.[14]

Western blot analysis

Equal amounts of protein were subjected to SDS-PAGE followed by transfer onto PVDF membrane. The membrane was blocked with 5% skim milk and probed with indicated primary antibodies (anti-galactosidase alpha antibody, GTX101178; anti-GAPDH, GTX100118) at 4 ^{o}C overnight. Following PBST washes, membranes were incubated in HRP-conjugated secondary antibodies at room temperature for 1 hour. Blots were detected by HRP Chemiluminescent Substrates (PerkinElmer, US), and the signals were measured using UVP biospectrum.

3. Chemical Synthesis

General imformation

All chemicals were obtained from commercial suppliers and used without further purification. Reactions were magnetically stirred and monitored by thin-layer chromatography on silica gel. Column chromatography was performed using silica gel (Merck Kieselgel Si60 (40–63 μm)). Thin layer chromatography (TLC) was performed on glass plates coated to a thickness of 1 mm with Merck Kieselgel 60F254. ^{1}H NMR (600 MHz) and ^{13}C NMR (150 MHz) spectra were recorded on a Bruker AM-600 NMR spectrometer using $CDCl_3$, MeOD or D_2O as solvents. Chemical shifts are given in parts per million (ppm) and *J* (coupling constant) values were estimated in Hertz (Hz). High resolution mass spectra (HRMS) were measured with a Bruker BioTOF III (ESI-MS).CC refers to column chromatography. Concentration refers to rotary evaporation. THF refers tetrahydrofuran. DMSO refers dimethyl sulfoxide. Cyclic nitrone **6** wase obtained through previous methods.[15]

Synthesis of compounds 7 and 8

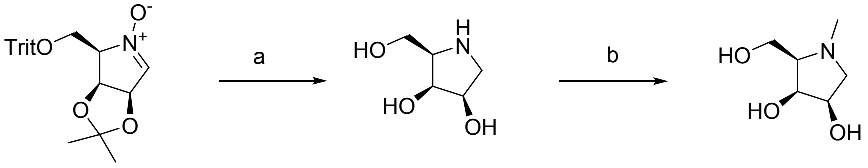

6 **7** **8**

Scheme S2. Synthesis of compounds **7** and **8**. Reagents and conditions: a) 1. Raney Ni, H_2, MeOH, rt, 12 h; 2. 6N HCl, MeOH, rt, 12 h; then DOWEX (OH^-), 74% over two steps; b) H_2, $Pd(OH)_2/C$, CH_2O, MeOH, rt, 12 h, 82%.

(2*R*,3*S*,4*R*)-2-(hydroxymethyl)pyrrolidine-3,4-diol (7)[16]

Nitrone **6** (100 mg, 0.23 mmol) was treated with Raney Ni in MeOH (1 mL) under hydrogen atmosphere and the reaction was stirred at room temperature for 12 h. The mixture was filtered, concentrated, and directly used for the next step. The residue was dissolved in MeOH containing 6N HCl, and stirred at room temperature for 12h. The mixture was quenched with resin (DOWEX 550A) concentrated and purified by CC to afford amine **7** (23 mg, 0.17 mmol, 74% over two steps) as a yellowish oil. TLC: $R_f = 0.3$ (*n*-propanol/$NH_{3(aq)}$, 8/1, v/v). $[\alpha]_D^{23} = +10.4$ ($c = 0.1$, H_2O). ^{1}H NMR (600 MHz, MeOD) δ 3.16 (dd, 1H, $J = 6.8$, 11.6 Hz), 3.42 (dd, 1H, $J = 7.3$, 11.6 Hz), 3.62–3.66 (m, 1H), 3.90 (dd, 1H, $J = 9.1$, 11.8 Hz), 3.96 (dd, 1H, $J = 4.6$, 11.8 Hz), 4.22 (t, 1H, $J = 4.4$), 4.39–4.42 (m, 1H); ^{13}C NMR (150 MHz, MeOD) δ 70.5, 70.0, 63.2, 57.9, 47.1; HRMS calcd. for $[C_5H_{11}NO_3 + H]^+$ 134.0812, found 134.0814.

(2*R*,3*S*,4*R*)-2-(hydroxymethyl)-1-methylpyrrolidine-3,4-diol (8)

Amine **7** (10 mg, 0.075 mmol) was treated with formaldehyde (68 μL, 0.75 mmol) and $Pd(OH)_2/C$ in MeOH (0.2 mL) under hydrogen atmosphere and the reaction was stirred at room temperature for 12 h. The mixture was concentrated and purified by CC to afford **8** (9 mg, 0.061 mmol, 82%) as a white solid. TLC: R_f = 0.3 ($CHCl_3$/MeOH/$NH_{3(aq)}$, 88/58/13.5, v/v). $[\alpha]_D^{23}$ = -7.9 (*c* = 0.1, H_2O). ^1H NMR (600 MHz, D_2O) δ 3.05 (s, 3H), 3.41 (dd, 1H, *J* = 5.3, 12.5 Hz), 3.66 (dd, 1H, *J* = 3.9, 12.5 Hz), 3.72 (dd, 1H, *J* = 5.8, 10.9 Hz), 4.03 (dd, 1H, *J* = 6.12, 12.8 Hz), 4.08 (dd, 1H, *J* = 4.6, 12.8 Hz), 4.61–4.64 (m, 2H); ^{13}C NMR (150 MHz, D_2O) δ 70.8, 70.3, 68.9, 58.9, 57.3, 41.5; HRMS calcd. for $[C_6H_{13}NO_3 + H]^+$ 148.0968, found 148.0969.

Synthesis of compounds 10-13

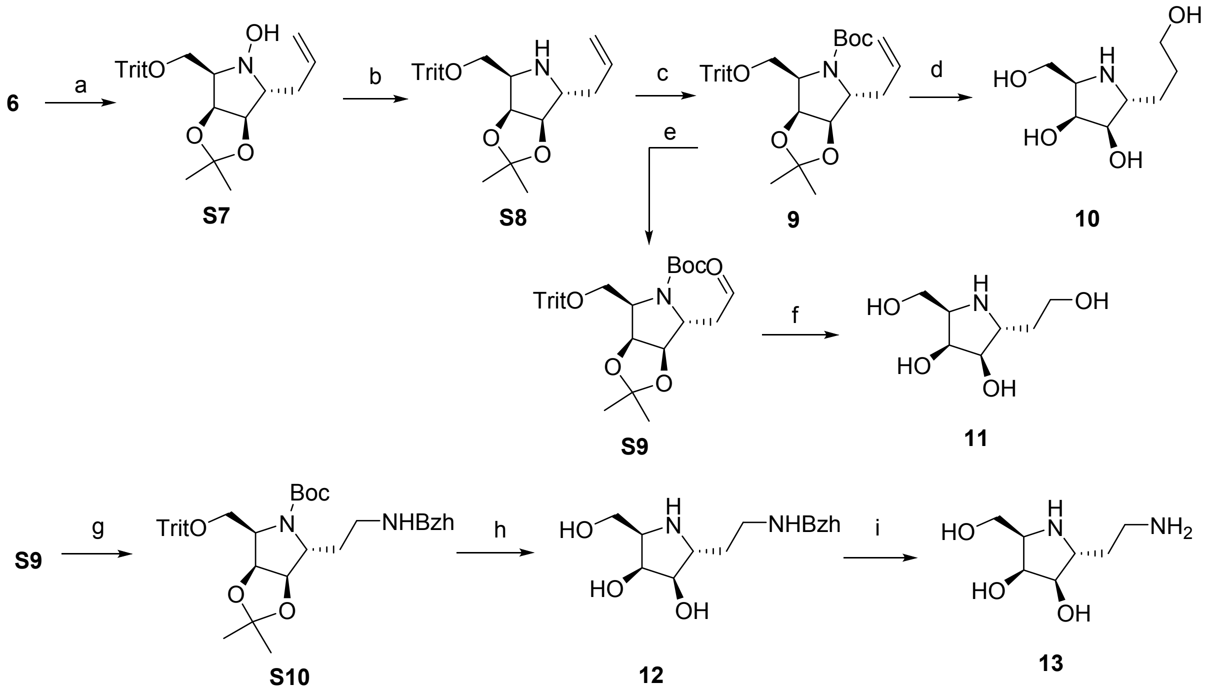

Scheme S3. Synthesis of compounds **10-13.** Reagents and conditions: a) AllylMgCl, THF, 0 °C, 2 h, 98%; b) Zn, AcOH, CH_2Cl_2, rt, 12 h, 96%; c) Boc_2O, Et_3N, MeOH, rt, 12 h, 82%; d) 1. BH_3, THF, rt, 1h; then H_2O_2, NaOH, rt, 12h; 2. 6N HCl, MeOH, rt, 12 h; then DOWEX (OH^-), 48% over two steps; e) O_3, CH_2Cl_2, -78 °C, 0.5 h, then Me_2S, 90%; f) 1. $NaBH_4$, MeOH, rt, 12 h; 2. 6N HCl, MeOH, rt, 12 h; then DOWEX (OH^-), 68% over two steps; g) $NaBH_3CN$, benzhydrylamine, AcOH, MeOH, rt, 12 h, 91%; h) 6N HCl, MeOH, rt, 12 h; then DOWEX (OH^-), 75%; i) $Pd(OH)_2/C$, H_2, MeOH, rt, 12 h, 84%.

(3a*R*,4*R*,6*R*,6a*S*)-4-allyl-2,2-dimethyl-6-((trityloxy)methyl)tetrahydro-5*H*-[1,3]dioxolo[4,5-c]pyrrol-5-ol (S7)

Cyclic nitrone **6** (1.35 g, 3.14 mmol) was dissolved in dry THF (20 mL), and then allyl

magnesium chloride (1 M in THF, 3 eq.) was added dropwisely at 0 °C. After 1 h, the mixture was quenched with $NH_4Cl_{(aq)}$, extracted with EtOAc, dried with $MgSO_4$, concentrated, and purified by CC to give alkene **S7** (1.44 g, 3.08 mmol, 98%) as a white solid. TLC: R_f = 0.3 (EtOAc/n-hexanes, 1/3, v/v). ^{1}H NMR (600 MHz, $CDCl_3$) δ 1.26 (s, 6H), 1.82–1.87(m, 1H), 2.65–2.67 (m, 1H), 3.17-3.19 (m, 1H), 3.36 (dd, 1H, J = 8.7, 5.7 Hz), 3.41 (dd, 1H, J = 10.68, 4.14 Hz), 3.55 (t, 1H, J = 8.52 Hz), 4.43 (d, 1H, J = 6.78 Hz), 4.73 (t, 1H, J = 6.18 Hz), 5.07–5.10 (m, 2H), 5.69-5.76 (m, 1H), 7.19–7.48 (m, 15H); ^{13}C NMR (150 MHz, $CDCl_3$) δ 24.5, 25.8, 61.2, 67.0, 69.3, 80.5, 86.9, 111.3, 117.6, 126.9-128.8 (× 18) 134.8, 144.0; HRMS calcd. for $[C_{27}H_{28}N_2O_4 + Na]^+$ 467.1941, found 467.1952.

(3a*R*,4*R*,6*R*,6a*S*)-4-allyl-2,2-dimethyl-6-((trityloxy)methyl)tetrahydro-4*H*-[1,3]dioxolo[4,5-c]pyrrole (S8)

Alkene **S7** (500 mg, 1.07 mmol) was treated with Zn (700 mg, 10.7 mmol) and AcOH (1.0 mL, 17.1 mmol) in CH_2Cl_2 (5 mL), and stirred at room temperature for 12h. After quenched with $NaHCO_{3(aq)}$, the mixture was extracted with CH_2Cl_2, dried with $MgSO_{4(s)}$, concentrated, and purified by CC to give amine **S8** (468 mg, 1.03 mmol, 96%) as a white solid. TLC: R_f = 0.2 (EtOAc/n-hexanes, 1/3, v/v). ^{1}H NMR (600 MHz, $CDCl_3$) δ 1.35 (s, 3H), 1.41 (s, 3H), 2.14–2.16 (m, 3H), 3.19 (dd, 1H, J = 7.7, 7.8 Hz), 3.26–3.31 (m, 2H), 3.46 (t, 1H, J = 6.4 Hz), 4.47 (d, 1H, J =5.8 Hz), 4.75 (dd, 1H, J = 4.7, 5.3 Hz), 5.12–5.16 (m, 2H), 5.79–5.87 (m, 1H), 7.25–7.28 (m, 3H), 7.32–7.35 (m, 6H), 7.53–7.54 (m, 6H); ^{13}C NMR (150 MHz, $CDCl_3$) δ 144.2 (× 3), 135.3, 128.9 (× 6), 127.7 (× 6), 126.9 (× 3), 117.3, 111.2, 86.7, 85.1, 80.9, 62.6, 62.2, 60.1, 37.1, 27.1, 24.8; HRMS calcd. for $[C_{30}H_{33}NO_3 + H]^+$ 456.2533, found 456.2534.

tert-butyl(3a*R*,4*R*,6*R*,6a*S*)-4-allyl-2,2-dimethyl-6-((trityloxy)methyl)tetrahydro-5*H*-[1,3]dioxolo[4,5-c]pyrrole-5-carboxylate (9)

Amine **S8** (200 mg, 0.44 mmol) was treated with Boc_2O (294 µL, 1.31 mmol) and Et_3N (1926 µL, 1.31 mmol) in CH_2Cl_2 (2 mL), and was stirred at room temperature for 12h. The mixture was extracted with CH_2Cl_2, dried with $MgSO_{4(s)}$, concentrated, and purified by CC to give alkene **9** (208 mg, 0.36 mmol, 82%) as a colorless oil. TLC: R_f = 0.5 (EtOAc/n-hexanes, 1/5, v/v). ^{1}H NMR (600 MHz, $CDCl_3$) δ 1.25–1.44 (m, 15H), 2.23–2.45 (m, 2H), 3.51 (t, 1H, J = 8.6 Hz), 3.68–3.73 (m, 1H), 3.87–4.28 (m, 2H), 4.46 (br, 1H), 4.95 (br, 1H), 4.14–4.18 (m, 2H), 5.72–5.79 (m, 1H), 7.25–7.28 (m, 3H), 7.32–7.35 (m, 6H), 7.53–7.56 (m, 6H); ^{13}C NMR (150 MHz, $CDCl_3$) δ 154.2, 144.4 (× 3), 133.7, 128.9 (× 6), 127.7 (× 6), 126.9 (× 3), 118.4, 111.2, 86.8, 81.5, 80.7, 80.1, 64.0, 61.8, 60.5, 35.7, 28.4 (× 3), 26.5, 25.4; HRMS calcd. for $[C_{35}H_{41}NO_5 + Na]^+$ 578.2877, found 578.2876.

(2*R*,3*S*,4*R*,5*R*)-2-(hydroxymethyl)-5-(3-hydroxypropyl)pyrrolidine-3,4-diol (10)

Alkene **9** (200 mg, 0.35 mmol) was dissolved in anhydrous THF (2 mL), treated with BH_3·THF (1 M in THF, 4 eq.) at 0 °C. The mixture was warmed to room temperature and stirred for 3 h, then cooled to 0 °C, and $NaOH_{(aq)}$ (2 M, 16 eq.) followed by H_2O_2 (30 %, 24 eq.) were added. The reaction was stirred at room temperature for 12 h. After quenched with $Na_2S_2O_{3(aq)}$, the mixture was extracted with Et_2O, dried with $MgSO_{4(s)}$, filtered, concentrated, and purified by CC to obtain the hydroxylated intermediate. The intermediate was dissolved in MeOH with 6N HCl, and stirred at room temperature for 12 h. The mixture was quenched with resin (DOWEX 550A), filtered, concentrated, and purified by CC to give **10** (32 mg, 0.17 mmol, 48% over two steps) as colorless oil. TLC: R_f = 0.5 (*n*-propanol/$NH_{3(aq)}$, 7/1, v/v). $[\alpha]_D^{23}$ = +37.8 (*c* = 0.1, H_2O). ^1H NMR (600 MHz, D_2O) δ 1.63–1.72 (m, 2H), 1.75–1.85 (m, 1H), 1.89–1.98 (m, 1H), 3.51 (ddd, 1H, *J* = 5.5, 9.1, 14.6 Hz), 3.60–3.63 (m, 2H), 3.77–3.82 (m, 1H), 3.86 (dd, 1H, *J* = 8.3, 12.1 Hz), 3.96 (dd, 1H, *J* = 4.9, 12.1 Hz), 4.15 (dd, 1H, *J* = 3.9, 9.1 Hz), 4.30 (dd, 1H, *J* = 3.6, 3.9 Hz); ^{13}C NMR (150 MHz, D_2O) δ 77.1, 71.7, 61.4, 60.0, 59.5, 59.4, 29.2, 28.6; HRMS calcd. for $[C_8H_{17}NO_4 + H]^+$ 192.1230, found 192.1233.

tert-butyl(3a*R*,4*R*,6*R*,6a*S*)-2,2-dimethyl-4-(2-oxoethyl)-6-((trityloxy)methyl)tetrahydro-5*H*-[1,3]dioxolo[4,5-c]pyrrole-5-carboxylate (S9)

Alkene **9** (500 mg, 0.9 mmol) was ozonolysis in CH_2Cl_2 (5 mL) at -78 ^oC for 10 min, and then treated with Me_2S (330 µL, 4.5 mmol), concentrated, and purified by CC to give aldehyde **S9** (464 mg, 0.8 mmol, 90%) as a white solid. TLC: R_f = 0.3 (EtOAc/n-hexanes, 1/5, v/v). ^1H NMR (600 MHz, $CDCl_3$) δ 1.21–1.45 (m, 15H), 2.65–2.95 (m, 2H), 3.51 (t, 1H, *J* = 7.8 Hz), 3.73–4.01 (m, 2H), 4.22 (s, 1H), 4.43 (d, 1H, *J* = 4.8 Hz), 5.01 (s, 1H), 7.25–7.28 (m, 3H), 7.31–7.34 (m, 6H), 7.53–7.55 (m, 6H), 9.75 (s, 1H); ^{13}C NMR (150 MHz, $CDCl_3$) δ 199.5, 153.9, 144.3 (× 3), 128.9 (× 6), 127.7 (× 6), 126.9 (× 3), 111.7, 86.8, 82.3, 80.6, 80.0, 61.3, 60.3, 59.9, 44.8, 28.3 (× 3), 26.7, 25.3; HRMS calcd. for $[C_{34}H_{39}NO_6 + Na]^+$ 580.2670, found 580.2668.

(2*R*,3*R*,4*S*,5*R*)-2-(2-hydroxyethyl)-5-(hydroxymethyl)pyrrolidine-3,4-diol (11)

Aldehyde **S9** (200 mg, 0.37 mmol) was dissolved in MeOH (2 mL), treated with $NaBH_4$ (42 mg, 1.11 mmol) at 0 ^oC, and then stirred for 1 h. After quenched with NH_4Cl, the mixture was extracted with EtOAc, dried with $MgSO_{4(s)}$, and concentrated. The residue was dissolved in MeOH (2 mL) with 6N HCl, and stirred for 12 h at room temperature. The mixture was quenched with resin (DOWEX 550A), filtered, concentrated, and purified by CC to give **11** (43 mg, 0.24 mmol, 68% yield over two steps) as a colorless oil. TLC: R_f = 0.2 (*n*-propanol/NH-$_{3(aq)}$, 8/1, v/v). $[\alpha]_D^{23}$ = +57.6 (*c* = 0.1, H_2O). ^1H NMR (600 MHz, D_2O) δ 1.75–1.77 (m, 1H), 1.87–1.93 (m, 1H), 3.25 (ddd, 1H, *J* = 4.8, 9.0, 13.7 Hz), 3.48 (m, 1H), 3.59–3.68 (m, 3H), 3.78 (dd, 1H, *J* = 6.0, 11.6 Hz), 3.91 (dd, 1H, *J* = 4.1, 9.1 Hz), 4.15 (dd, 1H, *J* = 3.8, 4.1 Hz); ^{13}C NMR (150 MHz, D_2O) δ 76.3, 70.7, 60.3, 59.0, 58.9, 57.6, 33.8; HRMS calcd. for

$[C_7H_{15}NO_4 + H]^+$ 178.1074, found 178.1065.

tert-butyl(3a*R*,4*R*,6*R*,6a*S*)-4-(2-(benzhydrylamino)ethyl)-2,2-dimethyl-6-((trityloxy)methyl)tetrahydro-5*H*-[1,3]dioxolo[4,5-c]pyrrole-5-carboxylate (S10)

Aldehyde **S9** (500 mg, 0.86 mmol) was treated with $NaBH_3CN$ (163 mg, 2.59 mmol), benzhydrylamine (447 μL, 2.59 mmol), and AcOH (155 μL, 2.59 mmol) in MeOH (10 mL), and stirred at room temperature for 12 h. The mixture was quenched with 1N $NaOH_{(aq)}$, extracted, concentrated, and purified by CC to give compound **S10** (567 mg, 0.78 mmol, 91%) as a colorless oil. TLC: $R_f = 0.6$ (EtOAc/n-hexanes, 1/5, v/v). ^1H NMR (600 MHz, $CDCl_3$) δ 1.22–1.36 (m, 15H), 1.55–1.91 (m, 2H), 2.60–2.62 (m, 2H), 3.56 (s, 2H), 3.90–4.28 (m, 2H), 4.37 (d, 1H, J = 4.9), 4.81 (s, 1H), 4.91 (s, 1H), 7.22–7.35 (m, 13H), 7.40–7.43 (m, 6H), 7.52–7.53 (m, 6H); ^{13}C NMR (150 MHz, $CDCl_3$) δ 154.3, 144.4 (× 3), 144.0, 143.9, 128.9 (× 6), 128.5 (× 4), 127.7 (× 6), 127.3 (× 4), 127.0 (× 3), 126.9 (× 2), 111.2, 86.8, 81.8, 80.7, 80.1, 67.7, 62.9, 61.6, 59.9, 44.7, 30.6, 28.3 (× 3), 26.5, 25.3; HRMS calcd. for $[C_{47}H_{52}N_2O_5 + H]^+$ 725.3949, found 725.3957.

(2*R*,3*R*,4*S*,5*R*)-2-(2-(benzhydrylamino)ethyl)-5-(hydroxymethyl)pyrrolidine-3,4-diol (12)

Compound **S10** (100 mg, 0.14 mmol) was dissolved in MeOH (1 mL) with 6N HCl, and stirred at room temperature for 12 h. The mixture was quenched with resin (DOWEX 550A), filtered, concentrated, and purified by CC to give **12** (35 mg, 0.10 mmol, 75%) as colorless oil. TLC: $R_f = 0.3$ (CH_2Cl_2/MeOH, 4/1, v/v). $[\alpha]_D^{23} = +27.4$ (c = 0.1, DMSO). ^1H NMR (600 MHz, D_2O) δ 1.87–1.91 (m, 1H), 1.95–2.05 (m, 1H), 2.78 (m, 2H), 3.28 (dd, 1H, J = 7.8, 14.9 Hz), 3.50–3.53 (m, 1H), 3.67 (dd, 1H, J = 8.0, 11.9 Hz), 3.79 (dd, 1H, J = 5.5, 11.9 Hz), 3.92 (dd, 1H, J = 4.0, 8.9 Hz), 4.15 (t, 1H, J = 7.5 Hz), 5.18 (s, 1H), 7.25–7.30 (m, 2H), 7.31–7.40 (m, 8H); ^{13}C NMR (150 MHz, D_2O) δ 140.3 (× 2), 129.1 (× 4), 128.0 (× 2), 127.1 (× 2), 127.0 (× 2), 75.9, 70.6, 65.8, 60.9, 59.1, 58.4, 44.1, 29.3; HRMS calcd. for $[C_{20}H_{26}N_2O_3 + H]^+$ 343.2016, found 343.2017.

(2*R*,3*R*,4*S*,5*R*)-2-(2-aminoethyl)-5-(hydroxymethyl)pyrrolidine-3,4-diol (13)

Compound **12** (35 mg, 0.10 mmol) was dissolved in MeOH (0.2 mL), treated with $Pd(OH)_2/C$ and stirred at room temperature for 12 h under hydrogen atmosphere. The mixture was filtered, concentrated, and purified by CC to give **13** (18 mg, 0.08 mmol, 84%) as yellowish oil. TLC: $R_f = 0.3$ (n-propanol/$NH_{3(aq)}$, 3/1, v/v). $[\alpha]_D^{23} = +43.3$ (c = 0.1, H_2O). ^1H NMR (600 MHz, D_2O) δ 1.53–1.59 (m, 1H), 1.70–1.76 (m, 1H), 2.68–2.73 (m, 1H), 2.76–2.81 (m, 1H), 2.90 (td, 1H, J = 5.2, 8.4 Hz), 3.20 (dd, 1H, J = 6.6, 10.5 Hz), 3.48 (dd, 1H, J = 6.6, 11.0 Hz), 3.65 (dd, 1H, J = 7.0, 11.0 Hz), 3.73 (dd, 1H, J = 4.2, 8.4 Hz), 4.05 (t, 1H, J = 4.2 Hz); ^{13}C NMR (150 MHz, D_2O) δ 77.5, 71.9, 60.5, 59.2, 57.6, 38.0 34.2; HRMS calcd. for $[C_7H_{16}N_2O_3 + H]^+$ 177.1234, found 177.1236.

Synthesis of compounds 4 and 16

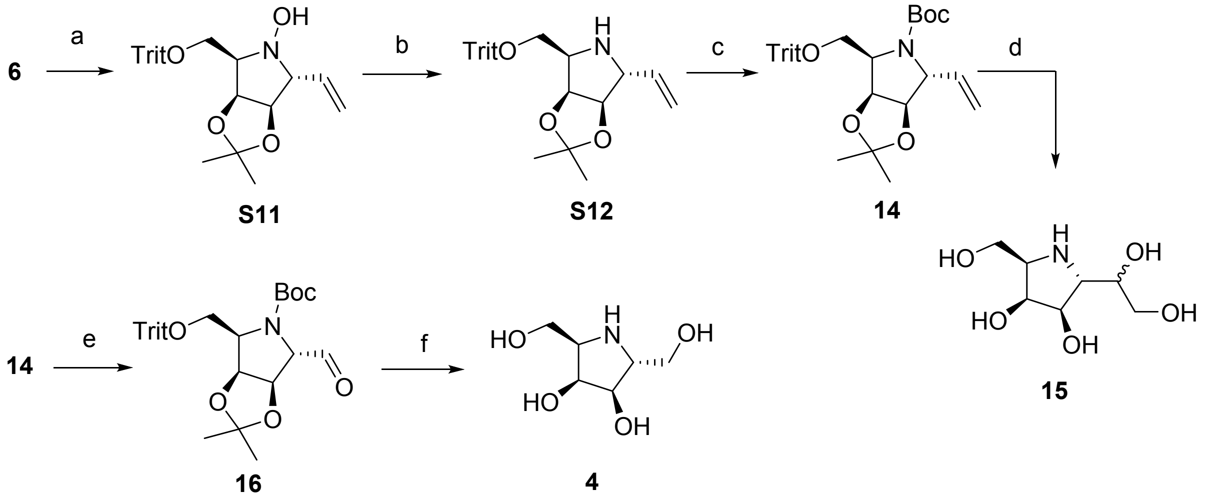

Scheme S4. Synthesis of compounds **4** and **15**. Reagents and conditions: a) vinylMgBr, THF, 0 °C, 2 h, 95%; b) Zn, AcOH, CH_2Cl_2, rt, 12 h, 67%; c) Boc_2O, Et_3N, MeOH, rt, 12 h, 95%; d) 1. OsO_4, *N*-methylmorpholine *N*-oxide, THF/H_2O, rt, 12 h; 2. 6N HCl, MeOH, rt, 12 h; then DOWEX (OH^-), 74% over two steps. e) O_3, CH_2Cl_2, -78 °C, 0.5 h, then Me_2S, 84%; f) 1. $NaBH_4$, MeOH, rt, 12 h; 2. 6N HCl, MeOH, rt, 12 h; then DOWEX (OH^-), 76% over two steps.

(3a*S*,4*R*,6*R*,6a*R*)-2,2-Dimethyl-4-((trityloxy)methyl)-6-vinyltetrahydro-5*H*-[1,3]dioxolo[4,5-c]pyrrol-5-ol (S11)

Cyclic nitrone **6** (100 mg, 0.23 mmol) was dissolved in dry THF (2 mL), and then vinyl magnesium bromide (0.4 mL, 0.69 mmol) was added dropwise at 0 °C. After 1 h, the reaction mixture was quenched with $NH_4Cl_{(aq)}$, extracted with EtOAc, dried with $MgSO_4$, concentrated, and purified by CC to give **S11** (101 mg, 0.22 mmol, 94%) as a white solid. TLC: R_f = 0.3 (EtOAc/n-hexanes, 1/3, v/v). ^1H NMR (600 MHz, $CDCl_3$) δ 1.27 (s, 6H), 3.21–3.23 (q, 1H, J = 5.64 Hz), 3.24-3.38 (m, 1H), 3.52–3.55 (t, 1H, J = 8.22 Hz), 3.94–3.95 (d, 1H, J = 8.04 Hz), 4.50-4.51 (d, 1H, J = 6.6 Hz), 4.75–4.77 (t, 1H, J = 6.12 Hz), 5.24–5.30 (m, 2H), 5.85–5.91 (m, 1H), 7.19–7.48 (m, 15H).; ^{13}C NMR (150MHz, $CDCl_3$) δ 24.6, 25.7, 61.2, 67.0, 81.7, 86.9, 111.3, 119.8, 126.9–128.8 (× 18), 134.8, 144.0; HRMS calcd. for $[C_{29}H_{31}NO_4 + H]^+$ 458.2326, found 458.2326.

(3a*S*,4*R*,6*R*,6a*R*)-2,2-Dimethyl-4-((trityloxy)methyl)-6-vinyltetrahydro-4*H*-[1,3]dioxolo[4,5-c]pyrrole (S12)

Alkene **S11** (500 mg, 1.1 mmol) was treated with Zn (724 mg, 11 mmol) and AcOH (1.1 mL, 17.6 mmol) in CH_2Cl_2 (5 mL), and stirred at room temperature for 12h. After quenched with $NaHCO_{3(aq)}$, the mixture was extracted with CH_2Cl_2, dried with $MgSO_{4(s)}$, concentrated, and purified by CC to give amine **S12** (326 mg, 0.74 mmol, 67%) as a white solid. TLC: R_f =

0.2 (EtOAc/n-hexanes, 1/3, v/v). ^{1}H NMR (600 MHz, $CDCl_3$) δ 1.33 (s, 3H), 1.40 (s, 3H), 2.08 (s, 1H), 3.24 (dd, 1H, J = 4.2, 6.0 Hz), 3.29 (dd, 1H, J = 6.6, 8.4 Hz), 3.44 (dd, 1H, J = 6.6, 9.0 Hz), 3.71 (d, 1H, J = 5.4 Hz), 4.59 (d, 1H, J = 6.0 Hz), 4.71 (t, 1H, J = 4.8 Hz), 5.17 (d, 1H, J = 10.2 Hz), 5.28 (d, 1H, J = 17.4 Hz), 5.76–5.82 (m, 1H), 7.24–7.27 (m, 3H), 7.31–7.34 (m, 6H), 7.52–7.53 (m, 6H); ^{13}C NMR (150 MHz, $CDCl_3$) δ 144.1 (× 3), 136.5, 128.8 (× 6), 127.7 (× 6), 126.9 (× 3), 115.9, 111.1, 86.7, 85.6, 81.1, 65.0, 62.1, 60.7, 26.0, 24.4; HRMS calcd. for $[C_{29}H_{31}NO_3 + H]^+$ 442.2377, found 442.2378.

tert-Butyl(3a_S_,4_R_,6_R_,6a_R_)-2,2-dimethyl-4-((trityloxy)methyl)-6-vinyltetrahydro-5_H_-[1,3]dioxolo[4,5-c]pyrrole-5-carboxylate (14)

Amine **S12** (100 mg, 0.23 mmol) was treated with Boc_2O (154 µL, 0.69 mmol) and Et_3N (96 µL, 0.69 mmol) in CH_2Cl_2 (2 mL), and was stirred at room temperature for 12h. The mixture was extracted with CH_2Cl_2, dried with $MgSO_{4(s)}$, concentrated, and purified by CC to give alkene **14** (123 mg, 0.22 mmol, 95%) as a colorless oil. TLC: R_f = 0.5 (EtOAc/n-hexanes, 1/5, v/v). ^{1}H NMR (600 MHz, $CDCl_3$) δ 1.30–1.41 (m, 15H), 3.51 (dd, 1H, J = 8.0, 8.3), 3.73–3.77 (m, 1H), 3.81–4.26 (m, 1H), 4.29–4.52 (m, 2H), 4.93 (s, 1H), 5.08 (d, 1H, J = 17.2 Hz), 5.14 (d, 1H, J = 10.2 Hz), 5.70 (s, 1H), 7.23–7.28 (m, 3H), 7.30–7.34 (m, 6H), 7.52–7.54 (m, 6H); ^{13}C NMR (150 MHz, $CDCl_3$) δ 154.5, 144.1 (× 3), 135.0, 128.9 (× 6), 127.6 (× 6), 126.9 (× 3), 115.5, 111.4, 86.8, 82.5, 80.0 (× 2), 66.7, 61.1, 60.3, 28.3 (× 3), 26.4, 25.3; HRMS calcd. for $[C_{34}H_{39}NO_5 + Na]^+$ 564.2720, found 564.2720.

(2_R_,3_R_,4_S_,5_R_)-2-((_S_)-1,2-Dihydroxyethyl)-5-(hydroxymethyl)pyrrolidine-3,4-diol (15)[17]

Alkene **14** (100 mg, 0.23 mmol) was dissolved in THF/H_2O (1 mL), before OsO_4 (trace) was added. _N_-methylmorpholine-_N_-oxide (32 mg, 0.28 mmol) was added and the mixture was stirred for 6 h at rt. After the reaction was completed, $Na_2S_2O_{5(aq)}$ was added and the mixture was stirred vigorously for another 1 h. EtOAc and water were added and the layers were separated. The aqueous layer was extracted with EtOAc, and the combined extracts were washed with brine, dried over $MgSO_{4(s)}$, concentrated and purified by CC to afford dihydroxylated intermediate as a mixture of inseparable two diastereoisomers. The intermediate was dissolved in MeOH (1 mL) with 6N HCl, and stirred at room temperature for 12 h. The mixture was quenched with resin (DOWEX 550A), filtered, concentrated, and purified by CC to give compound **15** (136 mg, 0.7 mmol, 74% over two steps, a 1:1 mixture of two inseparable diastereoisomers) as a colorless oil. TLC: R_f = 0.6 ($CHCl_3$/MeOH/$NH_{3(aq)}$, 88/58/13.5, v/v). $[\alpha]_D^{23}$ = +101.7 (c = 0.1, H_2O). ^{1}H NMR (600 MHz, D_2O) δ 3.18–3.22 (m, 2H), 3.35–3.38 (m, 1H), 3.41–3.45 (m, 1H), 3.49–3.57 (m, 2H), 3.59–3.69 (m, 4H), 3.73–3.85 (m, 4H), 4.08–4.17 (m, 3H), 4.18–4.22 (m, 1H); ^{13}C NMR (150 MHz, $CDCl_3$) δ 72.9, 72.1, 71.6, 71.2, 70.9, 69.7, 63.4, 62.9, 61.6, 61.3, 61.0, 60.9, 59.2, 58.9; HRMS calcd. for $[C_7H_{15}NO_5 + H]^+$ 194.1023, found 194.1024.

***tert*-Butyl (3a*S*,4*R*,6*R*,6a*R*)-2,2-dimethyl-4-((trityloxy)methyl)-6-vinyltetrahydro-5H-[1,3]dioxolo[4,5-c]pyrrole-5-carboxylate (16)**

A solution of alkene **14** (1 g, 1.77 mmol) in CH_2Cl_2 (10 mL) at -78 °C was saturated with ozone until a blue color persisted. The solution was then purged with argon until disappearance of the blue coloration and then treated with Me_2S (650 μL, 8.85 mmol), concentrated, and purified by CC to give aldehyde **16** (0.85 g, 1.49 mmol, 84%) as a white solid. TLC: $R_f = 0.3$ (EtOAc/n-hexane, 1/5, v/v). ^{1}H NMR (600 MHz, $CDCl_3$) δ 1.03–1.58 (m, 15H), 3.45 (s, 1H), 3.72–4.11 (m, 2H), 4.17–4.42 (m, 1H), 4.60 (s, 1H), 4.81–5.05 (m, 1H), 7.08–7.36 (m, 9H), 7.48 (s, 6H), 9.54 (s, 1H); ^{13}C NMR (150 MHz, $CDCl_3$) δ 197.4, 154.8, 144.2 (× 3), 130.0 (× 6), 127.8 (× 6), 127.0 (× 3), 112.6, 86.9, 80.9, 79.6, 78.2, 72.0, 61.3, 60.8, 28.2 (× 3), 26.8, 25.4, HRMS calcd for $[C_{33}H_{37}NO_6 + Na]^+$ 566.2513, found 566.2512.

(2*R*,3*S*,4*R*,5*R*)-2,5-Bis(hydroxymethyl)pyrrolidine-3,4-diol (4)[18]

Aldehyde **16** (200 mg, 0.37 mmol) was dissolved in MeOH (2 mL), treated with $NaBH_4$ (42 mg, 1.11 mmol) at 0 °C, and then stirred for 1 h. After quenched with NH_4Cl, the mixture was extracted with EtOAc, dried with $MgSO_{4(s)}$, and concentrated. The residue was dissolved in MeOH with 6N HCl, and stirred for 12 h at room temperature. The mixture was quenched with resin (DOWEX 550A), filtered, concentrated, and purified by CC to give **4** (46 mg, 0.28 mmol, 76% over two steps) as colorless syrup. TLC: $R_f = 0.3$ (n-propanol/$NH_{3(aq)}$, 6/1, v/v). $[\alpha]_D^{23} = +103.4$ ($c = 0.1$, H_2O). ^{1}H NMR (600 MHz, D_2O) δ 3.63–3.68 (m, 1H), 3.76–3.80 (m, 1H), 3.85 (dd, 1H, J = 5.9, 12.6 Hz), 3.91 (dd, 1H, J = 8.1, 12.6 Hz), 3.95–4.02 (m, 2H), 4.28 (dd, 1H, J = 3.9, 9.2 Hz), 4.35 (dd, 1H, J = 3.4, 3.5 Hz); ^{13}C NMR (150 MHz, D_2O) δ 71.3, 70.2, 62.3, 61.8, 58.3, 57.6; HRMS calcd for $[C_6H_{13}NO_4 + H]^+$ 164.0917, found 164.0923.

Synthesis of compounds 5 and 17-19

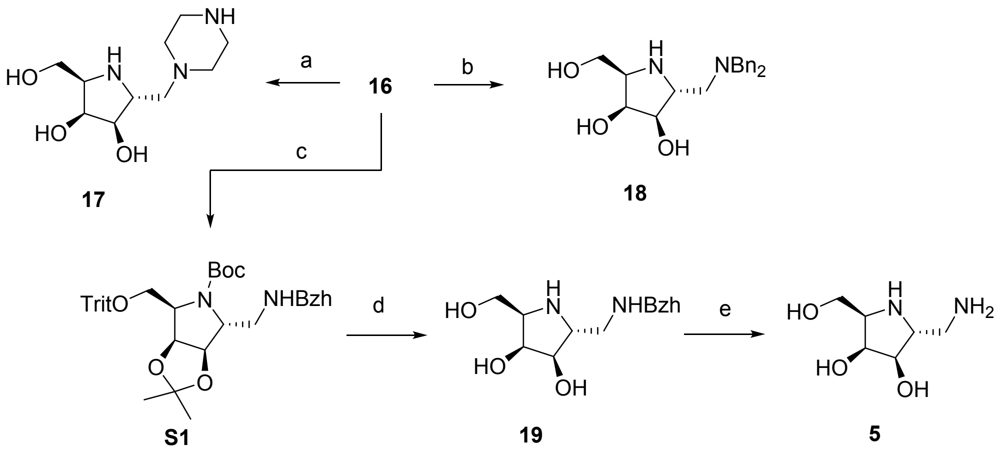

Scheme S5. Synthesis of compounds **5** and **17-19**. Reagents and conditions: a) 1. $NaBH_3CN$, 1-Boc-piperazine, AcOH, rt, 12 h; 2. 6N HCl, MeOH; then DOWEX (OH^-), rt, 4 h; then DOWEX (OH^-), 56 % over 2 steps; b) 1. $NaBH_3CN$, dibenzylamine, AcOH, MeOH, rt, 24 h; 2. 6N HCl, MeOH, rt, 12 h, 37% over two steps; c) $NaBH_3CN$, NH_2Bzh, AcOH, MeOH, rt, 12 h, 83%; d) 6N HCl, MeOH, rt, 12 h; then DOWEX (OH^-), 81%; e) H_2, $Pd(OH)_2/C$, MeOH, rt, 12 h, 75%.

(2*R*,3*S*,4*R*,5*R*)-2-(Hydroxymethyl)-5-(piperazin-1-ylmethyl)pyrrolidine-3,4-diol (17)

Aldehyde **16** (500 mg, 0.92 mmol) was treated with $NaBH_3CN$ (174 mg, 2.76 mmol), 1-Boc-piperazine (514 mg, 2.76 mmol), and AcOH (165 μL, 2.76 mmol) in MeOH (10 mL), and stirred at room temperature for 12 h. The mixture was quenched with 1N NaOH(aq), extracted, concentrated, and purified by CC to give a Boc-protected intermediate. A solution of the Boc-protected intermediate with 6N HCl in MeOH (5 mL) was stirred at room temperature for 12 h. The mixture was quenched with resin (DOWEX 550A), filtered, evaporated, and purified by CC to give **17** (120 mg, 0.52 mmol, 56% over two steps) as a yellow oil. TLC: Rf = 0.22 (2-propanol/$NH_{3(aq)}$, 2/1, v/v). $[\alpha]_D^{23}$ = +45.7 (*c* = 0.1, H_2O). ^1H NMR (600 MHz, D_2O) δ 2.56 (dd, 1H, *J* = 9.2, 13.2 Hz), 2.60-2.66 (m, 2H), 2.68 (dd, 1H, *J* = 3.3, 13.2 Hz), 2.70-2.75 (m, 2H), 3.05-3.12 (m, 4H), 3.23 (dt, 1H, *J* = 3.2, 8.9 Hz), 3.30 (dt, 1H, *J* = 3.7, 6.6 Hz), 3.59 (dd, 1H, *J* = 6.6, 11.1 Hz), 3.75 (dd,1H, *J* = 6.8, 11.1 Hz), 3.83 (dd, 1H, *J* = 4.2, 8.7 Hz), 4.12 (t, 1H, *J* = 4.0 Hz) ; ^{13}C NMR (150 MHz, D_2O) δ 75.9, 71.3, 61.5, 60.2, 59.6, 56.7, 50.5 (× 2), 43.2 (× 2). HRMS calcd. for $[C_{10}H_{21}N_3O_3 + H]^+$ 232.1656, found 232.1653.

(2*R*,3*R*,4*S*,5*R*)-2-((dibenzylamino)methyl)-5-(hydroxymethyl)pyrrolidine-3,4-diol (18)

Aldehyde **16** (500 mg, 0.92 mmol) was treated with $NaBH_3CN$ (174 mg, 2.76 mmol), diebenzylamine (531 μL, 2.76 mmol), and AcOH (165 μL, 2.76 mmol) in MeOH (10 mL), and stirred at room temperature for 12 h. The mixture was quenched with 1N NaOH(aq), extracted, concentrated, and purified by CC to give Boc-protected intermediate. A solution of Boc-

protected intermediate with 6N HCl in MeOH (5 mL) was stirred at room temperature for 12 h. The mixture was quenched with resin (DOWEX 550A), filtered, evaporated, and purified by CC to give **18** (116 mg, 0.34 mmol, 37% over two steps) as a white solid. TLC: R_f = 0.2 (CH_2Cl_2/MeOH, 4/1, v/v). $[\alpha]_D^{23}$ = +15.1 (*c* = 0.1, DMSO). ^{1}H NMR (600 MHz, D_2O) δ 2.71 (dd, 1H, *J* = 9.6, 13.8 Hz), 2.82 (dd, 1H, *J* = 4.2, 13.8 Hz), 3.21 (m,1H), 3.48 (m,1H), 3.63 (d, 2H, *J* = 13.2 Hz), 3.66 (dd, 1H, *J* = 7.2, 12 Hz), 3.77 (d, 2H, *J* = 13.2 Hz), 3.80 (dd, 1H, *J* = 6, 12 Hz), 3.84 (dd, 1H, *J* = 4.2, 7.8 Hz), 4.12 (t, 1H, *J* = 4.2 Hz), 7.35–7.38 (m, 2H), 7.387.44 (m, 8H); ^{13}C NMR (150 MHz, D_2O) δ137.8 (× 2), 129.7 (× 4), 128.7 (× 4), 127.7 (× 2), 74.8, 70.8, 60.4, 58.8, 58.0, 57.6 (× 2), 54.3. HRMS calcd. for $[C_{20}H_{26}N_2O_3 + H]^+$ 343.2016, found 343.2018.

***tert*-Butyl-(3a*R*,4*R*,6*R*,6a*S*)-4-((benzhydrylamino)methyl)-2,2-dimethyl-6-((trityloxy)methyl)tetrahydro-5*H*-[1,3]dioxolo[4,5-c]pyrrole-5-carboxylate (S1)**

Aldehyde **16** (500 mg, 0.92 mmol) was treated with $NaBH_3CN$ (173 mg, 2.76 mmol), benzhydrylamine (476 µL, 2.76 mmol), and AcOH (165 µL, 2.76 mmol) in MeOH (10 mL), and stirred at room temperature for 12 h. The mixture was quenched with 1N $NaOH_{(aq)}$, extracted, concentrated, and purified by CC to give compound **S1** (543 mg, 0.76 mmol, 83%) as a colorless oil. TLC: R_f = 0.5 (EtOAc/*n*-hexanes, 1/7, v/v). ^{1}H NMR (600 MHz, $CDCl_3$) *δ* 1.20–1.41 (m, 15H), 2.60–2.80 (m, 2H), 3.48 (t, 1H, *J* = 9.1 Hz), 3.75 (s, 1H), 3.80–4.10 (m, 2H), 4.64 (d, 1H, *J* = 5.6 Hz), 4.82 (s, 1H), 4.97 (t, 1H, *J* = 5.6 Hz), 7.24–7.27 (m, 5H), 7.30–7.36 (m, 10H), 7.41–7.43 (m, 4H), 7.53–7.55 (m, 6H); ^{13}C NMR (150 MHz, $CDCl_3$) *δ* 154.3, 144.4 (× 3), 143.9 (× 2), 128.9 (× 6), 128.6 (× 2), 128.5 (× 2), 127.7 (× 6), 127.3 (× 2), 127.2 (× 2), 127.1 (× 2), 126.9 (× 3), 111.2, 86.8, 81.8, 81.1, 80.2, 67.5, 64.6, 61.7, 61.1, 47.5, 28.3 (× 3), 26.5, 25.3; HRMS calcd. for $[C_{46}H_{50}N_2O_5 + H]^+$ 711.3792, found 711.3814.

(2*R*,3*R*,4*S*,5*R*)-2-((Benzhydrylamino)methyl)-5-(hydroxymethyl)pyrrolidine-3,4-diol (19)

Compound **S1** (500 mg, 0.70 mmol) was dissolved in MeOH (5 mL) with 6N HCl, and stirred for 12 h at room temperature. The mixture was quenched with resin (DOWEX 550A), filtered, concentrated, and purified by CC to give **19** (185 mg, 0.56 mmol, 81%) as a white solid. TLC: R_f = 0.5 ($CHCl_3$/MeOH/$NH_{3(aq)}$, 60/25/4, v/v). $[\alpha]_D^{23}$ = +10.2 (*c* = 0.1, DMSO). ^{1}H NMR (600 MHz, D_2O) δ 3.50 (dd, 1H, J = 4.1, 14.1 Hz), 3.58 (dd, 1H, J = 8.7, 14.1 Hz), 3.82–3.878 (m, 1H), 3.88–3.95 (m, 2H), 3.98 (dd, 1H, J = 4.6, 12.1 Hz), 4.18 (dd, 1H, J = 3.5, 9.6 Hz), 4.29-4.32 (m, 1H), 5.64 (s, 1H), 7.44–7.56 (m, 10H); ^{13}C NMR (150 MHz, D_2O) δ141.96, 141.91, 128.98 (× 2), 128.97 (× 2), 127.7 (× 2), 127.14 (× 2), 127.07 (× 2), 74.1, 70.5, 66.0, 61.3, 59.5, 58.2, 47.3. HRMS calcd. for $[C_{19}H_{24}N_2O_3 + H]^+$ 329.1860, found 329.1862.

(2*R*,3*R*,4*S*,5*R*)-2-(Aminomethyl)-5-(hydroxymethyl)pyrrolidine-3,4-diol (5)

The reaction was carried out as described for **13** starting from **19** to give **5** as a yellowish oil (75% yield). TLC: $R_f = 0.3$ (*n*-propanol/$NH_{3(aq)}$, 3/1, v/v). Analytical data were in agreement with literature.[12]

Synthesis of compounds 20-22 and S2

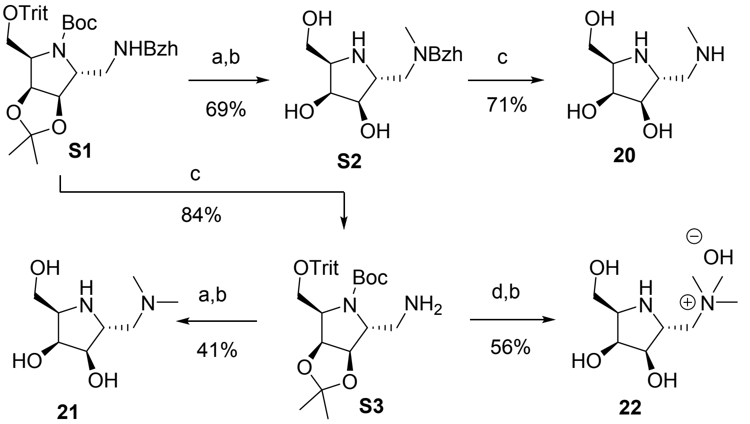

Scheme S6. Synthesis of compound **20-22** and **S2**. Reagents and conditions: a) $NaBH_3CN$, CH_2O, AcOH, MeOH, rt, 12 h. b) 6N HCl, MeOH, rt, 12 h; then DOWEX (OH^-). c) $Pd(OH)_2/C$, H_2, MeOH, rt, 12 h. d) MeI, MeOH, rt, 12 h.

(2*R*,3*R*,4*S*,5*R*)-2-((Benzhydryl(methyl)amino)methyl)-5-(hydroxymethyl) pyrrolidine-3,4-diol (S2)

Compound **S1** (200 mg, 0.28 mmol) was treated with formaldehyde (257 μL, 2.8 mmol), $NaBH_3CN$ (57 mg, 0.84 mmol), and AcOH (50 μL, 0.84 mmol) in MeOH (2 mL), and the reaction was stirred at room temperature for 12 h. The mixture was quenched with 1N $NaOH_{(aq)}$, extracted with EtOAc, dried with $MgSO_{4(s)}$, and concentrated. The residue was dissolved in MeOH (2 mL) with 6N HCl, and stirred for 12 h at room temperature. The mixture was quenched with resin (DOWEX 550A), filtered, concentrated, and purified by CC to give **S2** (65 mg, 0.19 mmol, 69% over two steps) as a white solid. TLC: $R_f = 0.3$ ($CHCl_3$/MeOH/$NH_{3(aq)}$, 60/25/4, v/v). $[\alpha]_D^{23} = +48.6$ ($c = 0.1$, DMSO). ^1H NMR (600 MHz, MeOD) δ 3.04 (s, 3H), 3.74 (s, 1H), 3.96–4.05 (m, 5H), 4.22 (s, 2H), 5.88 (s, 1H), 7.39–7.40 (m, 2H), 7.46–7.47 (m, 4H), 7.88–7.89 (m, 4H); ^{13}C NMR (150 MHz, MeOD) δ 135.2, 134.7, 129.5 (× 2), 129.4 (× 2), 129.3 (× 2), 128.3 (× 2), 128.2 (× 2), 75.9, 73.9, 68.8, 63.7, 57.7, 54.9, 54.2, 38.8; HRMS calcd. for $[C_{20}H_{26}N_2O_3 + H]^+$ 343.2016, found 343.2019.

(2*R*,3*S*,4*R*,5*R*)-2-(Hydroxymethyl)-5-((methylamino)methyl)pyrrolidine-3,4-diol (20)

Compound **S2** (35 mg, 0.10 mmol) was dissolved in MeOH (0.5 mL), treated with $Pd(OH)_2/C$ and stirred under hydrogen atmosphere at room temperature for 12 h. The mixture was filtered, concentrated, and purified by CC to give **20** (15 mg, 0.08 mmol, 84% over two steps) as yellowish oil. TLC: $R_f = 0.3$ (*n*-propanol/$NH_{3(aq)}$, 7/1, v/v). $[\alpha]_D^{23} = +41.6$ ($c = 0.1$,

H_2O). ^1H NMR (600 MHz, D_2O) δ 2.71 (s, 3H), 3.30 (dd, 1H, J = 7.8, 13.2 Hz), 3.33 (dd, 1H, J = 5.4, 13.2 Hz), 3.52–3.57 (m, 2H), 3.70 (dd, 1H, J = 7.7, 12 Hz), 3.82 (dd, 1H, J = 5.3, 12 Hz), 4.07 (dd, 1H, J = 3.6, 9.0 Hz), 4.16 (dd, 1H, J = 3.0, 3.6 Hz); ^{13}C NMR (150 MHz, D_2O) δ 74.8, 70.4, 61.7, 58.6, 56.3, 49.9, 33.4; HRMS calcd. for $[C_7H_{16}N_2O_3 + H]^+$ 177.1234, found 177.1233.

***tert*-Butyl(3a*R*,6*R*,6a*S*)-4-(aminomethyl)-2,2-dimethyl-6 ((trityloxy)methyl)tetrahydro-5H-[1,3]dioxolo[4,5-c]pyrrole-5-carboxylate (S3)**

Comound **S1** (35 mg, 0.10 mmol) was dissolved in MeOH (0.5 mL), treated with $Pd(OH)_2/C$, and stirred at room temperature for 12 h under hydrogen atmosphere. The mixture was filtered, concentrated, and purified by CC to give **S3** (18 mg, 0.08 mmol, 84%) as white solid. ^1H NMR (600 MHz, $CDCl_3$) δ 1.06–1.38 (m, 15H), 2.90 (m, 2H), 3.48 (d, J = 11.8 Hz, 2H), 3.79–3.86 (m, 2H), 4.55 (d, J = 5.8 Hz, 1H), 4.94 (d, J = 5.8 Hz, 1H), 7.23–7.26 (m, 3H), 7.29–7.32 (m, 6H), 7.51–7.52 (m, 6H); ^{13}C NMR (150 MHz, $CDCl_3$) δ 154.3, 144.3 (× 3), 129.0 (× 6), 127.6 (× 6), 126.9 (× 3), 111.5, 86.8, 80.7, 80.3, 79.9, 66.2, 61.5, 60.7, 41.5, 28.3 (× 3), 26.6, 25.4. HRMS calcd for $[C_{33}H_{40}N_2O_5 + H]^+$ 545.3024, found 545.3010.

(2*R*,3*R*,4*S*,5*R*)-2-((Dimethylamino)methyl)-5-(hydroxymethyl)pyrrolidine-3,4-diol (21)

Compound **S3** (200 mg, 0.28 mmol) was treated with formaldehyde (275 µL, 3.0 mmol), $NaBH_3CN$ (61 mg, 0.9 mmol), and AcOH (53 µL, 0.9 mmol) in MeOH (2 mL), and the reaction was stirred at room temperature for 12 h. The mixture was quenched with 1N $NaOH_{(aq)}$, extracted with CH_2Cl_2, dried with $MgSO_{4(s)}$, and concentrated. The residue was dissolved in MeOH (1 mL) with 6N HCl, and stirred for 12 h at room temperature. The mixture was quenched with resin (DOWEX 550A), filtered, concentrated, and purified by CC to give **21** (22 mg, 0.12 mmol, 41% over two steps) as a white solid. TLC: R_f = 0.6 ($CHCl_3$/MeOH/$NH_{3(aq)}$, 60/25/4, v/v). $[\alpha]_D^{23}$ = +20.25 (c = 0.1, DMSO). ^1H NMR (600 MHz, MeOD) δ 2.33 (s, 6H), 2.48–2.55 (m, 2H), 3.27–2.33 (m, 2H), 3.70 (dd, 1H, J = 6.5, 11.0), 3.76 (dd, 1H, J = 4.3, 7.7 Hz), 3.81 (dd, 1H, J = 5.9, 11.0 Hz), 3.93 (t, 1H, J = 8.3 Hz); ^{13}C NMR (150 MHz, MeOD) δ 76.2, 71.7, 62.8, 60.5, 60.4, 58.4, 44.4 (× 2); HRMS calcd. for $[C_8H_{18}N_2O_3 + H]^+$ 191.1390, found 191.1390.

1-((2*R*,3*R*,4*S*,5*R*)-3,4-Dihydroxy-5-(hydroxymethyl)pyrrolidin-2-yl)-*N*,*N*,*N*-trimethylmethanaminium hydroxide (22)

Compound **S3** (200 mg, 0.28 mmol) was treated with MeI (142 mg, 1 mmol) in MeOH (5 mL), and the reaction was stirred at room temperature for 12 h. The mixture was extracted with CH_2Cl_2, dried with $MgSO_{4(s)}$, and concentrated. The residue was dissolved in MeOH (1 mL) with 6N HCl, and stirred for 12 h at room temperature. The mixture was quenched with (DOWEX 550A), filtered, concentrated, and purified by CC to give **22** (35 mg, 0.16 mmol,

56% over three steps) as a white solid. TLC: R_f = 0.1 (*n*-propanol/$NH_{3(aq)}$, 1/1, v/v). $[\alpha]_D^{23}$ = +31.44 (*c* = 0.1, H_2O). ^1H NMR (600 MHz, D_2O) δ 3.01 (s, 9H), 3.21 (td, 1H, *J* = 3.5, 6.5), 3.39 (dd, 1H, *J* = 8.7, 13.4), 3.45–3.51 (m, 2H), 3.55 (dd, 1H, *J* = 6.5, 11.2 Hz), 3.70 (dd, 1H, *J* = 6.5, 11.2 Hz), 3.85 (dd, 1H, *J* = 3.7, 8.7 Hz), 4.04 (t, 1H, *J* = 3.7 Hz); ^{13}C NMR (150 MHz, D_2O) δ 75.8, 70.6 (× 2), 60.2, 60.0, 55.1, 53.6 (× 3); HRMS calcd. for $[C_9H_{21}N_2O_3]^+$ 205.1547, found 205.1549.

Synthesis of compounds S4-S6, 23 and 24

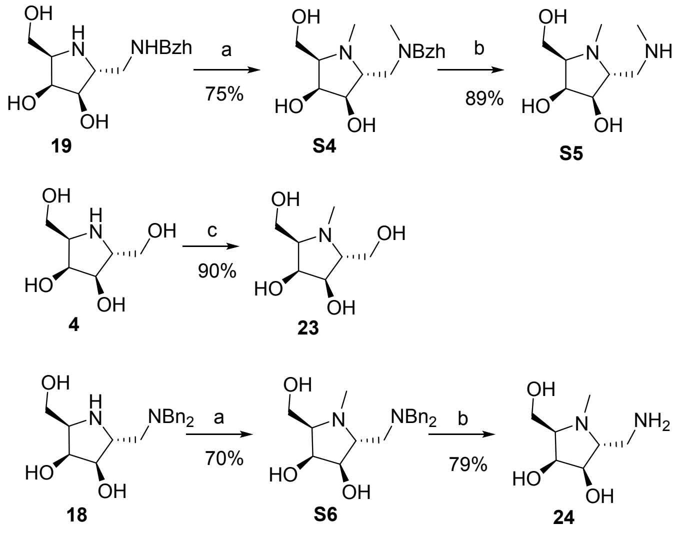

Scheme S7. Synthesis of compounds **S4-S6**, **23** and **24.** Reagents and conditions: a) $NaBH_3CN$, CH_2O, AcOH, MeOH, rt, 12 h. b) $Pd(OH)_2/C$, H_2, MeOH, rt, 12 h. c) H_2, $Pd(OH)_2/C$, CH_2O, MeOH, rt, 12 h.

(2*R*,3*R*,4*S*,5*R*)-2-((Benzhydryl(methyl)amino)methyl)-5-(hydroxymethyl)-1-methylpyrrolidine-3,4-diol (S4)

Compound **19** (100 mg, 0.3 mmol) was treated with formaldehyde (275 μL, 3.0 mmol), $NaBH_3CN$ (61 mg, 0.9 mmol), and AcOH (53 μL, 0.9 mmol) in MeOH (2 mL), and the reaction was stirred at room temperature for 12 h. The mixture was quenched with 1N $NaOH_{(aq)}$, extracted with CH_2Cl_2, dried with $MgSO_{4(s)}$, concentrated and purified by CC to give **S4** (80 mg, 0.2 mmol, 75%) as a white solid. TLC: R_f = 0.3 (CH_2Cl_2/MeOH, 4/1, v/v). $[\alpha]_D^{23}$ = -48.6 (*c* = 0.1, DMSO). ^1H NMR (600 MHz, $CDCl_3$) δ 2.26 (s, 3H), 2.33 (dd, 1H, *J* = 10.7, 11.8 Hz), 2.37 (s, 3H), 2.42 (dd, 1H, *J* = 4.1, 11.8 Hz), 2.83–2.86 (m, 1H), 3.24–3.26 (m, 1H), 3.76–3.79 (m, 2H), 4.12 (dd, 1H, *J* = 1.8, 5.1 Hz), 4.19 (dd, 1H, *J* = 5.7, 7.1 Hz), 4.31 (s, 1H), 7.20–7.23 (m, 2H), 7.28–7.32 (m, 4H), 7.41–7.43 (m, 4H); ^{13}C NMR (150 MHz, $CDCl_3$) δ 142.5 (× 2), 128.7 (× 2), 128.6 (× 2), 127.9 (× 2), 127.8 (× 2), 127.3, 127.2, 77.1, 74.7, 72.3, 65.2, 64.4, 59.0, 54.3, 41.6, 34.9; HRMS calcd. for $[C_{21}H_{28}N_2O_3 + H]^+$ 357.2173, found 357.2176.

(2*R*,3*S*,4*R*,5*R*)-2-(Hydroxymethyl)-1-methyl-5-((methylamino)methyl) pyrrolidine-3,4-diol (S5)

The reaction was carried out as described for **20** starting from **S4** to give **S5** as a colorless oil (89% yield). TLC: R_f = 0.4 ($CHCl_3$/MeOH/$NH_{3(aq)}$, 60/25/4, v/v). $[\alpha]_D^{23}$ = +57.8 (*c* = 0.1, H_2O). ^{1}H NMR (600 MHz, D_2O) δ 2.74 (s, 3H), 2.77 (s, 3H), 3.35–3.39 (m, 1H), 3.50–3.57 (m, 3H,), 3.88–3.95 (m, 2H), 4.22–4.25 (m, 2H); ^{13}C NMR (150 MHz, D_2O) δ 73.4, 70.1, 67.8, 64.8, 56.6, 47.8, 37.2, 34.0; HRMS calcd. for $[C_8H_{18}N_2O_3 + H]^+$ 191.1390, found 191.1389.

(2*R*,3*S*,4*R*,5*R*)-2,5-Bis(hydroxymethyl)-1-methylpyrrolidine-3,4-diol (23)[17]

Amine **4** (20 mg, 0.12 mmol) was treated with formaldehyde (110 μL, 1.2 mmol) and $Pd(OH)_2$/C in MeOH (1 mL) under hydrogen atmosphere and the reaction was stirred at room temperature for 12 h. The mixture was concentrated and purified by CC to afford compound **23** (18 mg, 0.1 mmol, 85%) as a white solid. TLC: R_f = 0.2 (NPA/$NH3_{(aq)}$, 8/1, v/v). $[\alpha]_D^{23}$ = +34.8 (*c* = 0.1, H_2O). ^{1}H NMR (600 MHz, D_2O) δ 2.94 (s, 3H), 3.46–3.49 (m, 1H), 3.60–3.63 (m, 1H), 3.84 (dd, 1H, *J* = 5.6, 13.2 Hz), 3.92–3.96 (m, 3H), 4.20 (dd, 1H, *J* = 4.2, 8.5 Hz), 4.27 (dd, 1H, *J* = 3.8, 4.2 Hz); ^{13}C NMR (150 MHz, D_2O) δ 70.9, 70.3 (× 2), 68.6, 57.0, 56.1, 37.9; HRMS calcd. for $[C_7H_{15}NO_4 + H]^+$ 178.1074, found 178.1073.

(2*R*,3*R*,4*S*,5*R*)-2-((Dibenzylamino)methyl)-5-(hydroxymethyl)-1-methyl pyrrolidine-3,4-diol (S6)

A solution of **18** (100 mg, 0.29 mmol), formaldehyde (686 μL, 8.7 mmol, 37% water solution) and AcOH (52 μL, 0.87 mmol) in MeOH (1 mL) was stirred at rt for 15 min. The mixture was treated with $NaBH_3CN$ (55 mg, 0.84 mmol) and stirred at rt. After 24 h, the mixture was neutralized with 1N $NaOH_{(aq)}$, concentrated and purified by CC to give **S6** (64 mg, 0.18 mmol, 70 %) as a white solid. TLC: R_f = 0.5 (CH_2Cl_2/MeOH, 4/1, v/v). $[\alpha]_D^{23}$ = –15.1 (*c* = 0.1, DMSO). ^{1}H NMR (600 MHz, MeOD) δ 2.30 (dd, 1H, *J* = 9.1, 12.9 Hz), 2.43 (s, 3H), δ 2.56 (dd, 1H, *J* = 4.5, 12.9 Hz), 2.86 (td, 1H, *J* = 3.4, 6.8 Hz), 3.06–3.11 (m, 1H), 3.44 (d, 2H, *J* = 13.3 Hz), 3.68 (dd, 1H, *J* = 3.2, 11.7 Hz), 3.69 (d, 2H, *J* = 13.2 Hz), 3.79 (dd, 1H, *J* = 4.0, 11.8 Hz), 3.85 (dd, 1H, *J* = 2.7, 5.7 Hz), 3.90 (t, 1H, *J* = 6.3 Hz), 7.23 (t, 4H, *J* = 7.3 Hz), 7.31 (t, 4H, J = 7.6 Hz), 7.38 (d, 2H, *J* = 7.3 Hz); ^{13}C NMR (150 MHz, MeOD) δ139.2 (× 2), 128.9 (× 4), 127.9 (× 4), 126.7 (× 2), 74.2, 70.6, 68.6, 66.2, 58.5 (× 2), 57.0, 53.7, 35.0. HRMS calcd. for $[C_{21}H_{28}N_2O_3 + H]^+$ 357.2173, found 357.2173

(2*R*,3*R*,4*S*,5*R*)-2-(Aminomethyl)-5-(hydroxymethyl)pyrrolidine-3,4-diol (24)

Compound **S6** (35 mg, 0.10 mmol) was dissolved in MeOH, treated with $Pd(OH)_2$/C, and stirred under a hydrogen atmosphere at room temperature for 24 h. The mixture was filtered, concentrated, and purified by CC to give **24** (14 mg, 0.08 mmol, 79%) as a yellowish solid. TLC: R_f = 0.3 (*n*-propanol/$NH_{3(aq)}$, 9/1, v/v). $[\alpha]_D^{23}$ = +13.9 (*c* = 0.1, H_2O). ^{1}H NMR (600

MHz, D_2O) δ 2.88 (s, 3H), 3.36 (dd, 1H, J = 8.4, 13.4 Hz), 3.54 (dd, 1H, J = 5.3, 13.4 Hz), 3.59 (s, 1H), 3.67 (s, 1H), 3.95 (d, 2H, J = 6.1 Hz), 4.25–4.27 (m, 2H); ^{13}C NMR (150 MHz, D_2O) δ 73.5. 70.5. 66.3 (× 2), 56.8, 38.8, 36.2; HRMS calcd. for $[C_7H_{16}N_2O_3 + H]^+$ 177.1240, found 177.1234.

4. NMR spectra

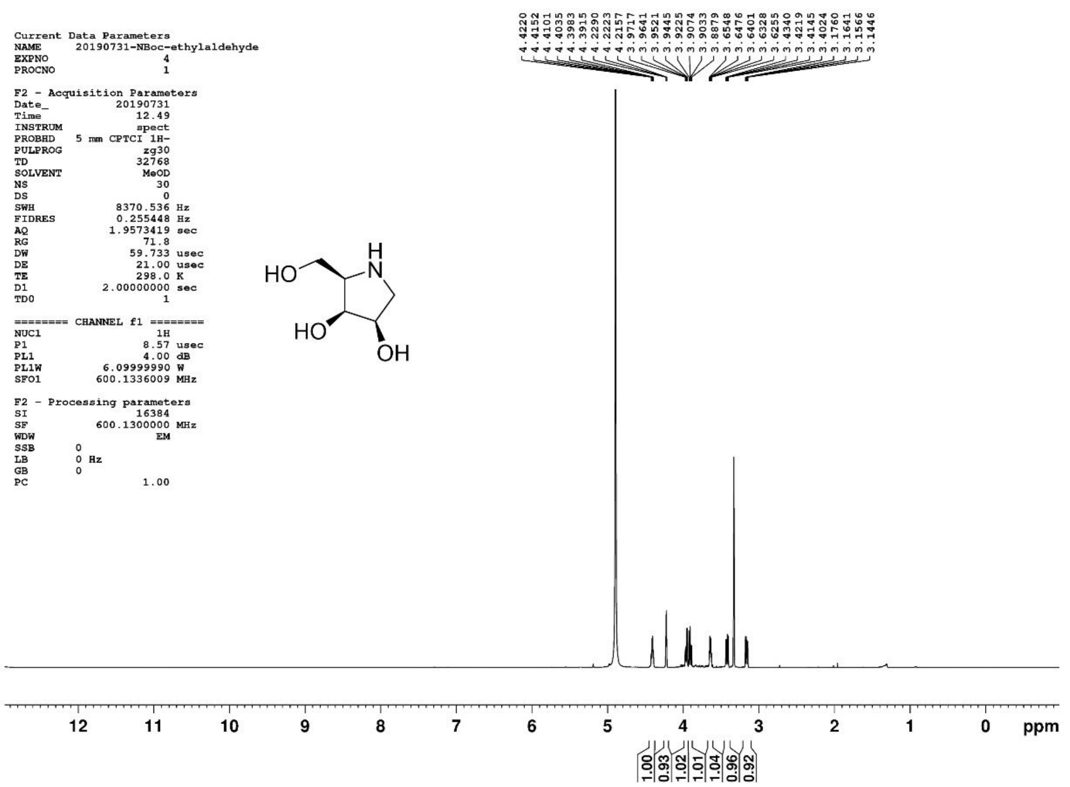

^{1}H spectrum of compound **7** (600 MHz, MeOD)

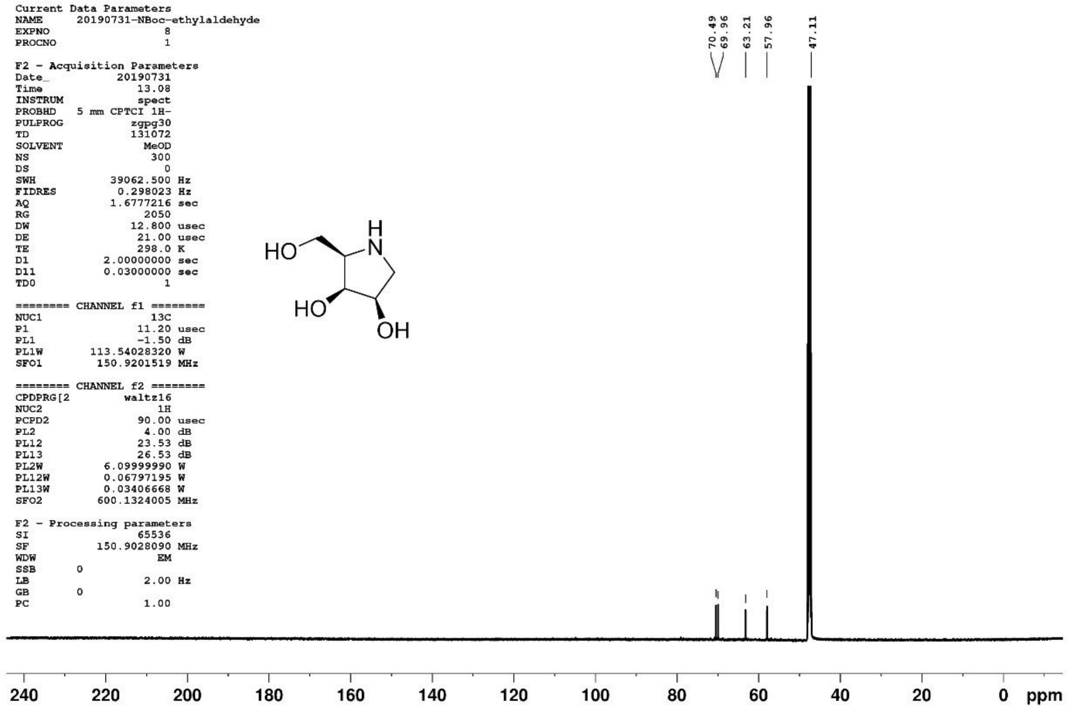

^{13}C spectrum of compound **7** (150 MHz, MeOD)

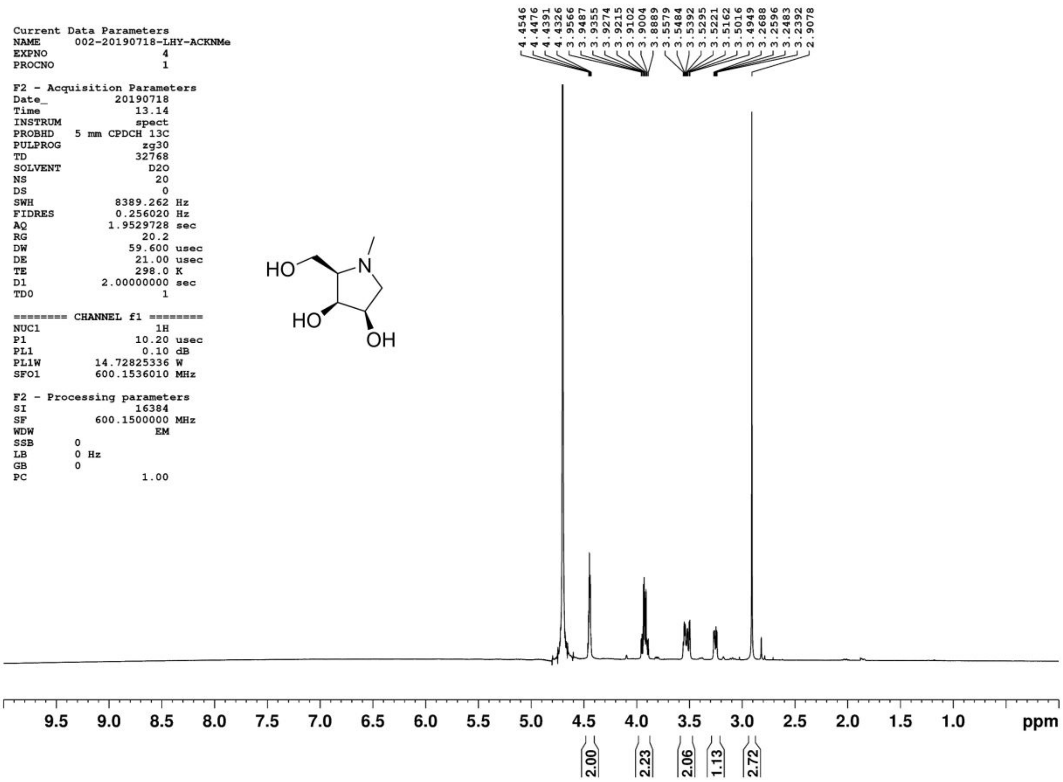

^{1}H spectrum of compound **8 (**600 MHz, D_2O)

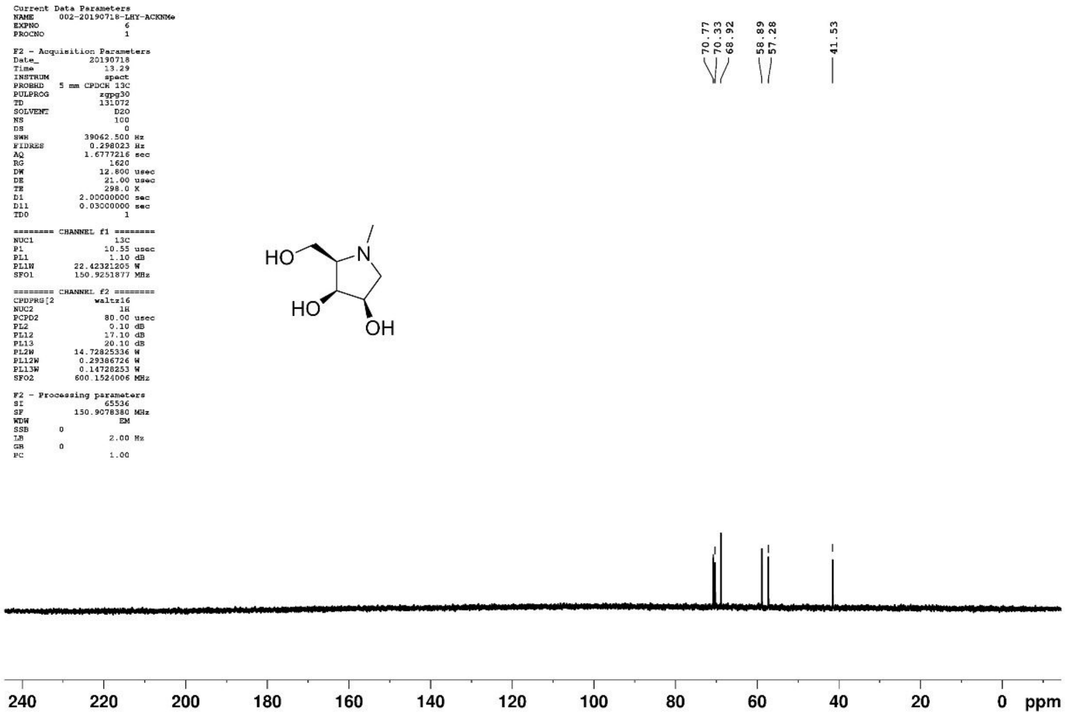

^{13}C spectrum of compound **8 (**150 MHz, D_2O)

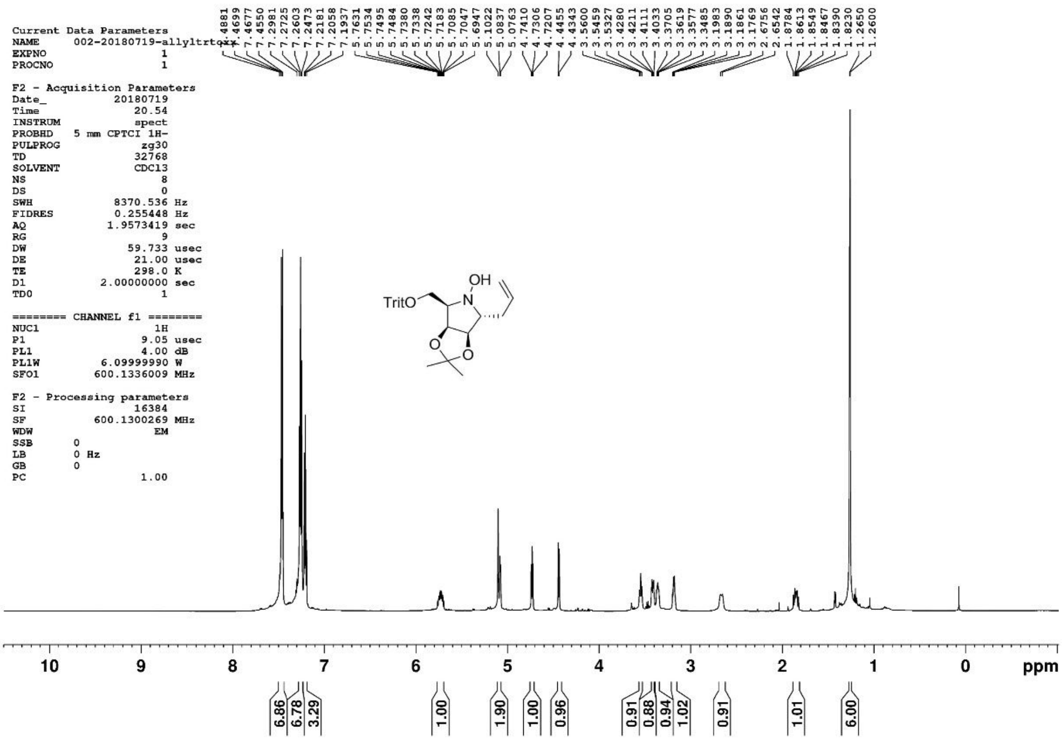

^{1}H spectrum of compound **S7 (**600 MHz, $CDCl_3$)

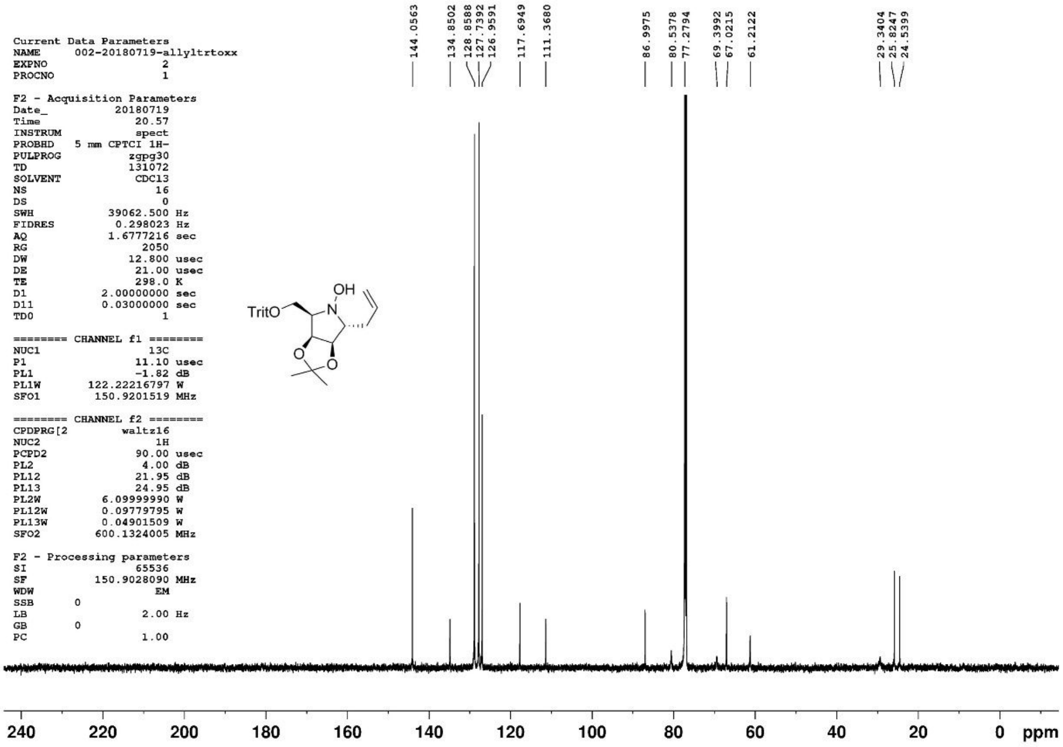

^{13}C spectrum of compound **S7 (**150 MHz, $CDCl_3$)

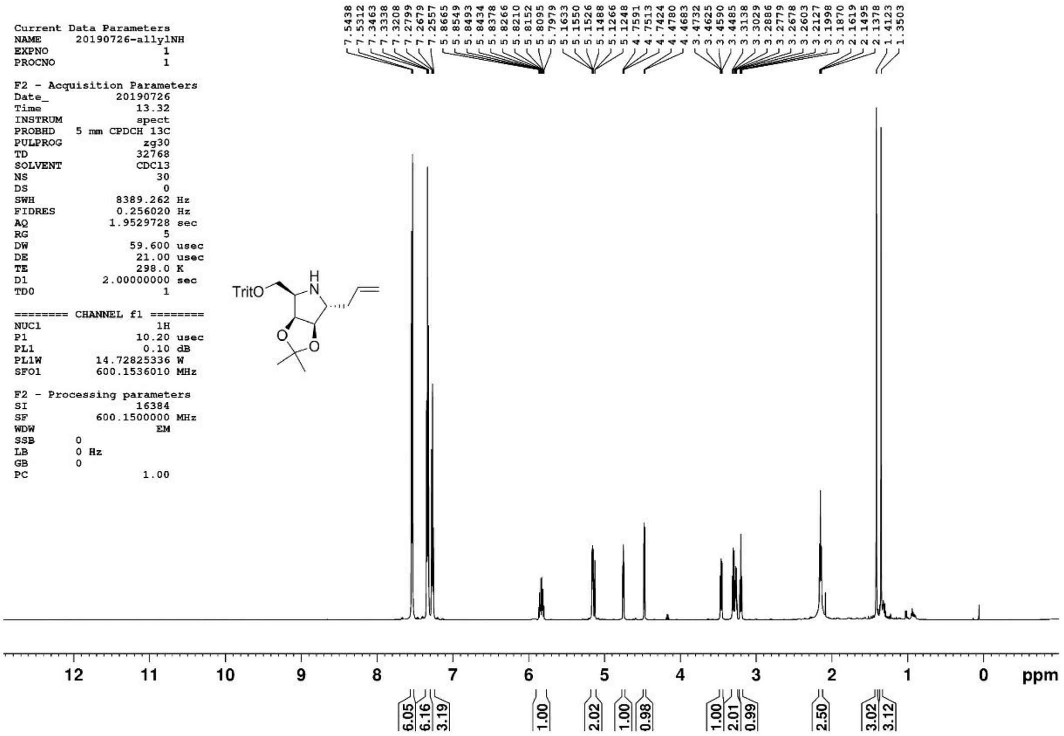

^{1}H spectrum of compound **S8 (**600 MHz, $CDCl_3$)

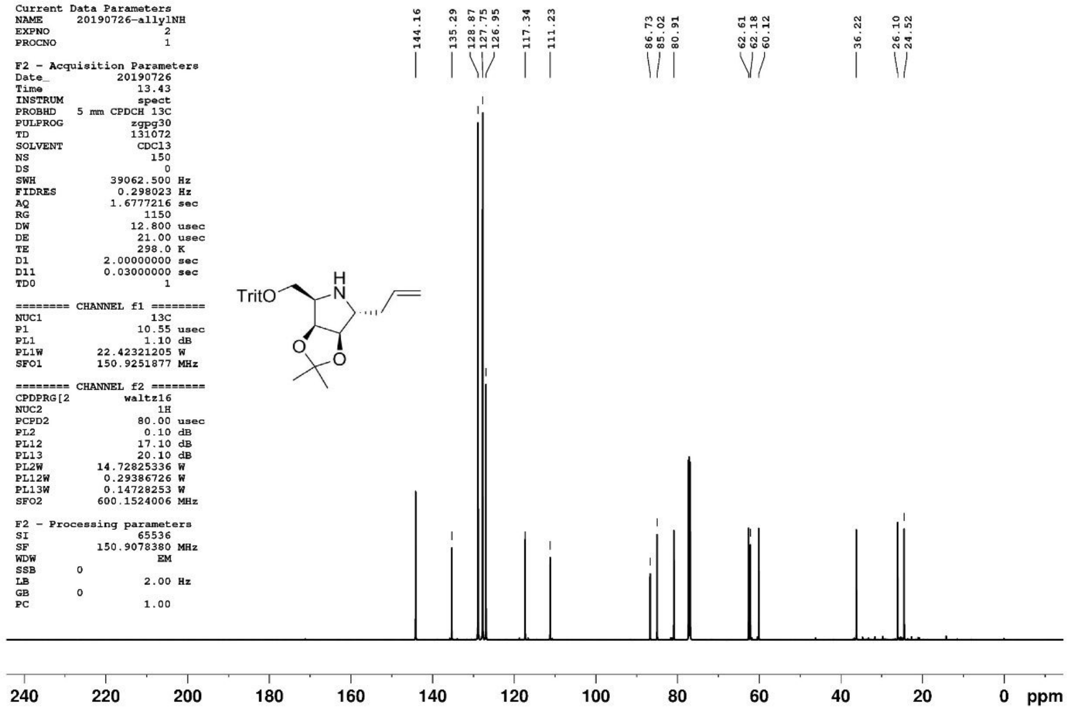

^{13}C spectrum of compound **S8 (**150 MHz, $CDCl_3$)

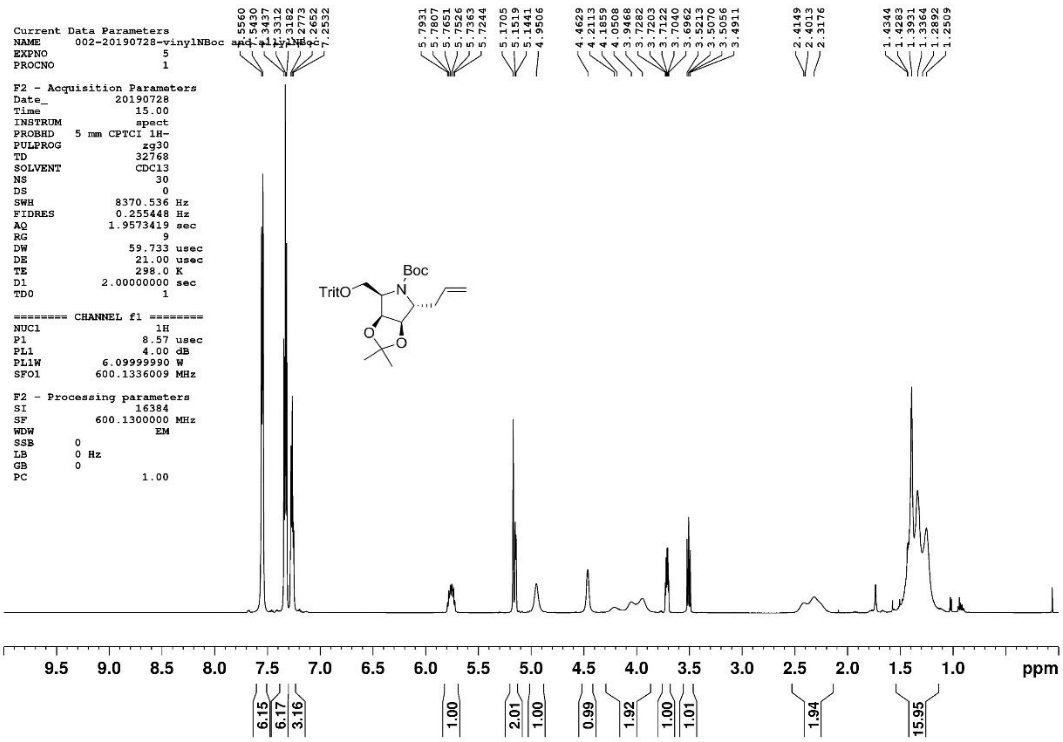

^{1}H spectrum of compound **9** **(**600 MHz, $CDCl_3$)

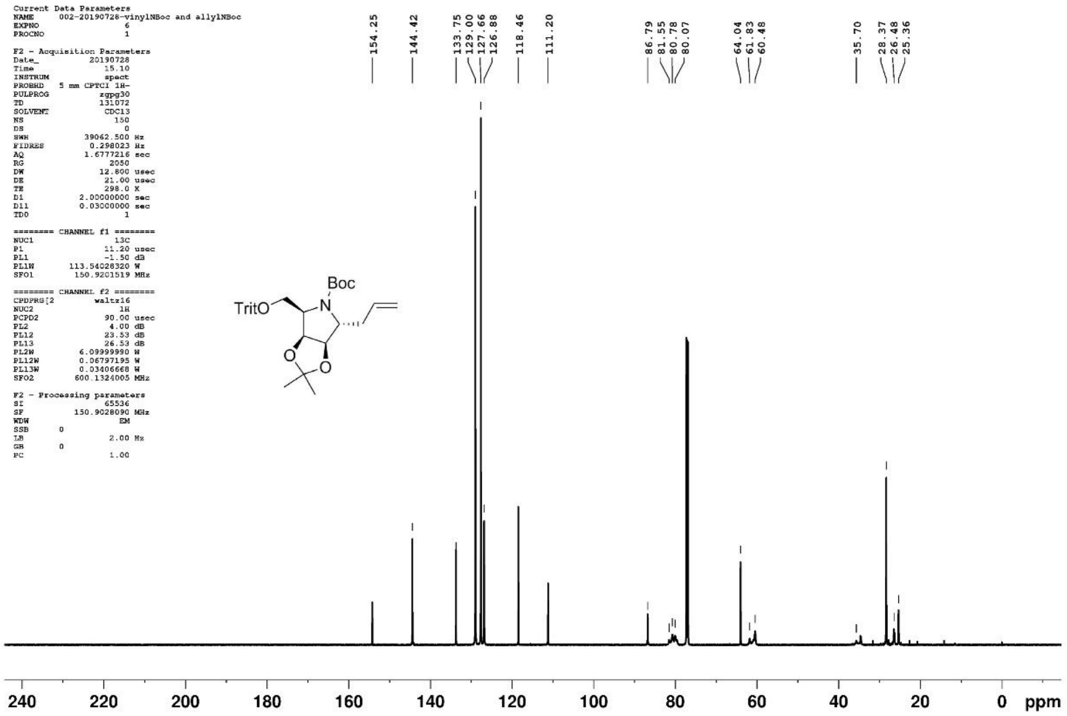

^{13}C spectrum of compound **9** **(**150 MHz, $CDCl_3$)

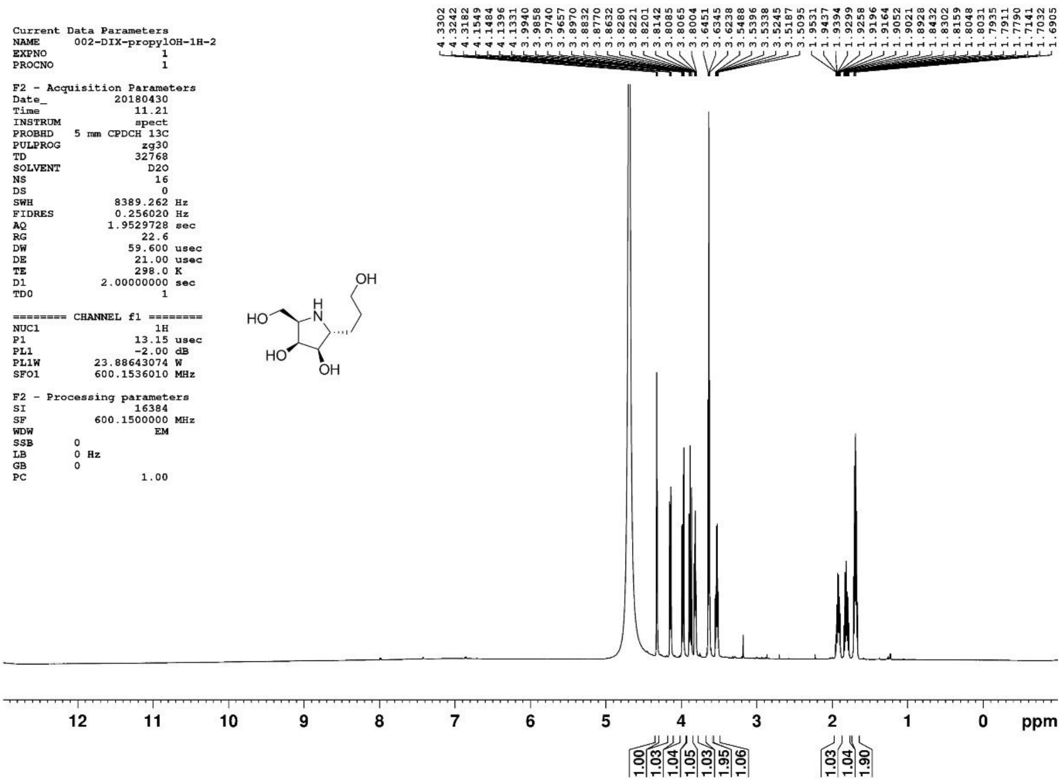

^{1}H spectrum of compound **10 (**600 MHz, D_2O)

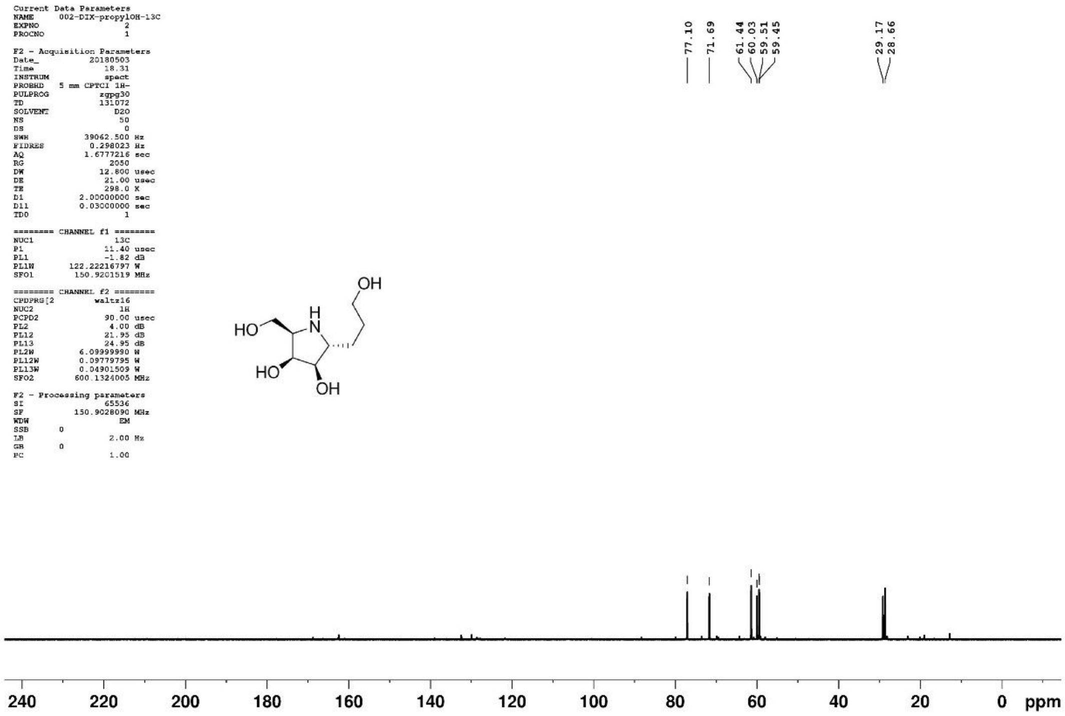

^{13}C spectrum of compound **10 (**150 MHz, D_2O)

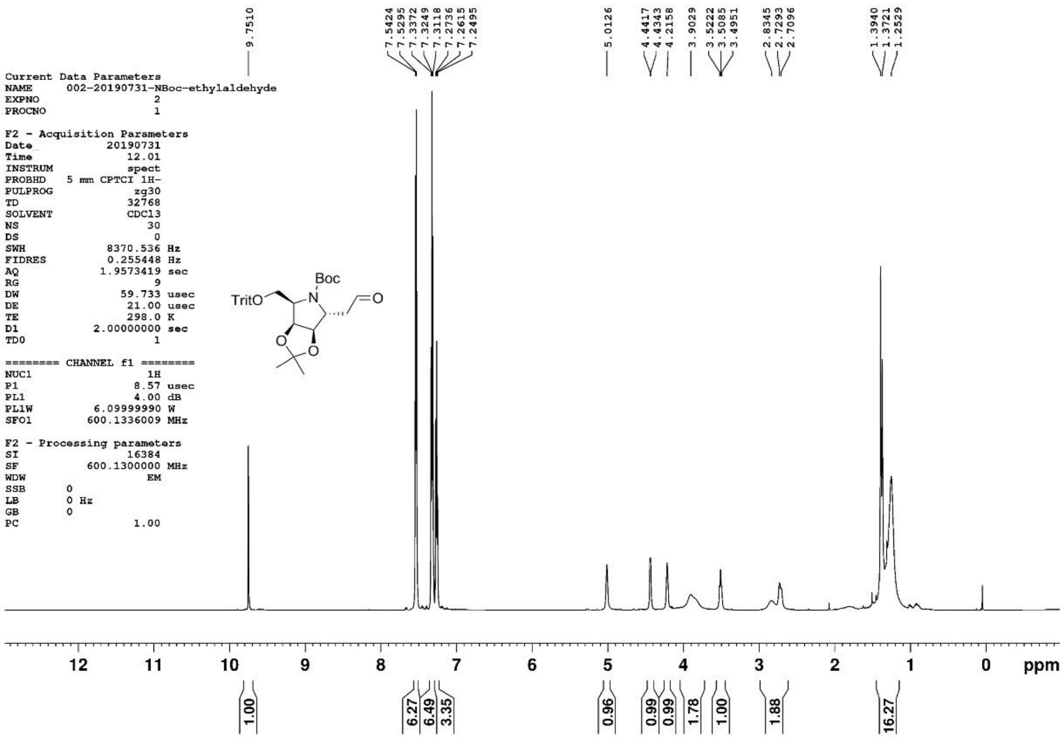

^{1}H spectrum of compound **S9 (**600 MHz, $CDCl_3$)

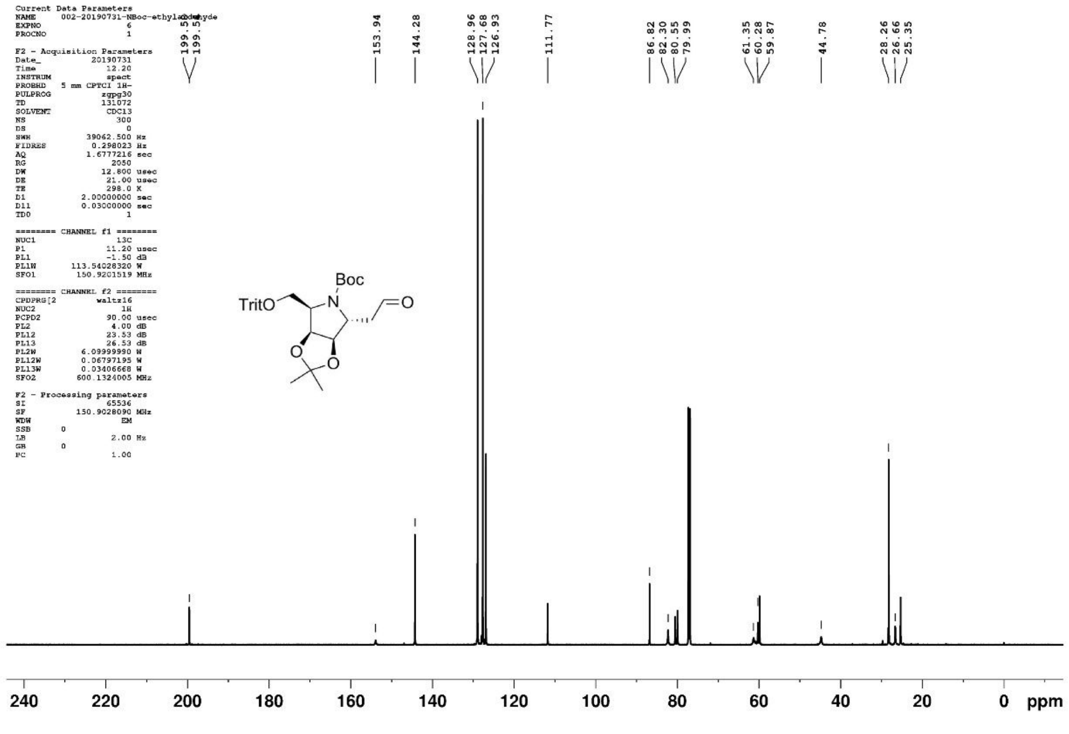

^{13}C spectrum of compound **S9 (**150 MHz, $CDCl_3$)

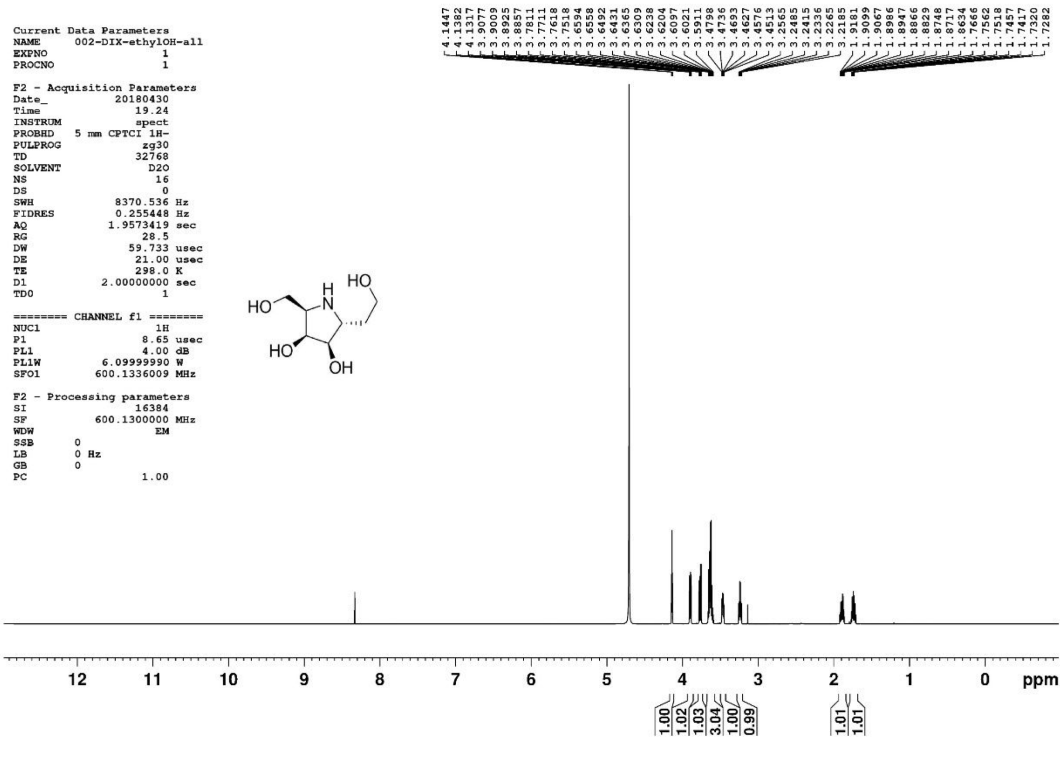

^{1}H spectrum of compound **11 (**600 MHz, D_2O)

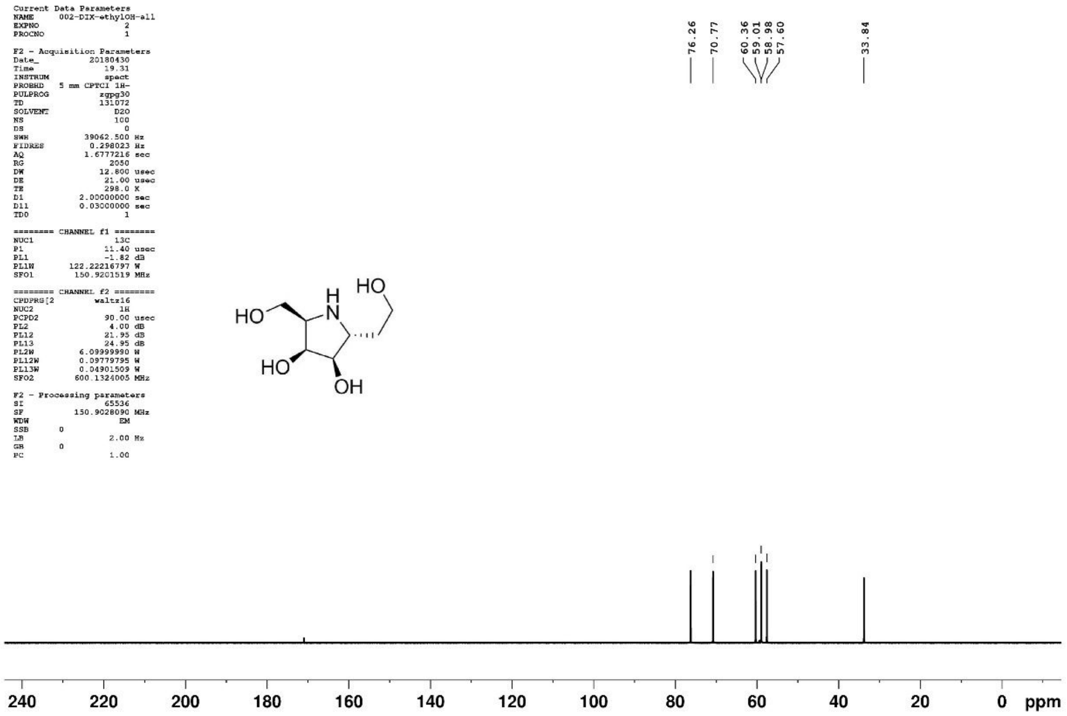

^{13}C spectrum of compound **11 (**150 MHz, D_2O)

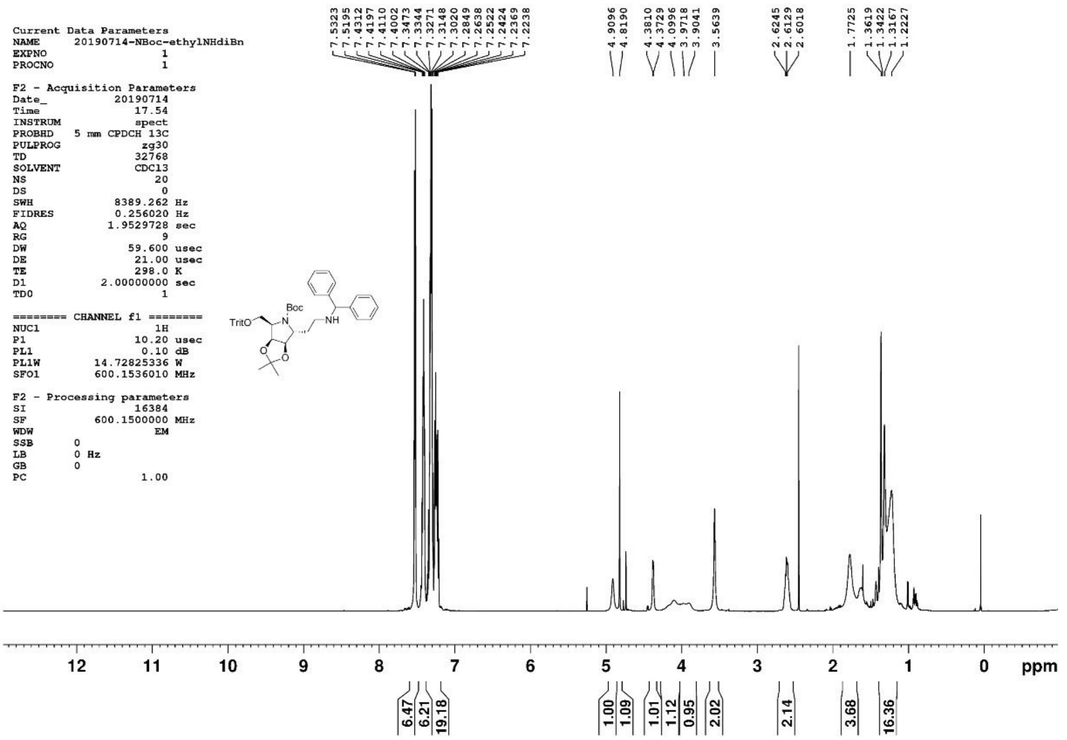

^{1}H spectrum of compound **S10 (**600 MHz, $CDCl_3$)

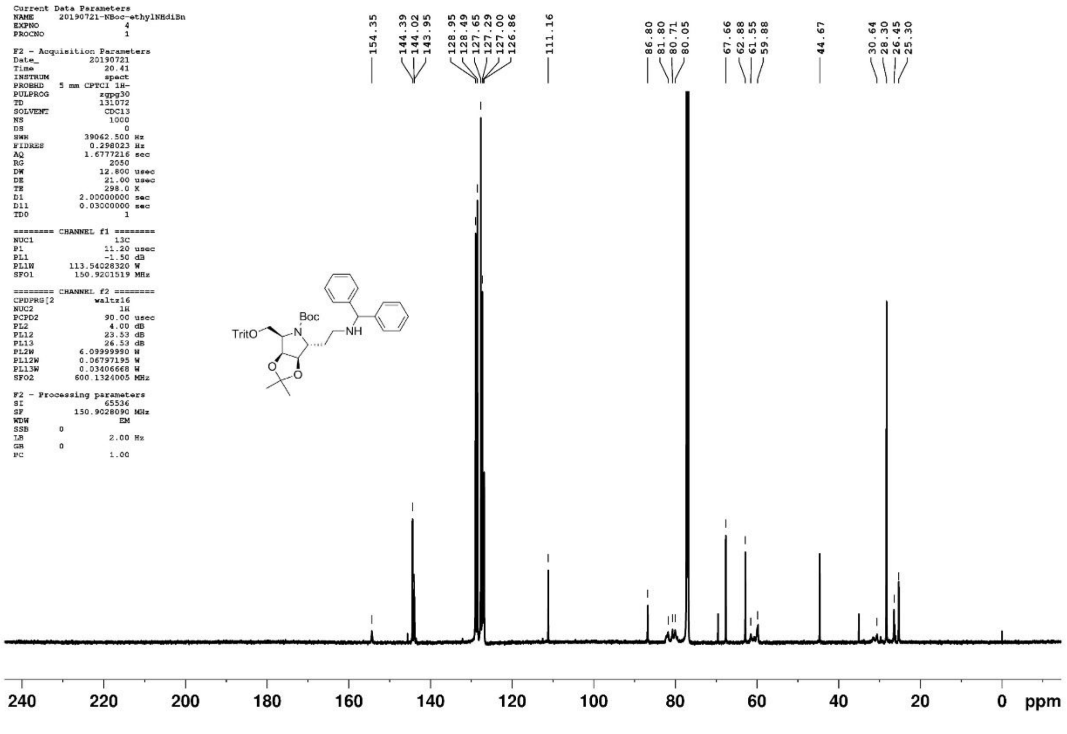

^{13}C spectrum of compound **S10 (**150 MHz, $CDCl_3$)

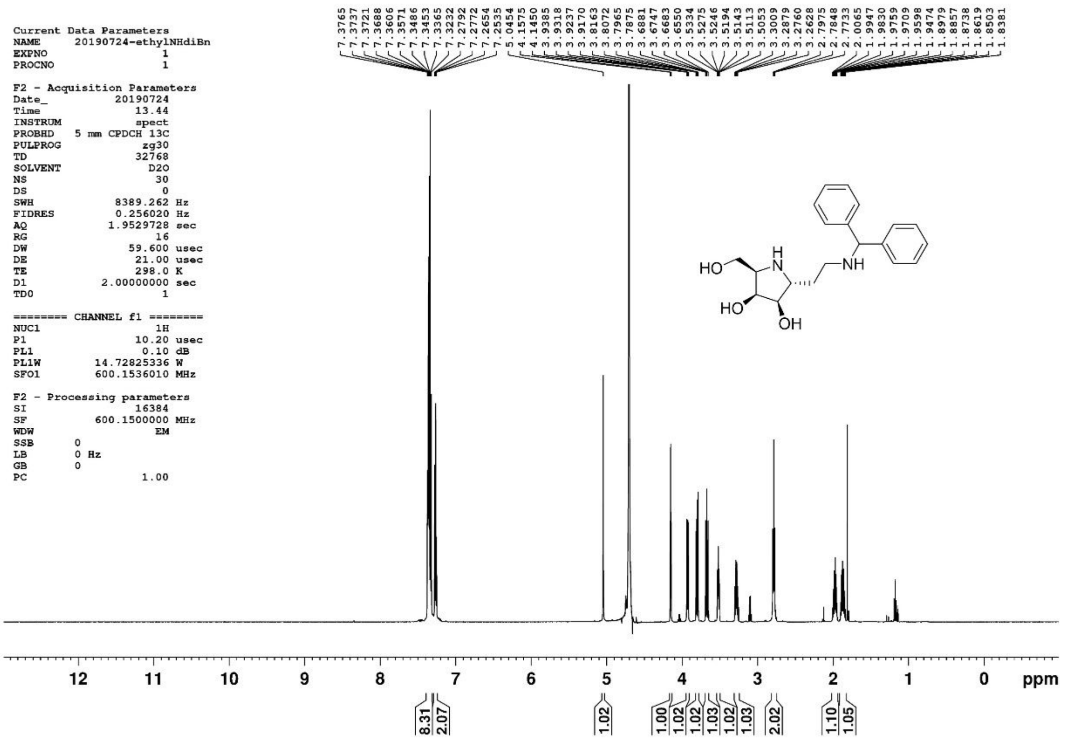

^{1}H spectrum of compound **12 (**600 MHz, D_2O)

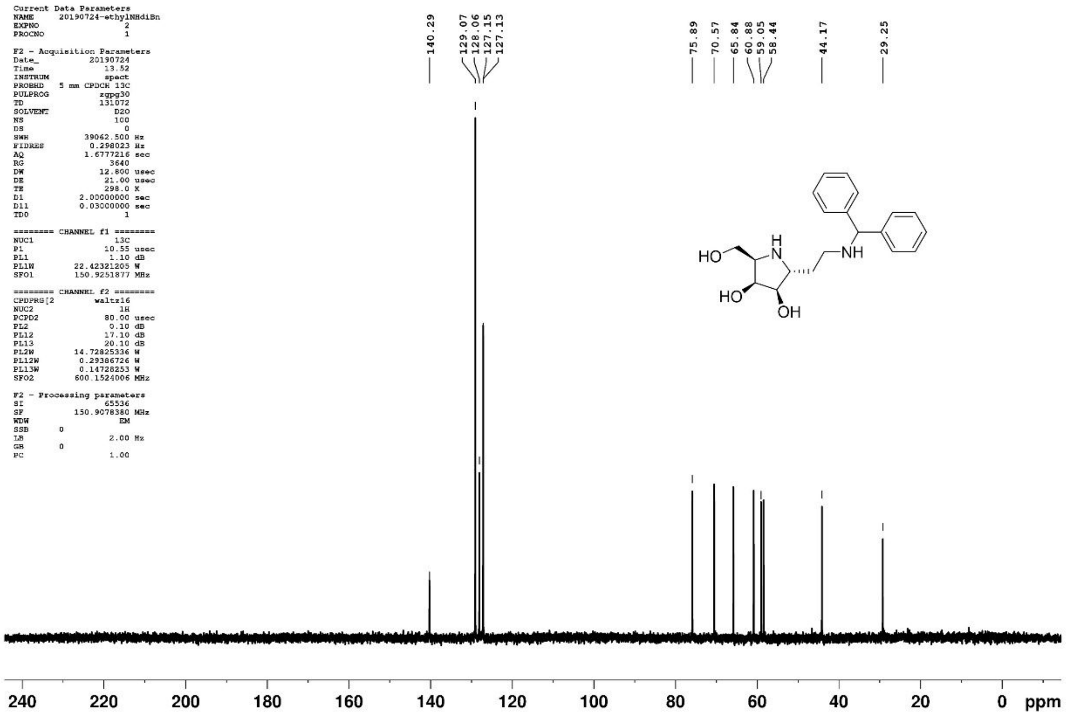

^{13}C spectrum of compound **12 (**150 MHz, D_2O)

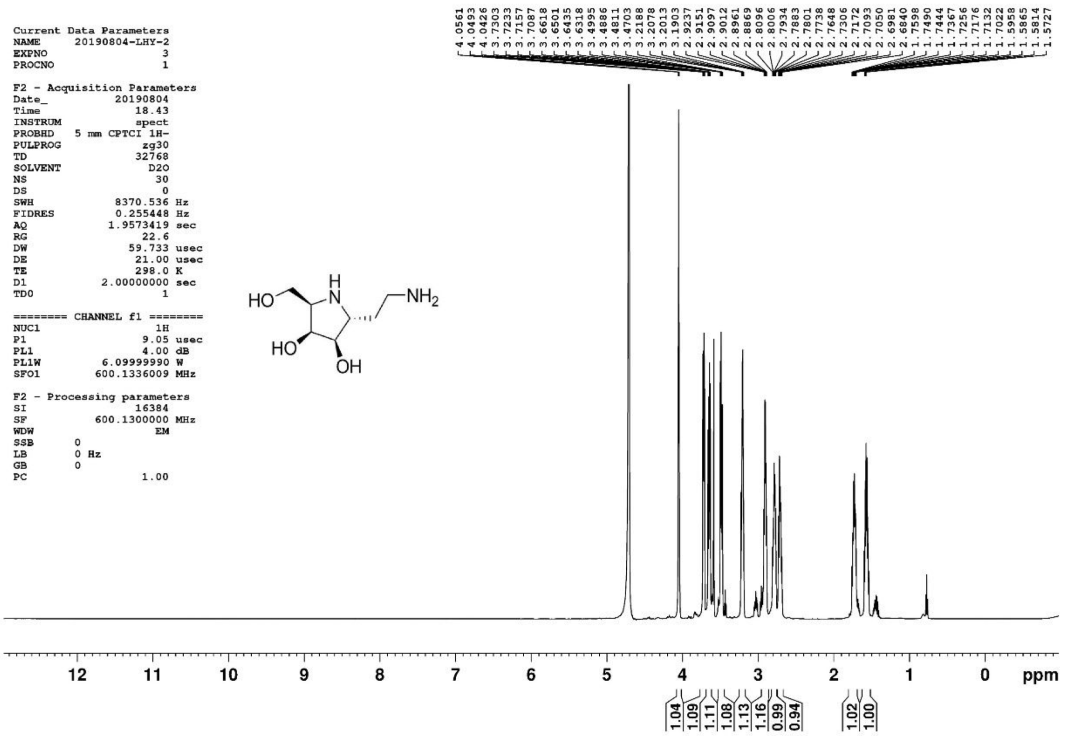

^{1}H spectrum of compound **13 (**600 MHz, D_2O)

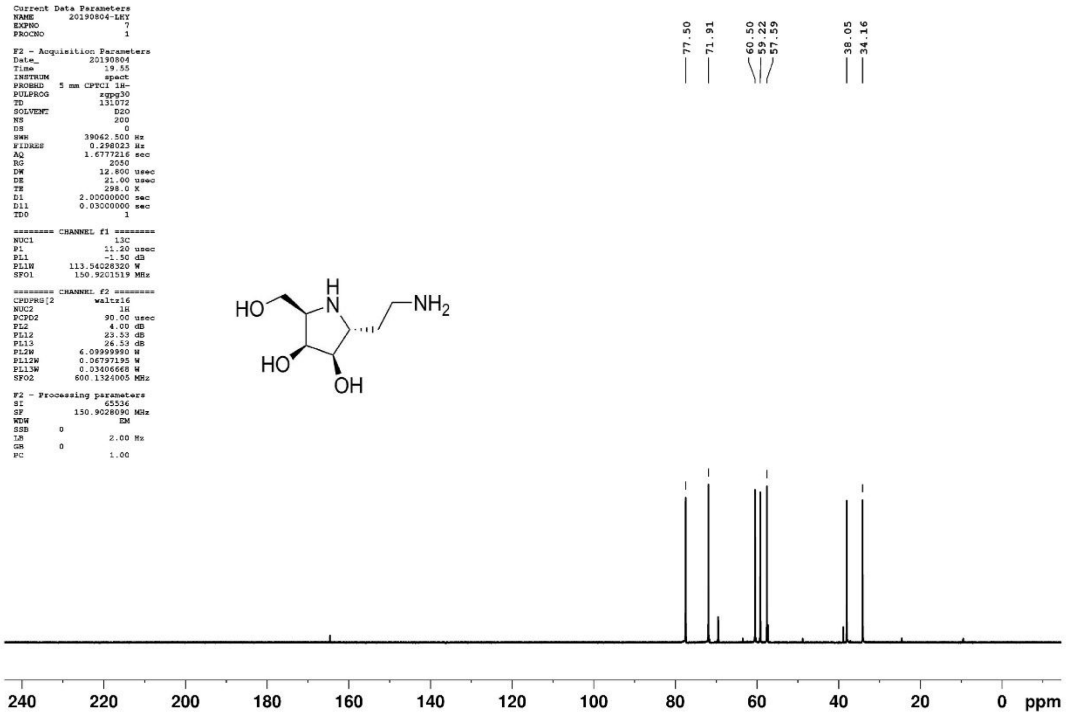

^{13}C spectrum of compound **13 (**150 MHz, D_2O)

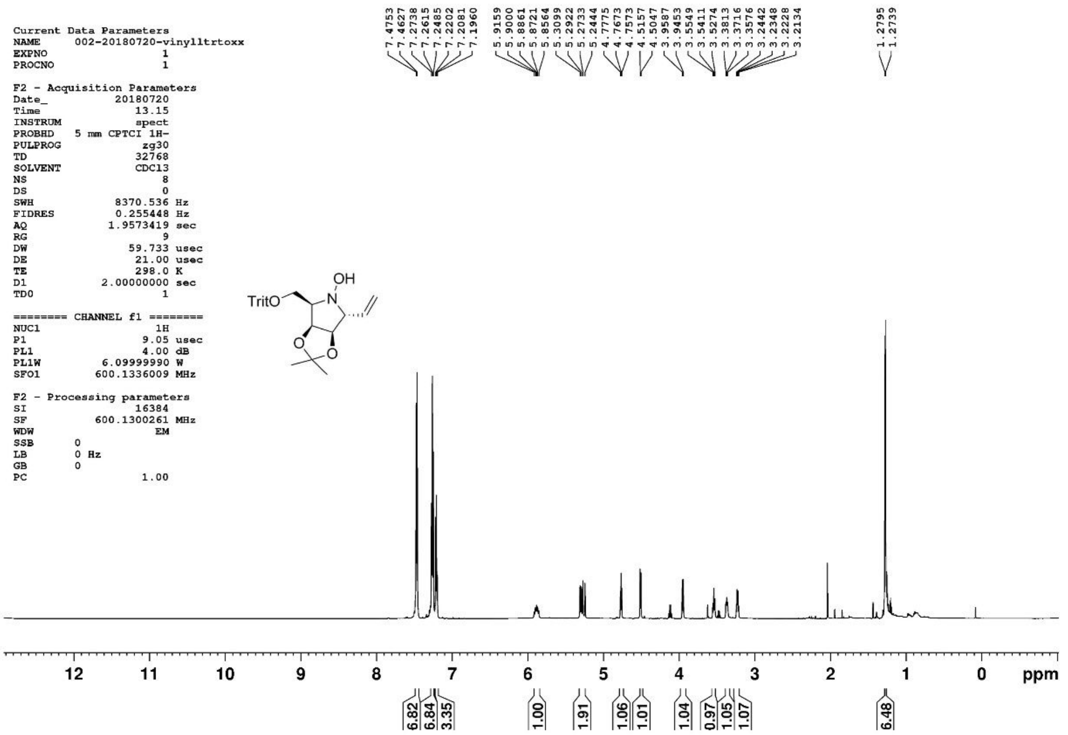

^{1}H spectrum of compound **S11 (**600 MHz, $CDCl_3$)

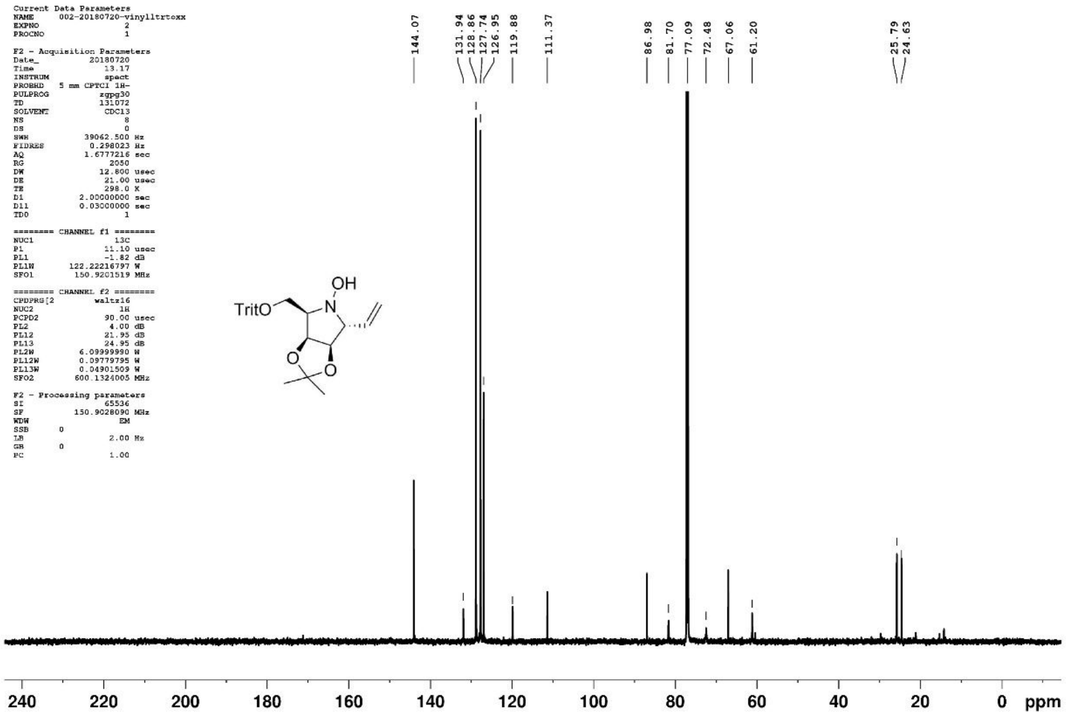

^{13}C spectrum of compound **S11 (**150 MHz, $CDCl_3$)

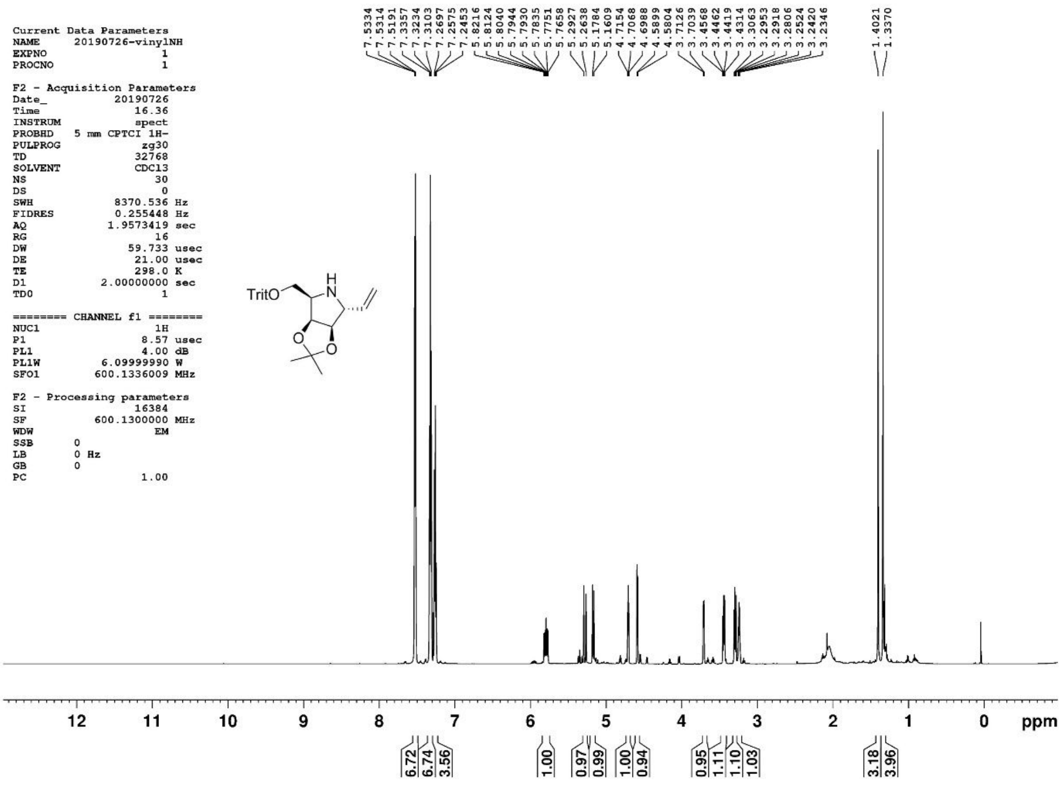

^{1}H spectrum of compound **S12 (**600 MHz, $CDCl_3$)

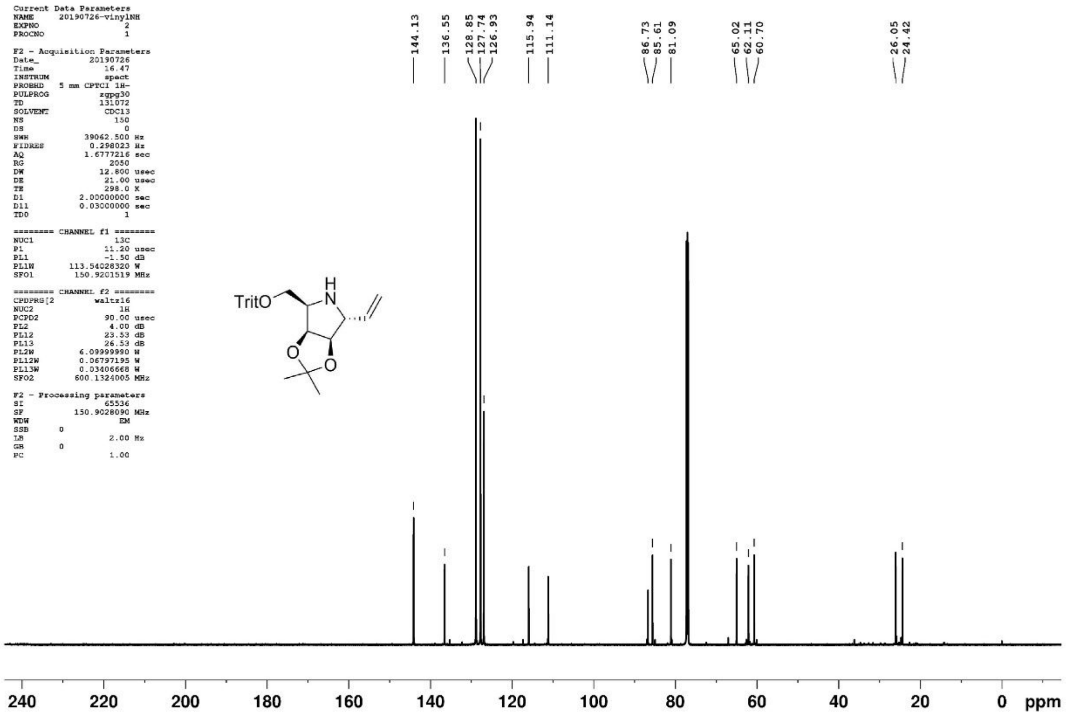

^{13}C spectrum of compound **S12 (**150 MHz, $CDCl_3$)

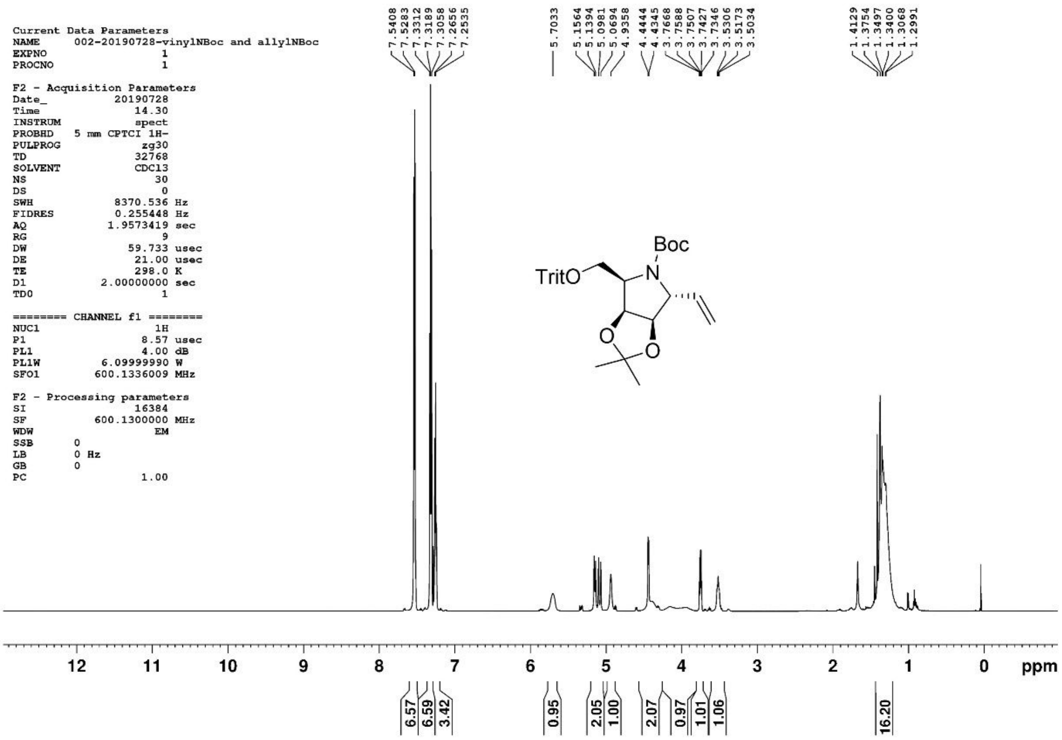

^{1}H spectrum of compound **14 (**600 MHz, $CDCl_3$)

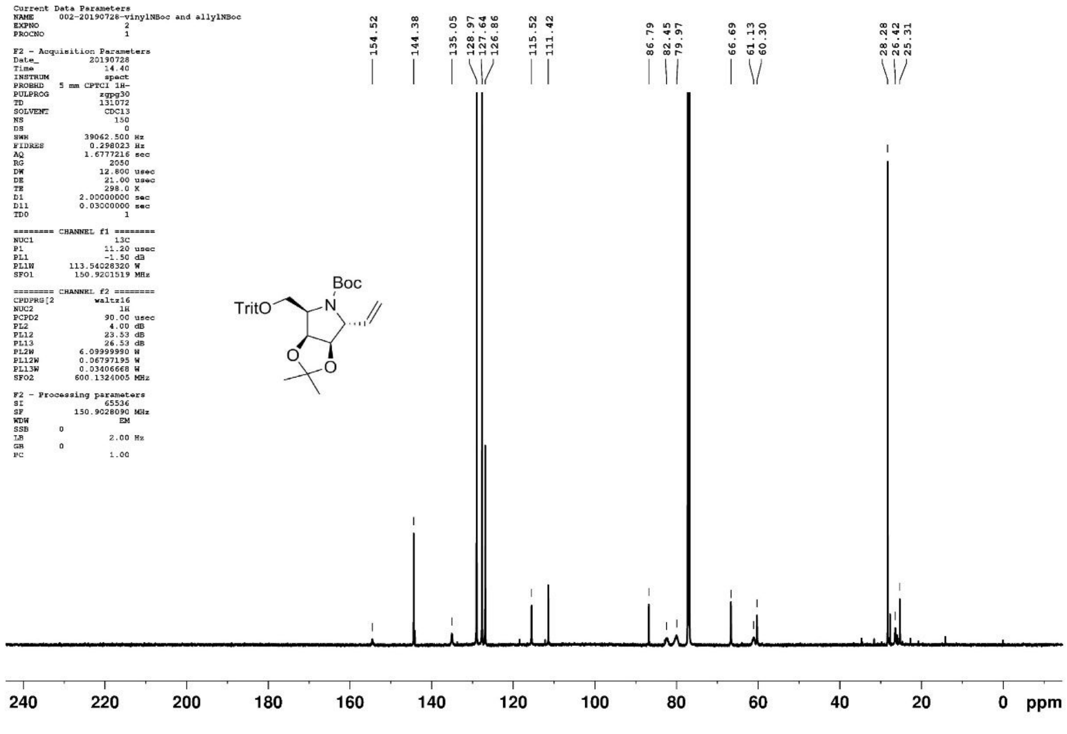

^{13}C spectrum of compound **14 (**150 MHz, $CDCl_3$)

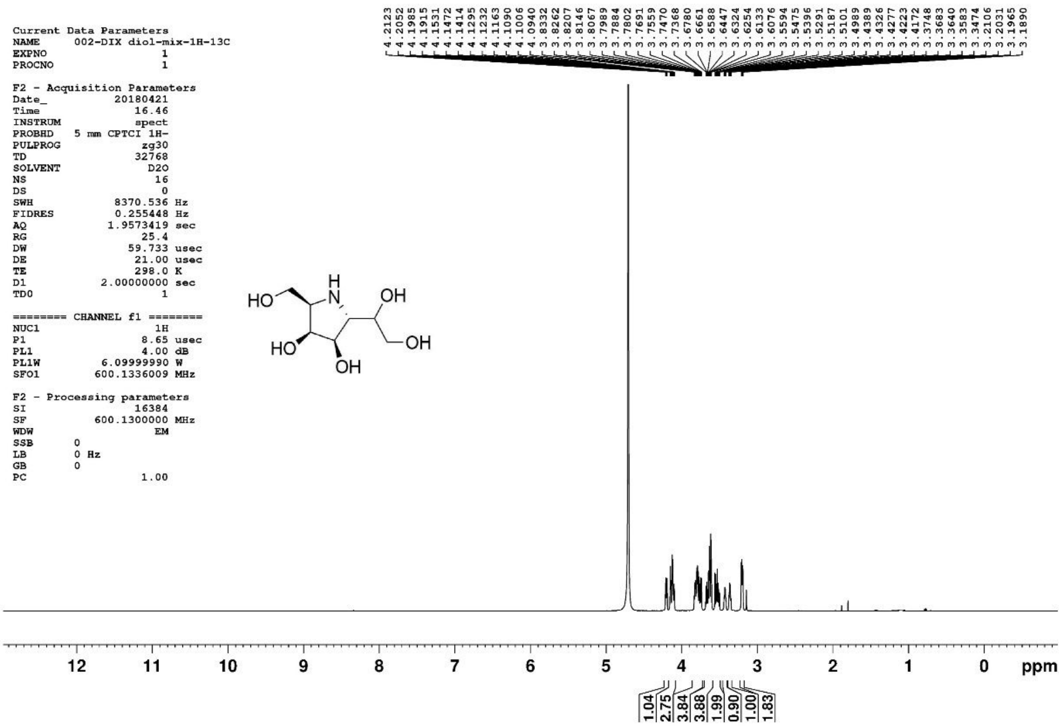

^{1}H spectrum of compound **15 (**600 MHz, D_2O)

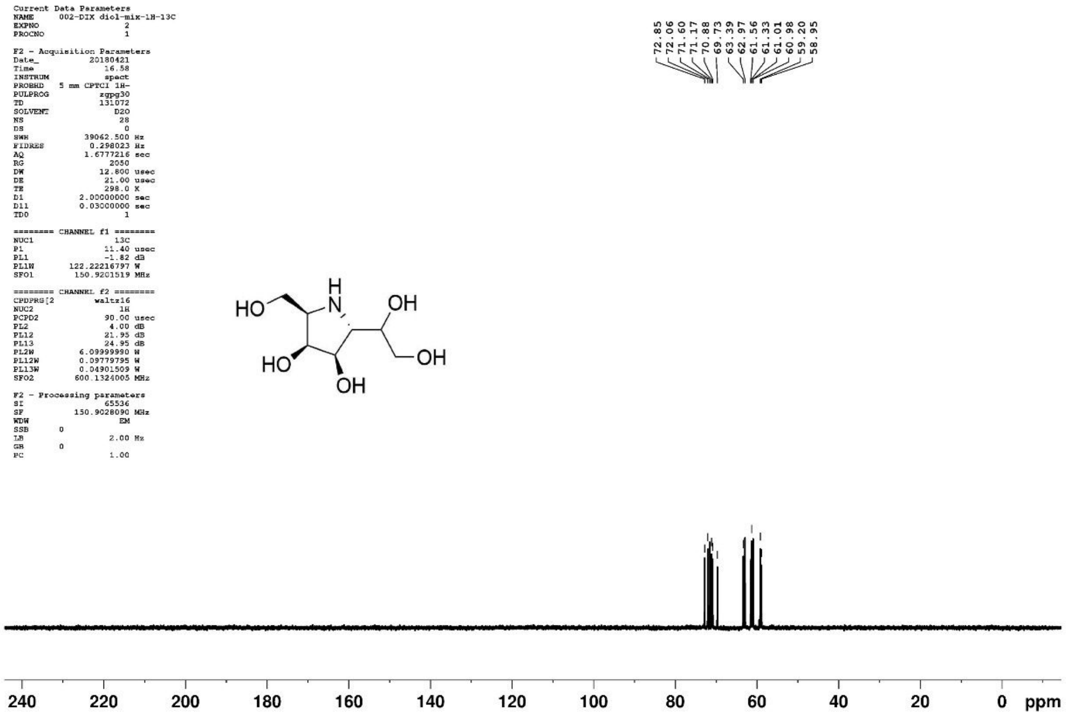

^{13}C spectrum of compound **15 (**150 MHz, D_2O)

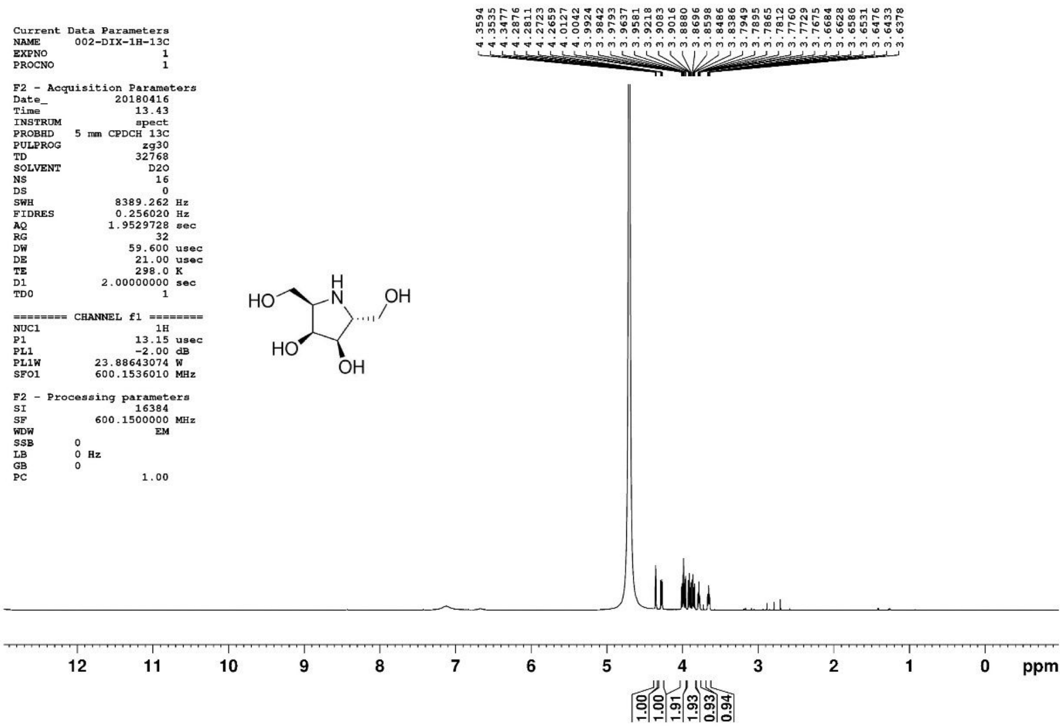

^{1}H spectrum of compound **4** (600 MHz, D_2O)

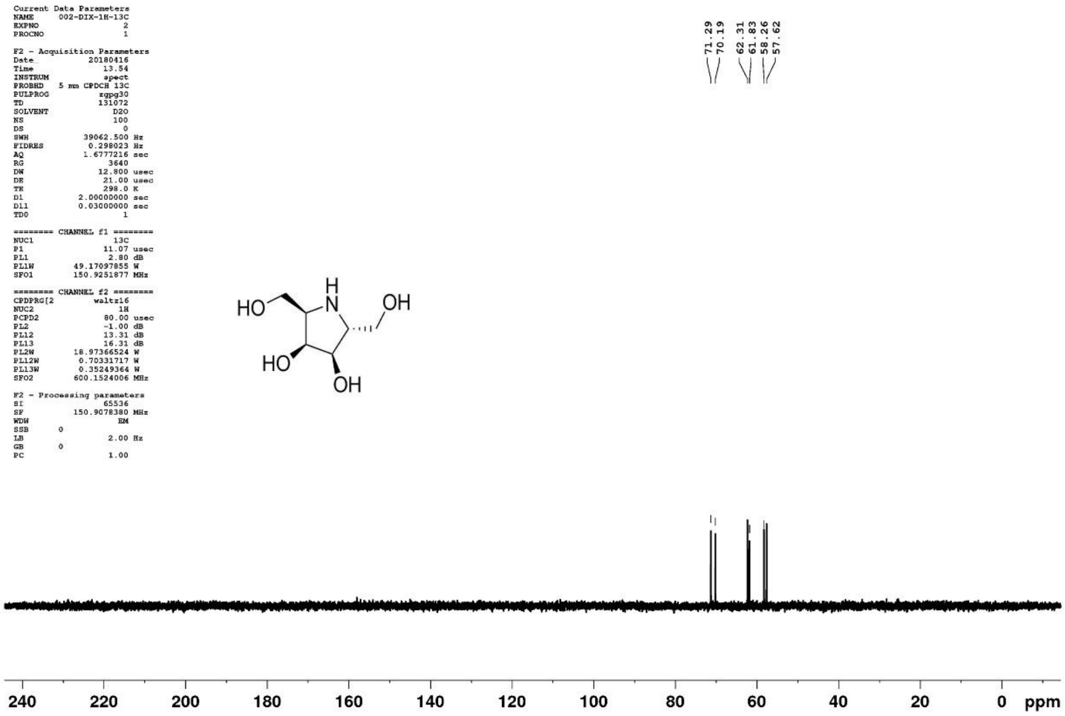

^{13}C spectrum of compound **4** (150 MHz, D_2O)

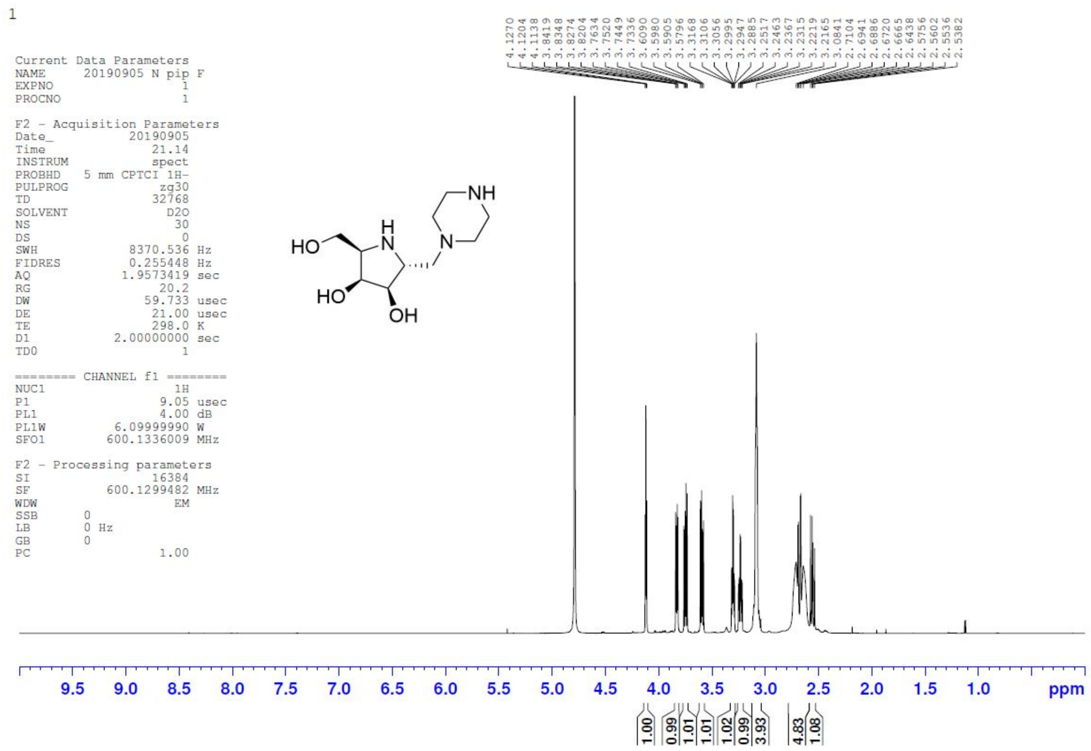

^{1}H spectrum of compound **17 (**600 MHz, D_2O)

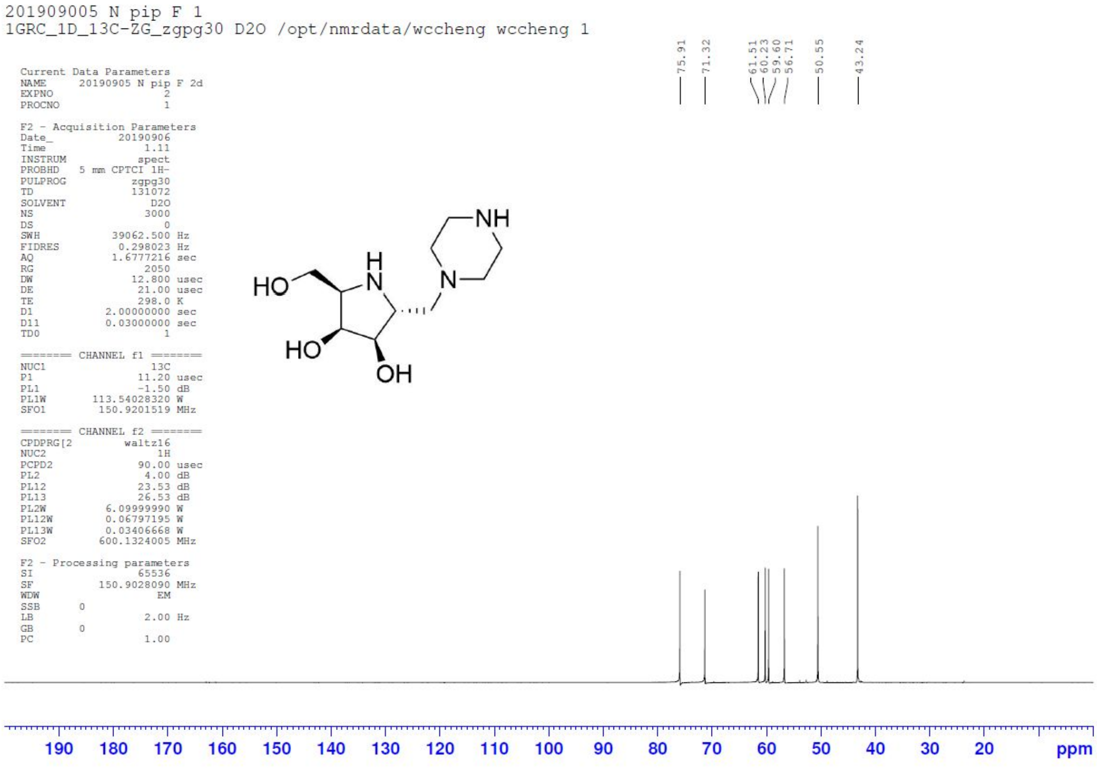

^{13}C spectrum of compound **17 (**150 MHz, D_2O)

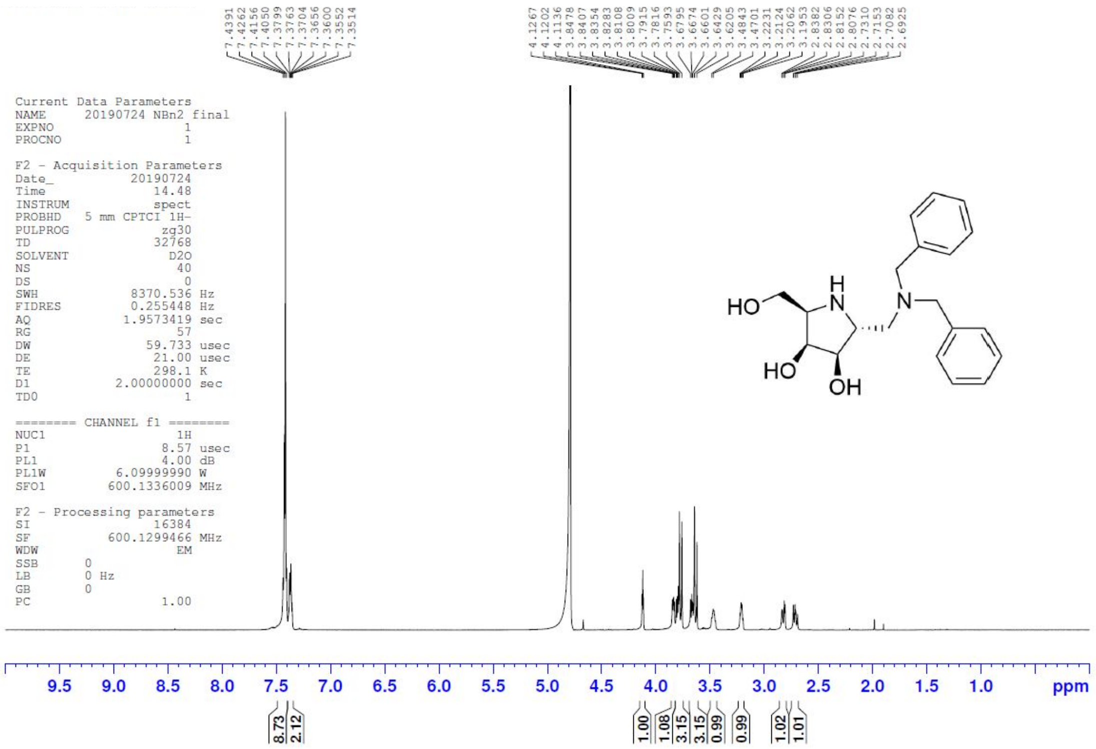

^{1}H spectrum of compound **18** (600 MHz, D_2O)

1GRC_1D_13C-ZG_zgpg30 D2O /opt/nmrdata/wccheng wccheng 4

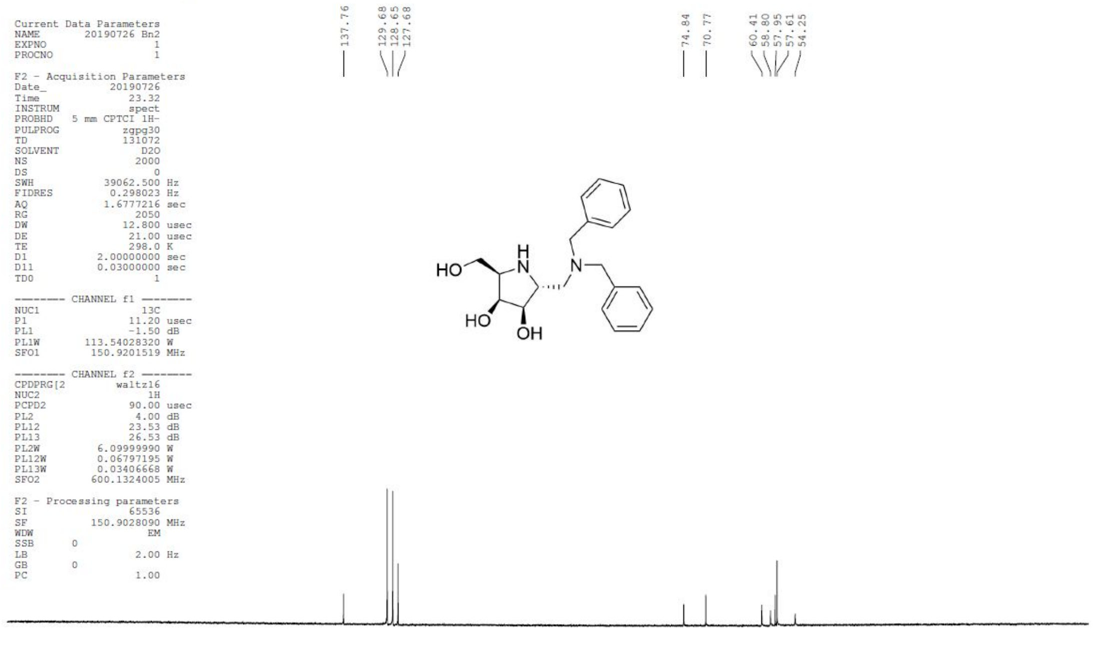

^{13}C spectrum of compound **18** (150 MHz, D_2O)

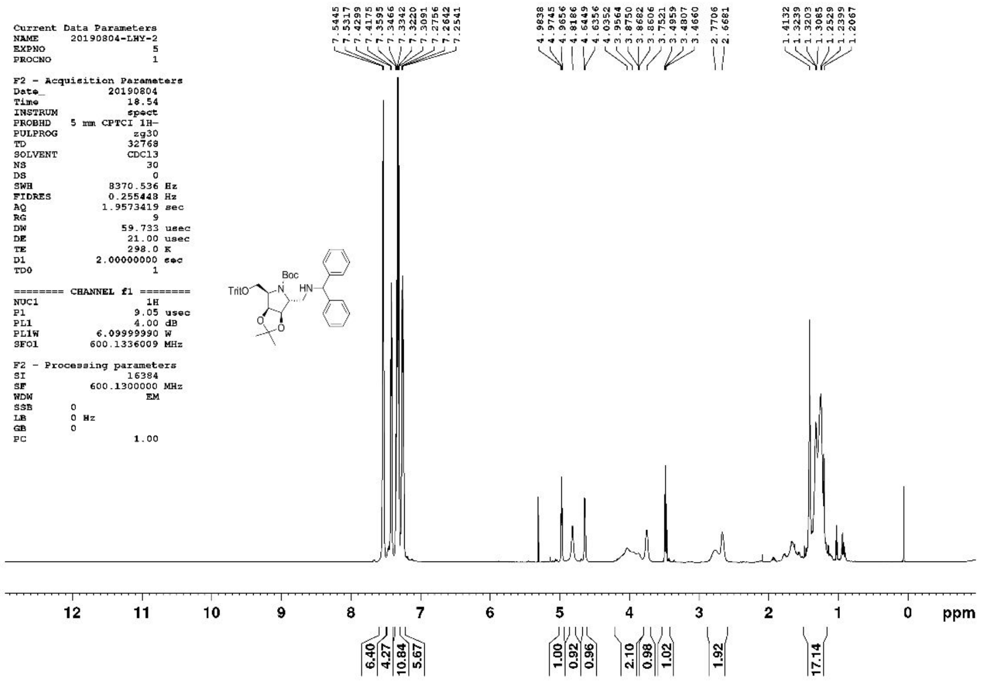

^{1}H spectrum of compound **S1 (**600 MHz, $CDCl_3$)

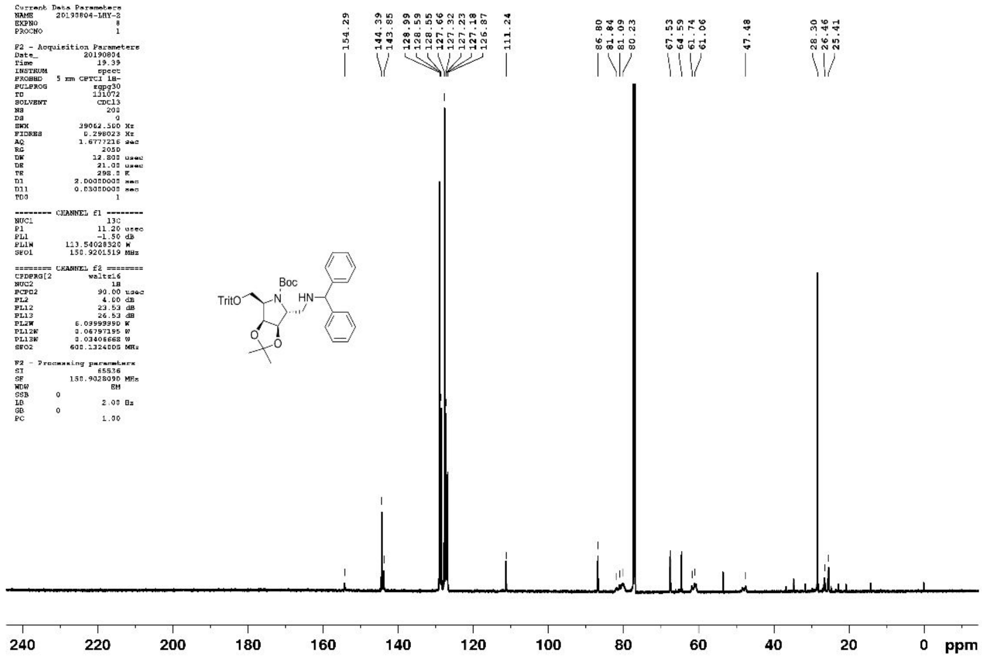

^{13}C spectrum of compound **S1 (**150 MHz, $CDCl_3$)

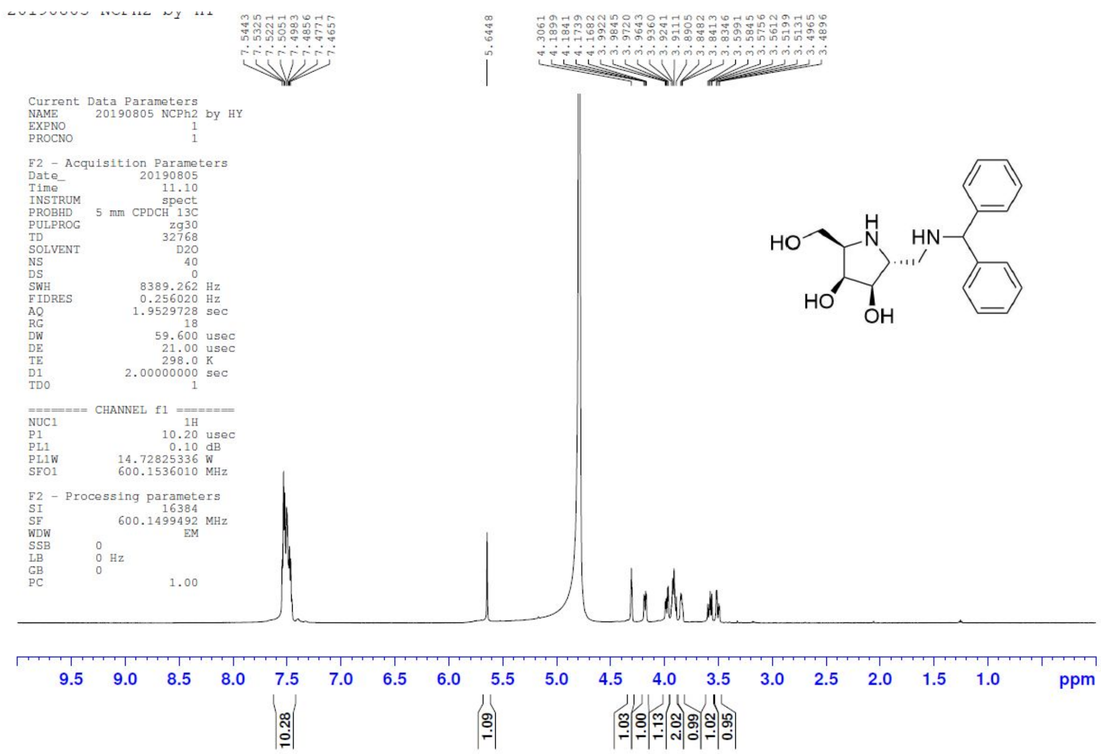

^{1}H spectrum of compound **19 (**600 MHz, D_2O)

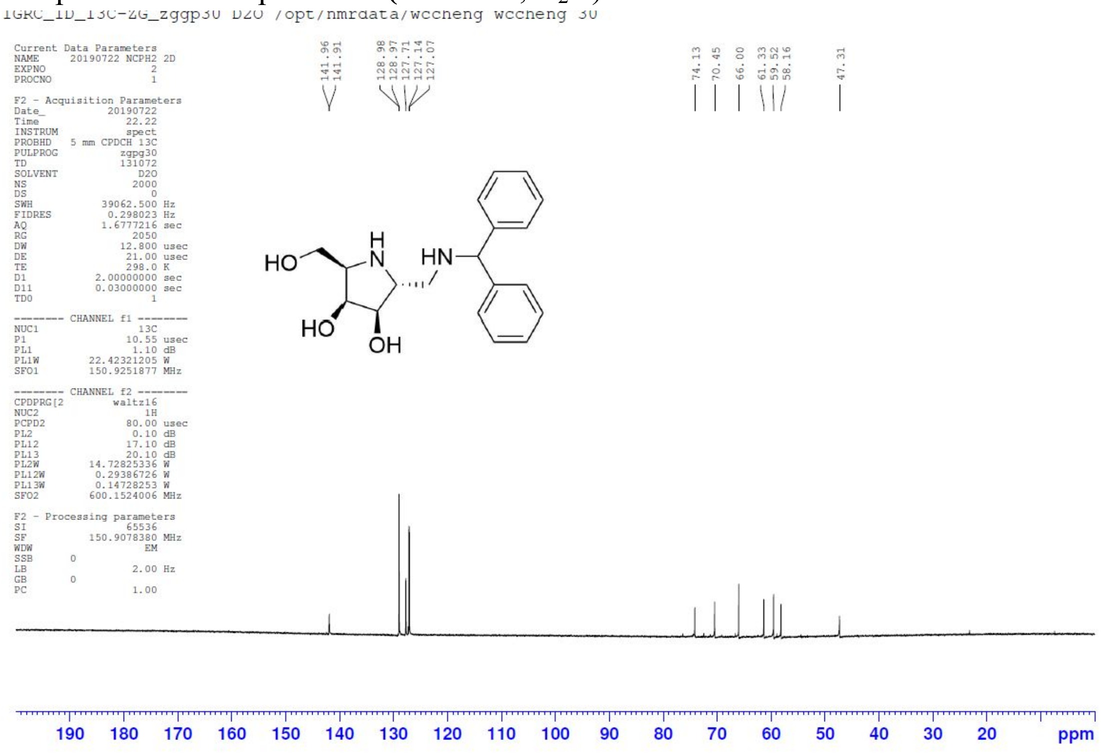

^{13}C spectrum of compound **19 (**150 MHz, D_2O)

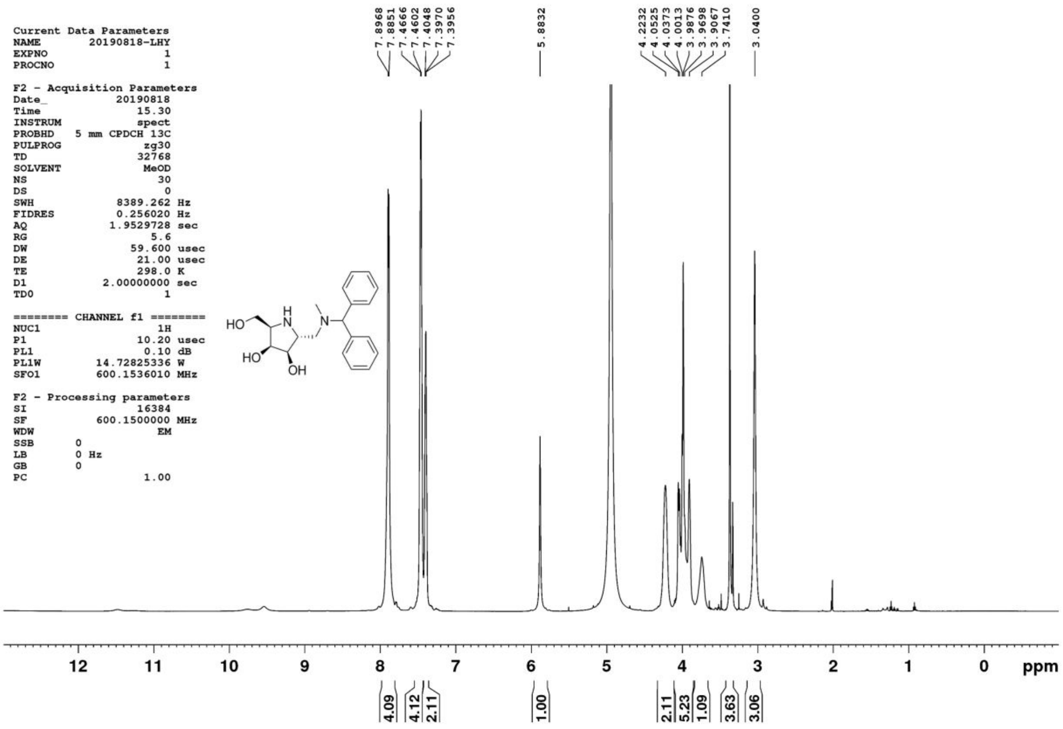

^{1}H spectrum of compound **S2 (**600 MHz, MeOD)

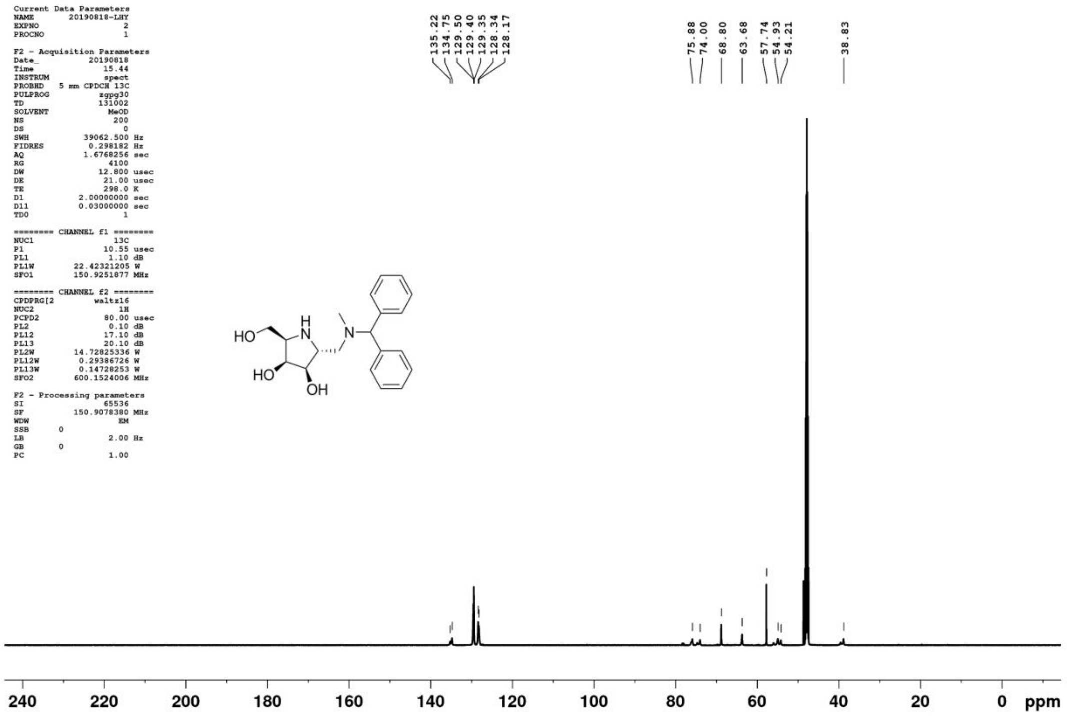

^{13}C spectrum of compound **S2 (**150 MHz, MeOD)

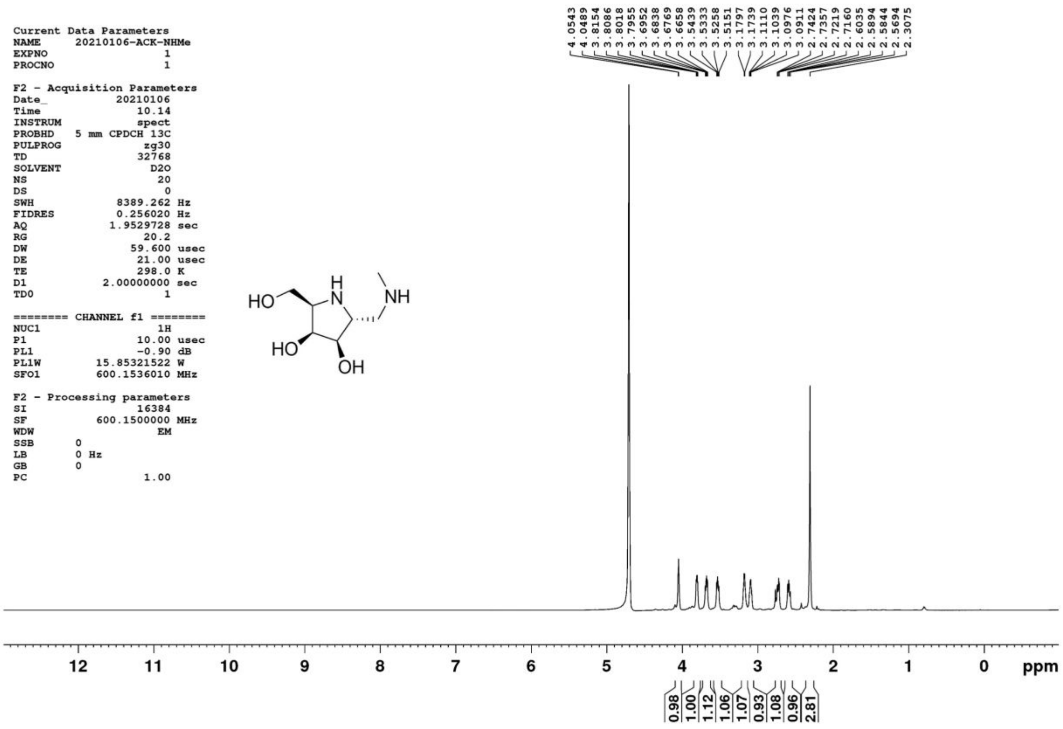

^{1}H spectrum of compound **20 (**600 MHz, D_2O)

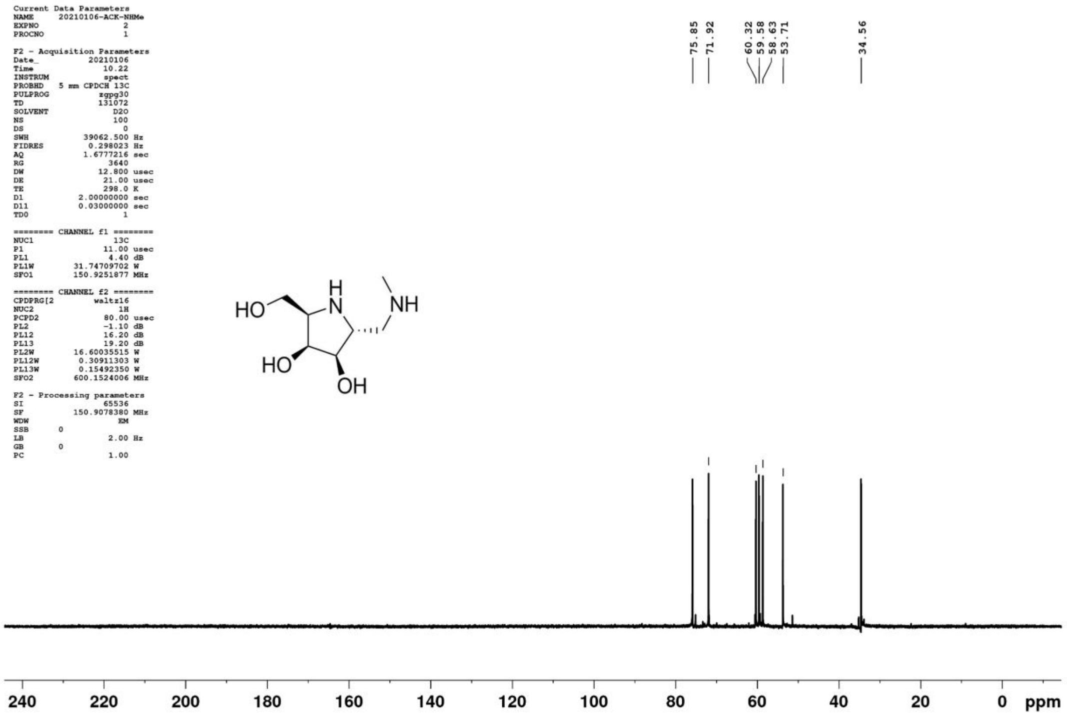

^{13}C spectrum of compound **20 (**150 MHz, D_2O)

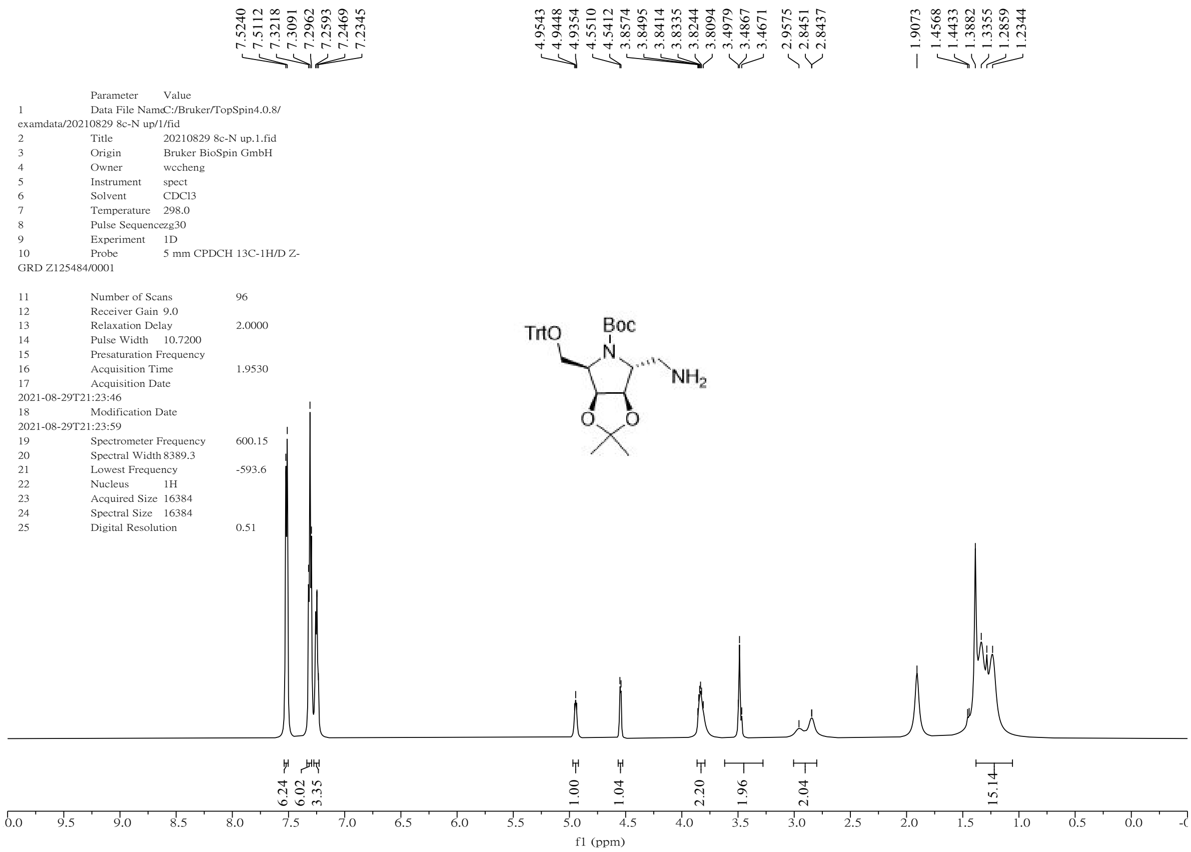

^1H spectrum of compound **S3** (600 MHz, $CDCl_3$)

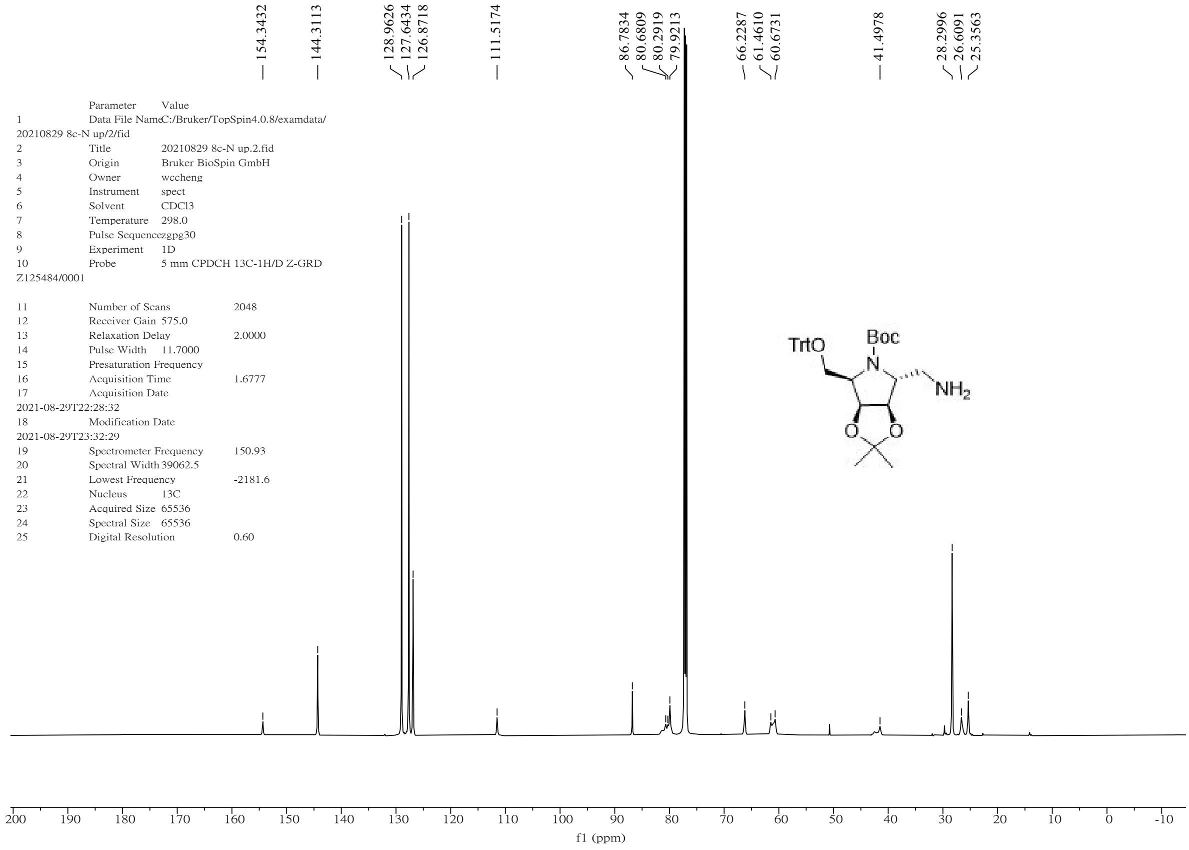

^{13}C spectrum of compound **S3** (150 MHz, $CDCl_3$)

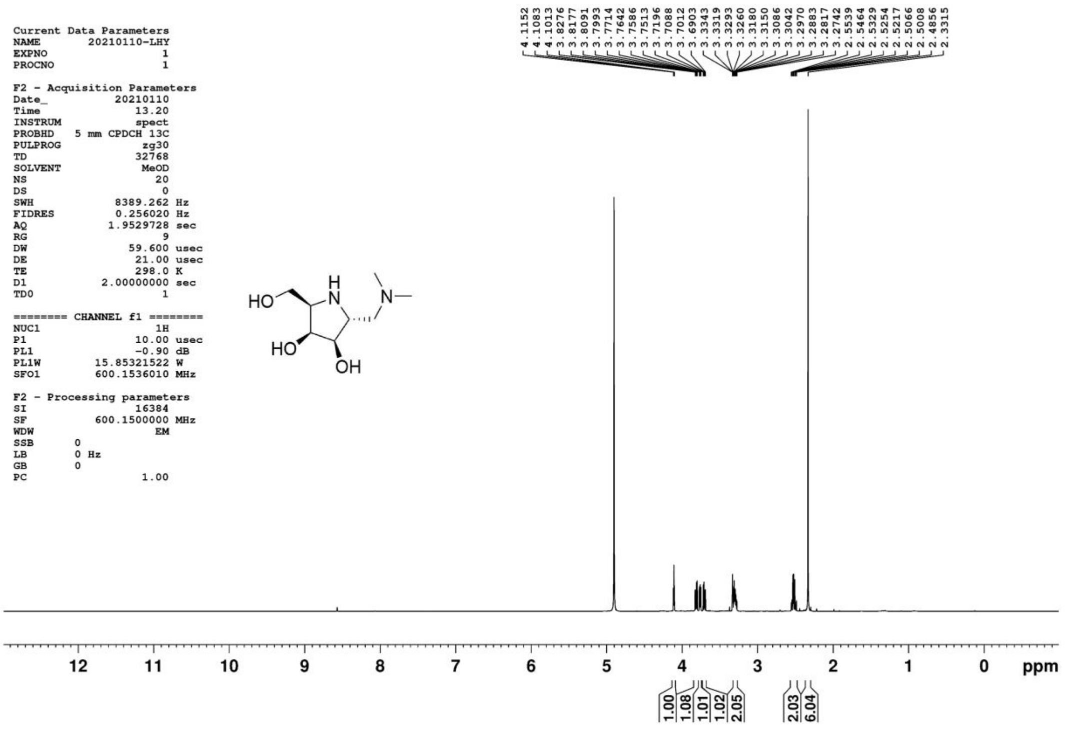

^{1}H spectrum of compound **21 (**600 MHz, MeOD)

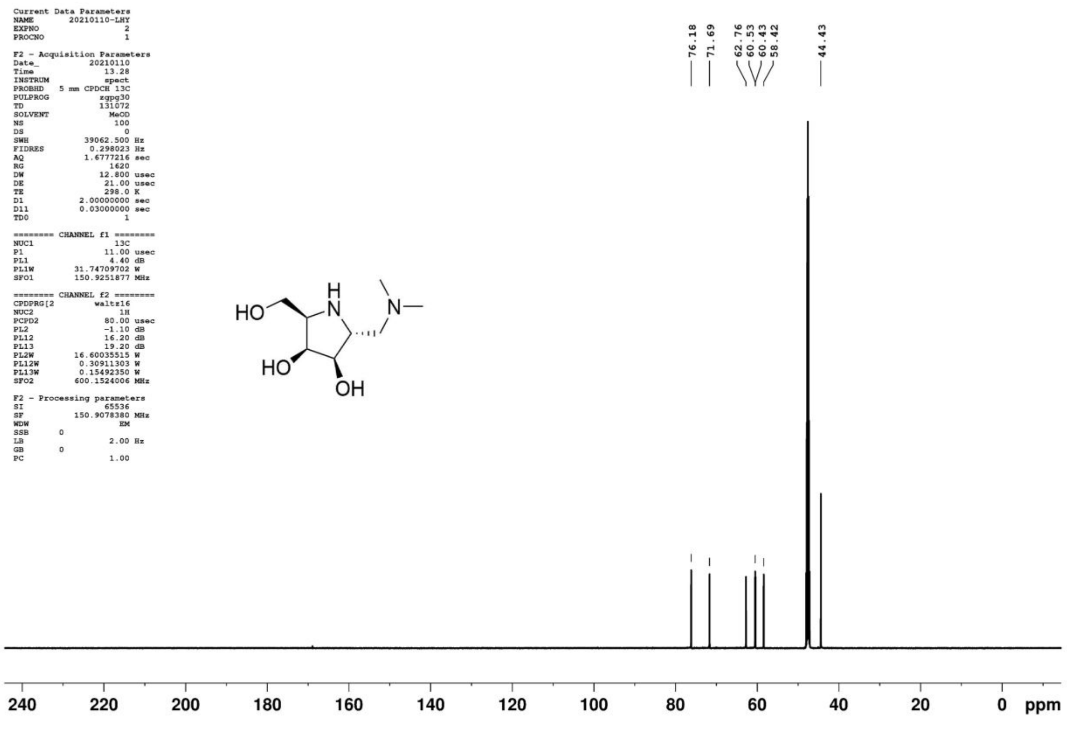

^{13}C spectrum of compound **21 (**150 MHz, MeOD)

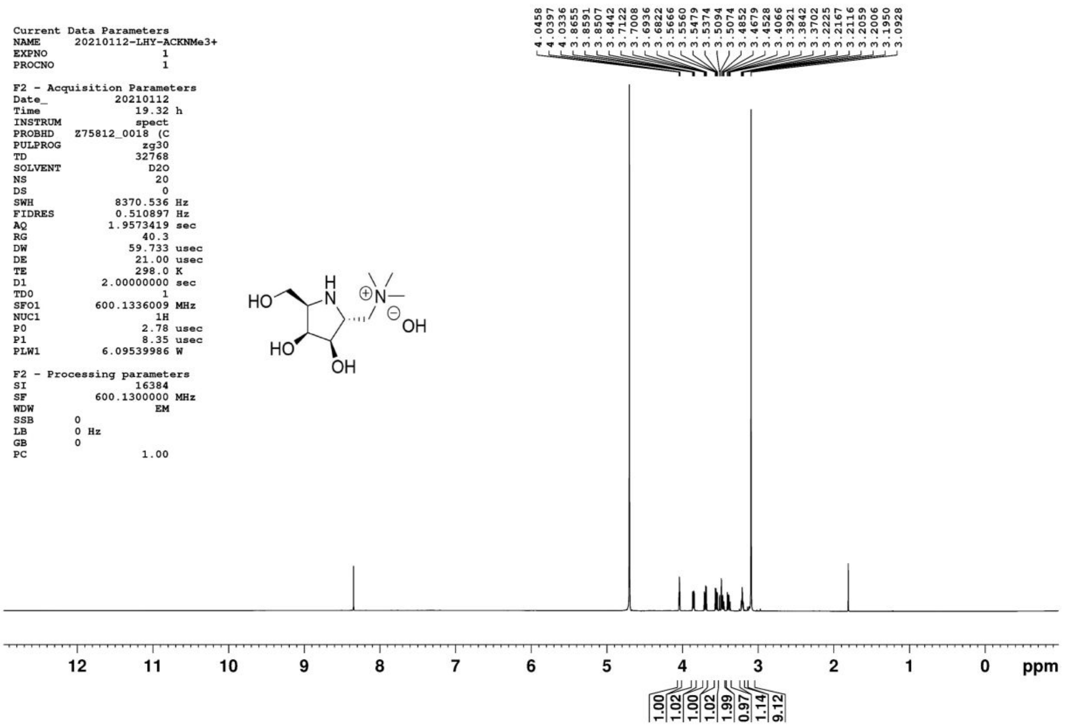

^{1}H spectrum of compound **22 (**600 MHz, D_2O)

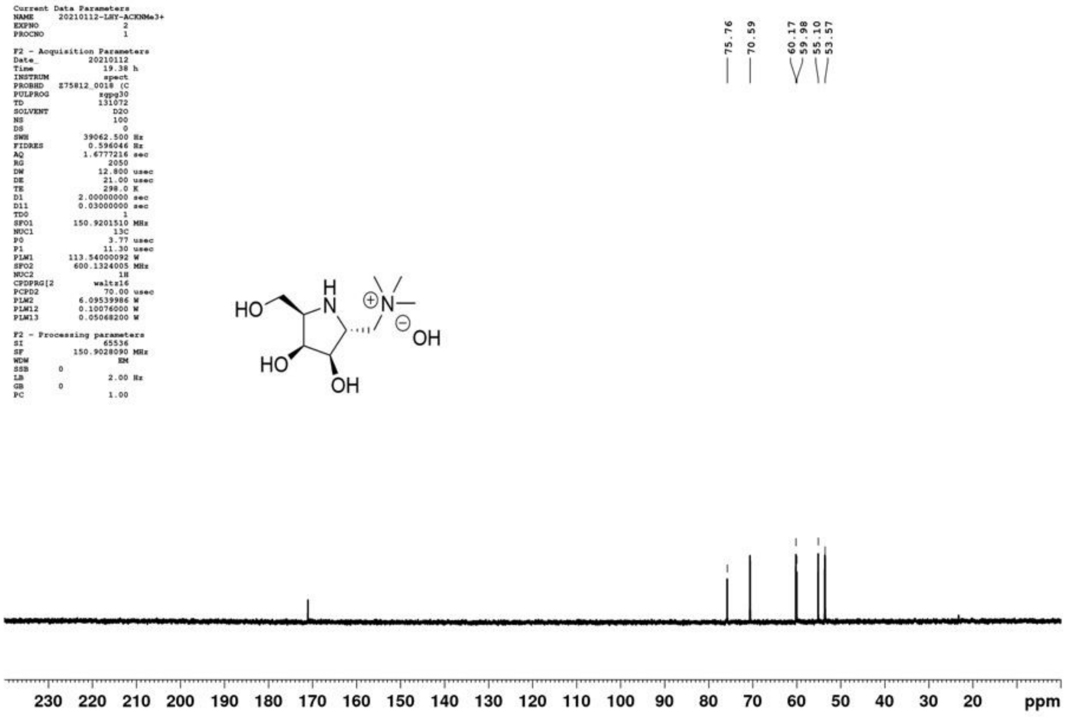

^{13}C spectrum of compound **22 (**150 MHz, D_2O)

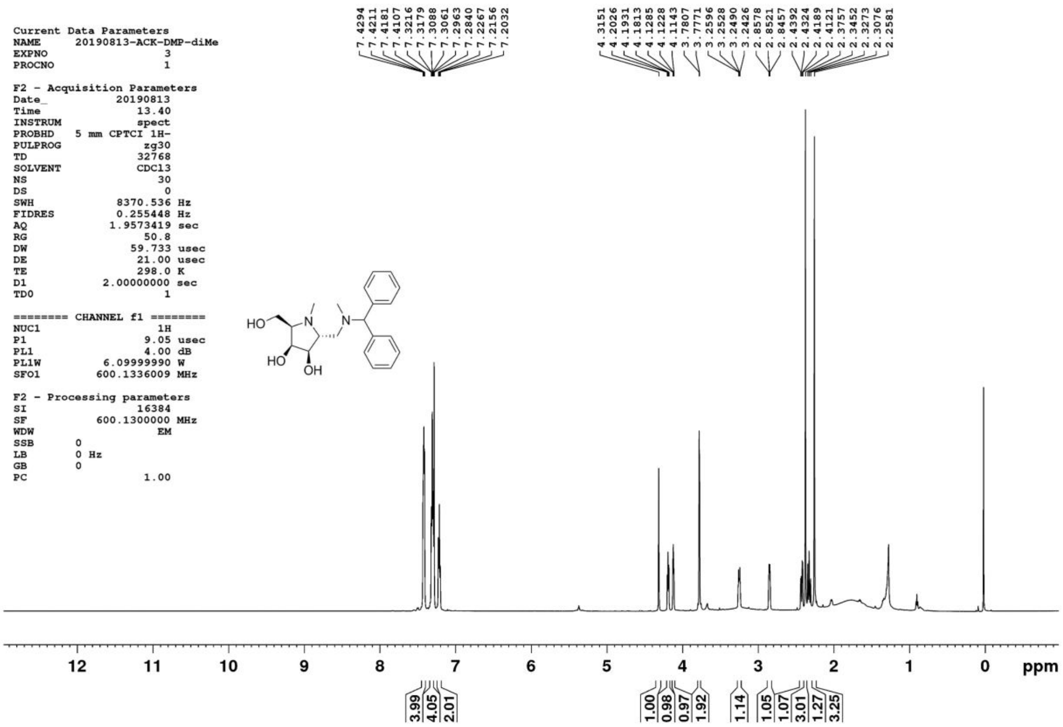

^{1}H spectrum of compound **S4 (**600 MHz, $CDCl_3$)

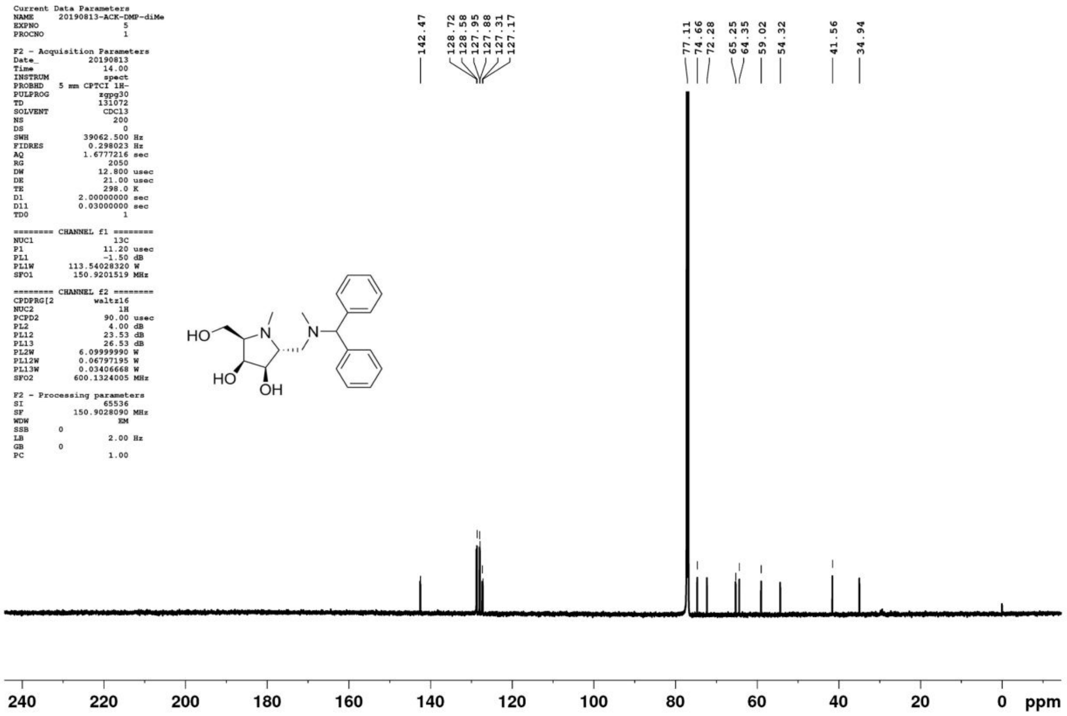

^{13}C spectrum of compound **S4 (**150 MHz, $CDCl_3$)

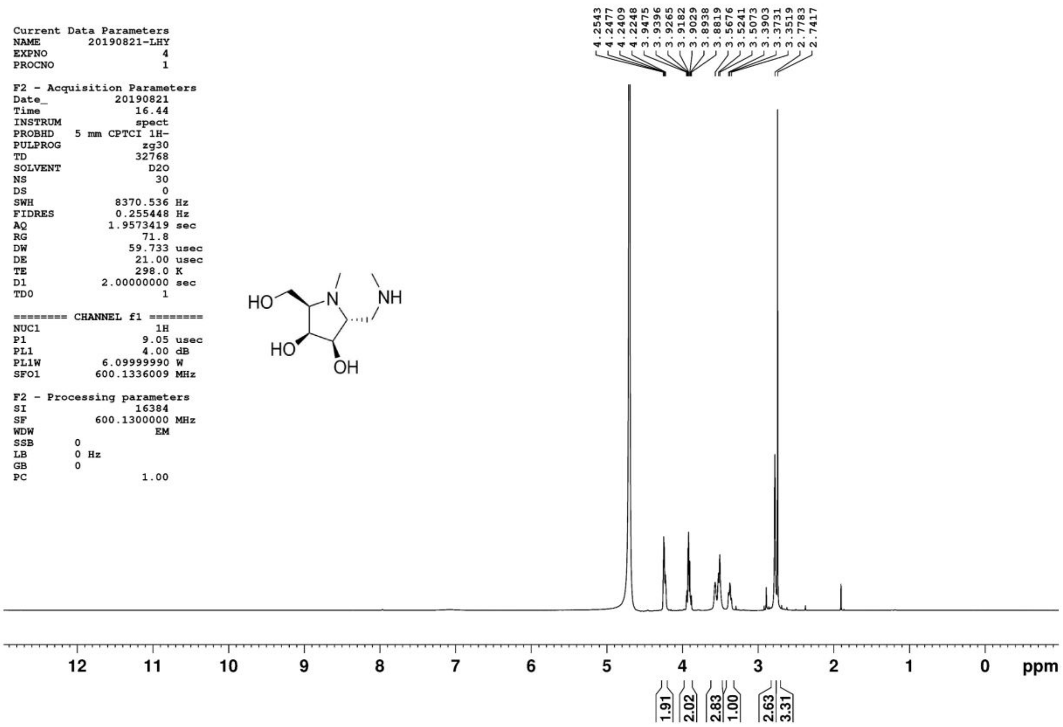

^{1}H spectrum of compound **S5 (**600 MHz, D_2O)

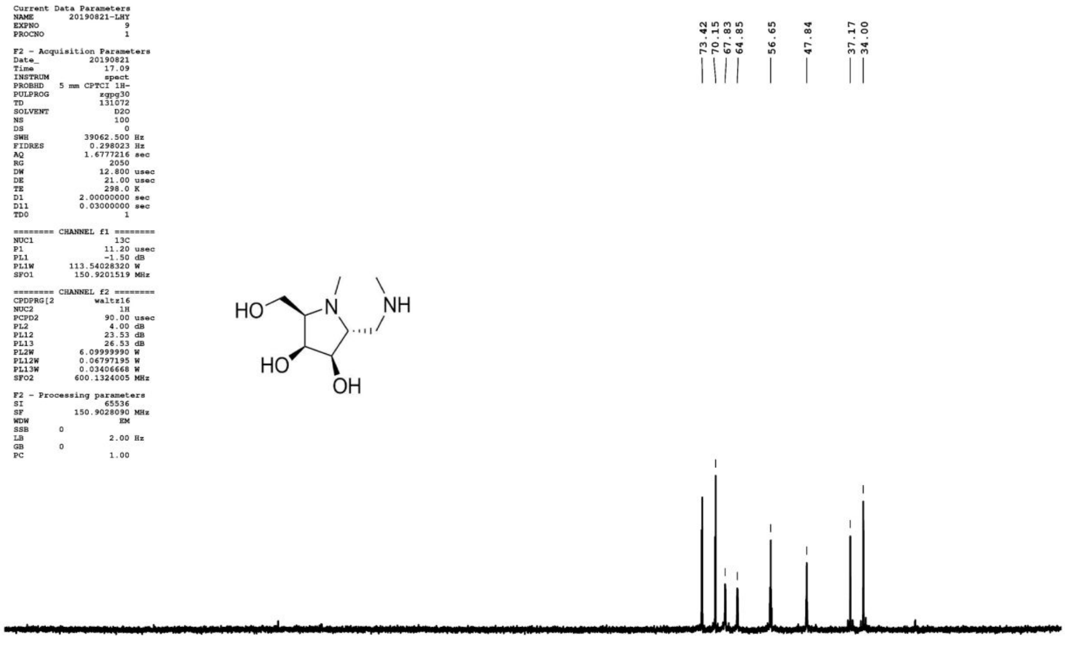

^{13}C spectrum of compound **S5 (**150 MHz, D_2O)

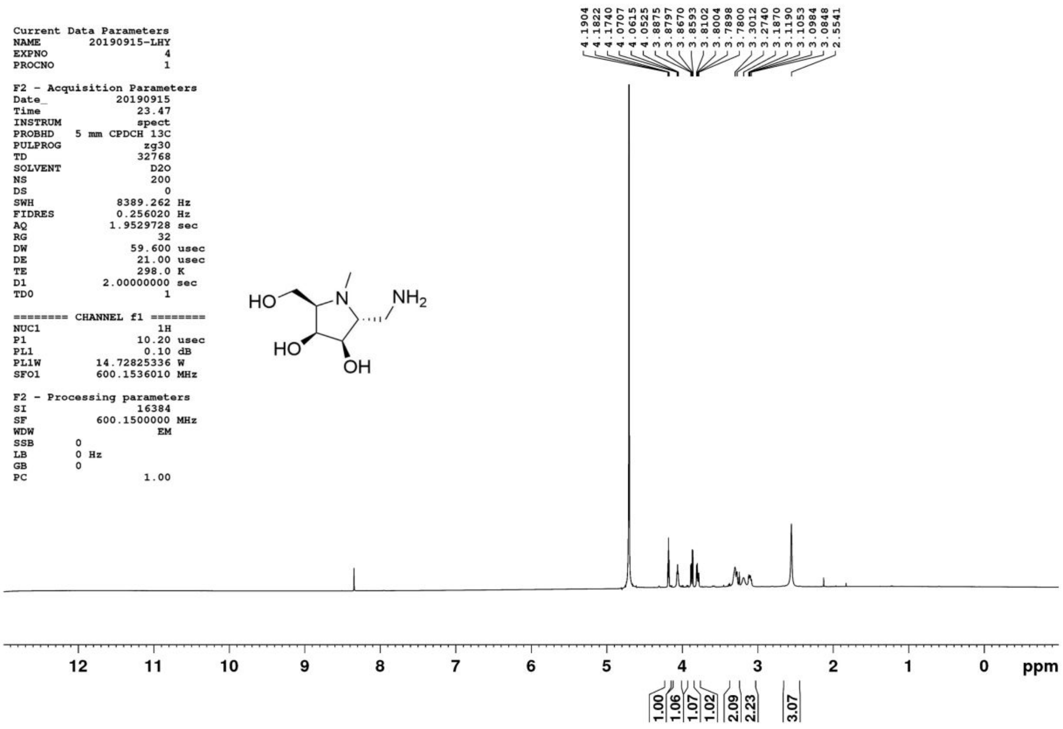

^{1}H spectrum of compound **23 (**600 MHz, D_2O)

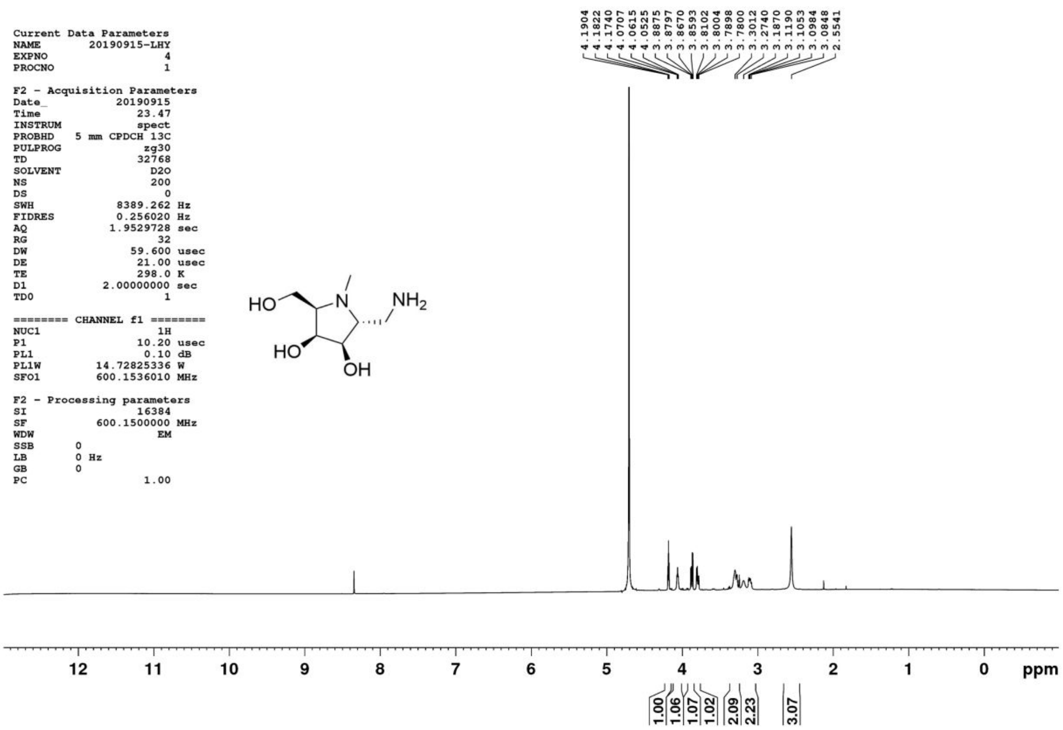

^{13}C spectrum of compound **23 (**150 MHz, D_2O)

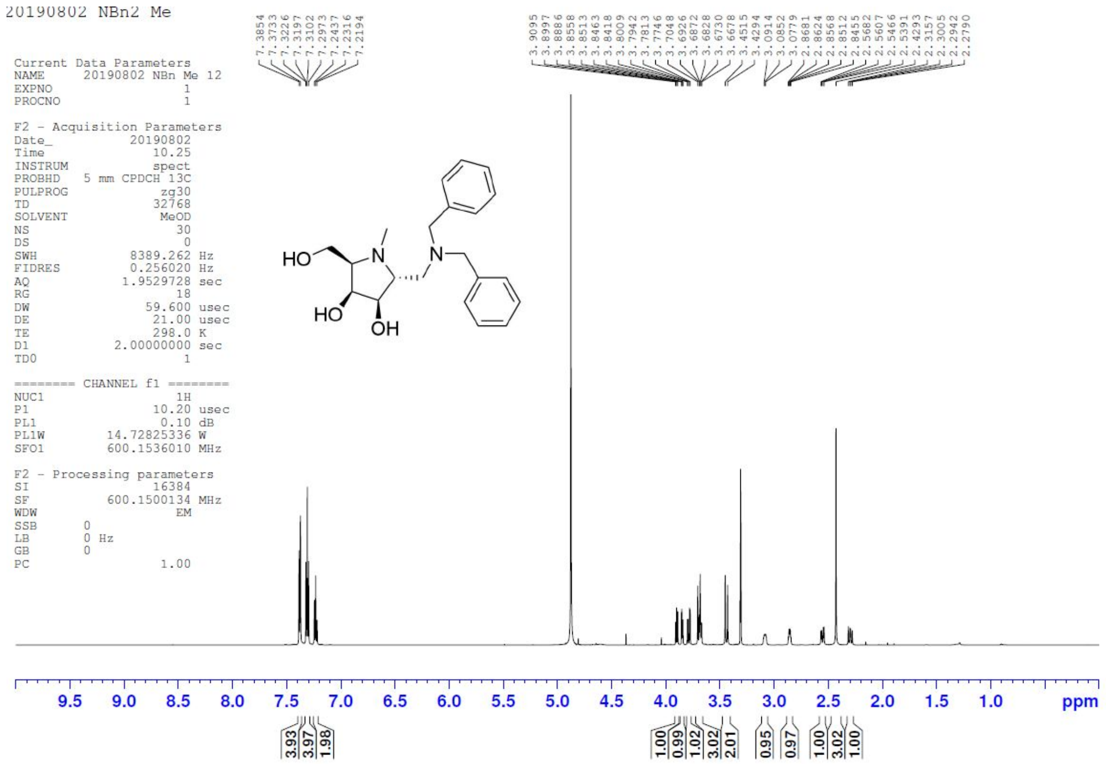

^{1}H spectrum of compound **S6 (**600 MHz, MeOD)

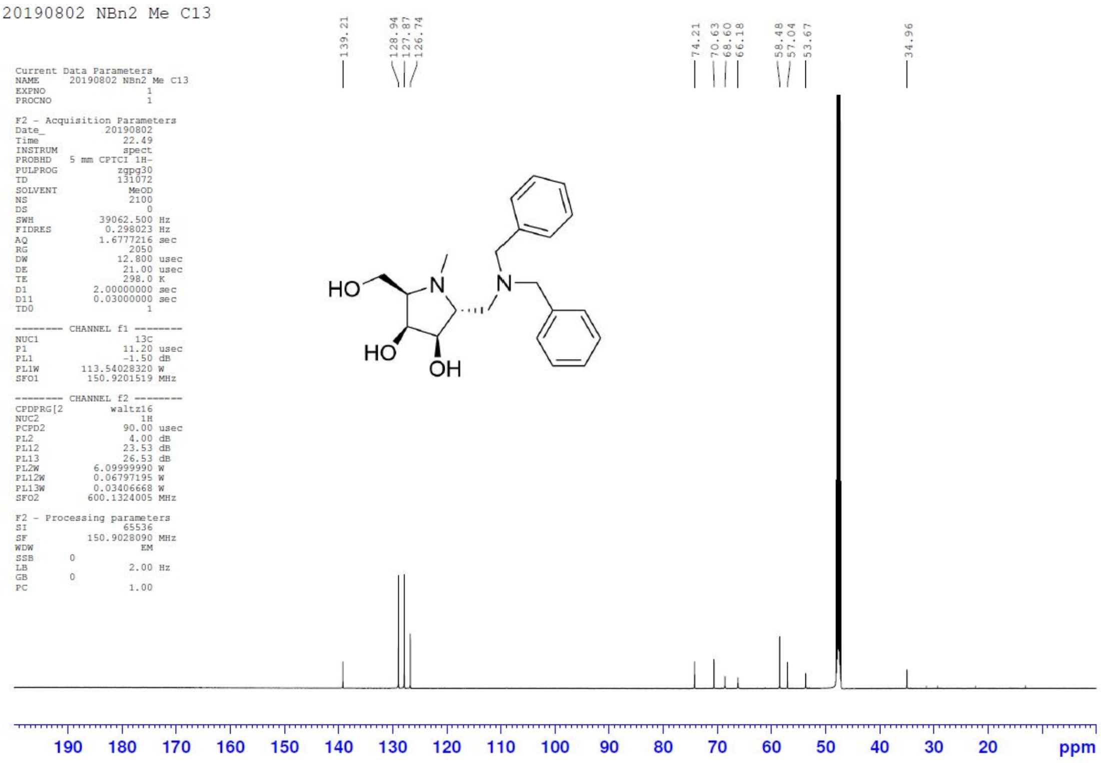

^{13}C spectrum of compound **S6 (**150 MHz, MeOD)

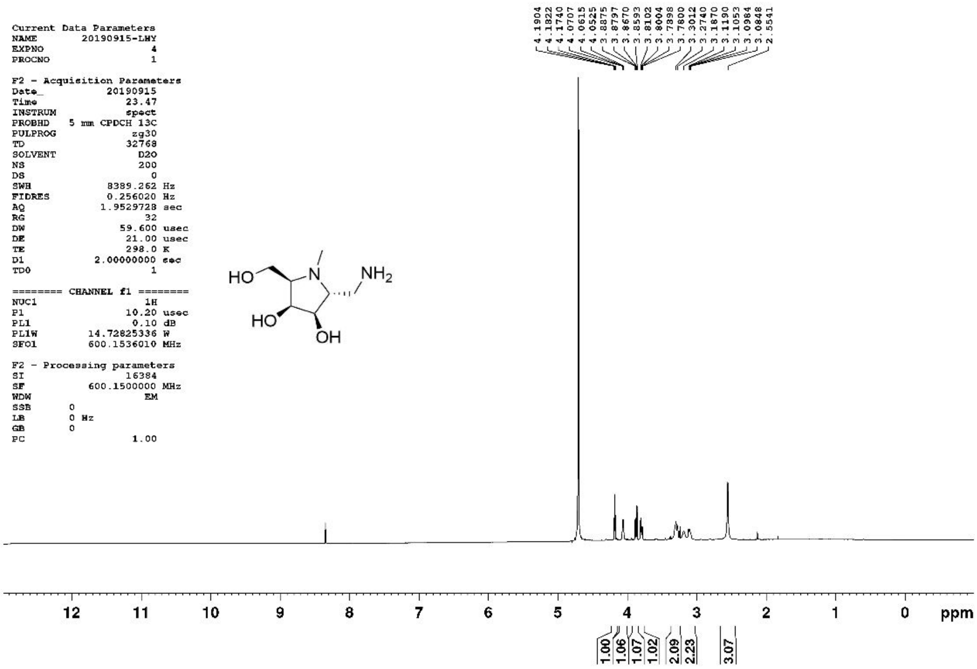

^{1}H spectrum of compound **24 (**600 MHz, D_2O)

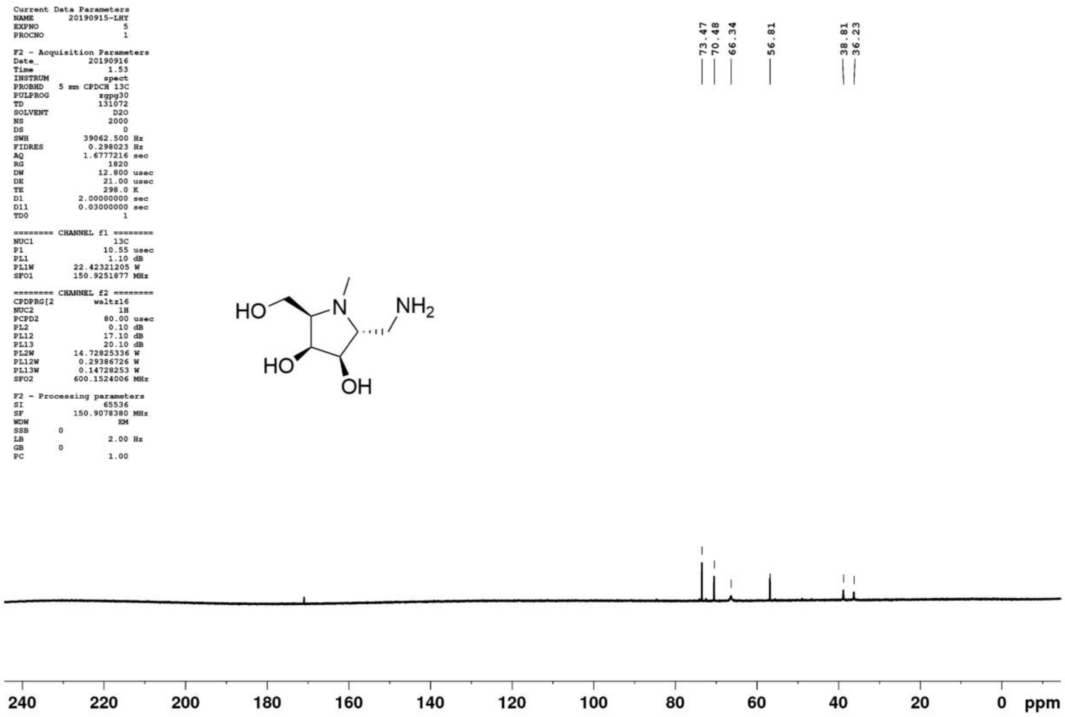

^{13}C spectrum of compound **24 (**150 MHz, D_2O)

5. Reference

(1) Niesen, F. H.; Berglund, H.; Vedadi, M. The use of differential scanning fluorimetry to detect ligand interactions that promote protein stability. *Nat. Protoc.* **2007**, *2*, 2212-2221.

(2) Li, H.-Y.; Lee, J.-D.; Chen, C.-W.; Sun, Y.-C.; Cheng, W.-C. Synthesis of (3*S*,4*S*,5*S*)-trihydroxylpiperidine derivatives as enzyme stabilizers to improve therapeutic enzyme activity in Fabry patient cell lines. *Eur. J. Med. Chem.* **2018**, *144*, 626-634.

(3) Garman, S. C.; Garboczi, D. N. The Molecular Defect Leading to Fabry Disease: Structure of Human α-Galactosidase. *J. Mol. Biol.* **2004**, *337*, 319-335.

(4) Lieberman, R. L.; D.aquino, J. A.; Ringe, D.; Petsko, G. A. Effects of pH and Iminosugar Pharmacological Chaperones on Lysosomal Glycosidase Structure and Stability. *Biochemistry* **2009**, *48*, 4816-4827.

(5) Otwinowski, Z.; Minor, W. [20] Processing of X-ray diffraction data collected in oscillation mode. In *Methods in Enzymology*, Vol. 276; Academic Press, 1997; pp 307-326.

(6) Vagin, A.; Teplyakov, A. Molecular replacement with MOLREP. *Acta Crystallogr. D Biol. Crystallogr.* **2010**, *66*, 22-25.

(7) Emsley, P.; Lohkamp, B.; Scott, W. G.; Cowtan, K. Features and development of Coot. *Acta Crystallogr. D Biol. Crystallogr.* **2010**, *66*, 486-501.

(8) Murshudov, G. N.; Skubak, P.; Lebedev, A. A.; Pannu, N. S.; Steiner, R. A.; Nicholls, R. A.; Winn, M. D.; Long, F.; Vagin, A. A. REFMAC5 for the refinement of macromolecular crystal structures. *Acta Crystallogr. D Biol. Crystallogr.* **2011**, *67*, 355-367.

(9) Williams, C. J.; Headd, J. J.; Moriarty, N. W.; Prisant, M. G.; Videau, L. L.; Deis, L. N.; Verma, V.; Keedy, D. A.; Hintze, B. J.; Chen, V. B.; et al. MolProbity: More and better reference data for improved all-atom structure validation. *Protein Sci.* **2018**, *27*, 293-315.

(10) Sugawara, K.; Tajima, Y.; Kawashima, I.; Tsukimura, T.; Saito, S.; Ohno, K.; Iwamoto, K.; Kobayashi, T.; Itoh, K.; Sakuraba, H. Molecular interaction of imino sugars with human α-galactosidase: Insight into the mechanism of complex formation and pharmacological chaperone action in Fabry disease. *Mol. Genet. Metab.* **2009**, *96*, 233-238.

(11) Gloster, T. M.; Meloncelli, P.; Stick, R. V.; Zechel, D.; Vasella, A.; Davies, G. J. Glycosidase Inhibition: An Assessment of the Binding of 18 Putative Transition-State Mimics. *J. Am. Chem. Soc.* **2007**, *129*, 2345-2354.

(12) Cheng, W.-C.; Wang, J.-H.; Li, H.-Y.; Lu, S.-J.; Hu, J.-M.; Yun, W.-Y.; Chiu, C.-H.; Yang, W.-B.; Chien, Y.-H.; Hwu, W.-L. Bioevaluation of sixteen ADMDP stereoisomers toward alpha-galactosidase A: Development of a new pharmacological chaperone for the treatment of Fabry disease and potential enhancement of enzyme replacement therapy efficiency. *Eur. J. Med. Chem.* **2016**, *123*, 14-20.

(13) Shin, S.-H.; Park, M.-H.; Byeon, J.-J.; Lee, B. I.; Park, Y.; Ko, A.-r.; Seong, M.-r.; Lee, S.; Kim, M. R.; Seo, J.; et al. A Liquid Chromatography-Quadrupole-Time-of-Flight Mass Spectrometric Assay for the Quantification of Fabry Disease Biomarker

Globotriaosylceramide (GB3) in Fabry Model Mouse. *Pharmaceutics* **2018**, *10*, 69.
(14) Nilsen, J.; Trabjerg, E.; Grevys, A.; Azevedo, C.; Brennan, S. O.; Stensland, M.; Wilson, J.; Sand, K. M. K.; Bern, M.; Dalhus, B.; et al. An intact C-terminal end of albumin is required for its long half-life in humans. *Commun. Biol.* **2020**, *3*, 181.
(15) Zhu, J.-S.; Nakagawa, S.; Chen, W.; Adachi, I.; Jia, Y.-M.; Hu, X.-G.; Fleet, G. W. J.; Wilson, F. X.; Nitoda, T.; Horne, G.; et al. Synthesis of Eight Stereoisomers of Pochonicine: Nanomolar Inhibition of β-*N*-Acetylhexosaminidases. *J. Org. Chem.* **2013**, *78*, 10298-10309.
(16) Fleet, G. W. J.; Nicholas, S. J.; Smith, P. W.; Evans, S. V.; Fellows, L. E.; Nash, R. J. Potent competitive inhibition of α-galactosidase and α-glucosidase activity by 1,4-dideoxy-1,4-iminopentitols: syntheses of 1,4-dideoxy-1,4-imino-d-lyxitol and of both enantiomers of 1,4-dideoxy-1,4-iminoarabinitol. *Tetrahedron Lett.* **1985**, *26*, 3127-3130.
(17) Saotome, C.; Kanie, Y.; Kanie, O.; Wong, C.-H. Synthesis and enzymatic evaluation of five-membered iminocyclitols and a pseudodisaccharide. *Biorg. Med. Chem.* **2000**, *8*, 2249-2261.
(18) Fleet, G. W. J.; Smith, P. W. Enantiospecific syntheses of deoxymannojirimycin, fagomine and 2*R*,5*R*-dihydroxymethyl-3*R*,4*R*-dihydroxypyrrolidine from D-glucose. *Tetrahedron Lett.* **1985**, *26*, 1469-1472.
